# An extended gene protein/products boolean network model including post-transcriptional regulation

**DOI:** 10.1186/1742-4682-11-S1-S5

**Published:** 2014-05-07

**Authors:** Alfredo Benso, Stefano Di Carlo, Gianfranco Politano, Alessandro Savino, Alessandro Vasciaveo

**Affiliations:** 1 Department of Control and Computer Engineering, Politecnico di Torino, Torino, Italy; 2 Consorzio Interuniversitario Nazionale per l'Informatica, Verres (AO), Italy

## Abstract

**Background:**

Networks Biology allows the study of complex interactions between biological systems using formal, well structured, and computationally friendly models. Several different network models can be created, depending on the type of interactions that need to be investigated. Gene Regulatory Networks (GRN) are an effective model commonly used to study the complex regulatory mechanisms of a cell. Unfortunately, given their intrinsic complexity and non discrete nature, the computational study of realistic-sized complex GRNs requires some abstractions. Boolean Networks (BNs), for example, are a reliable model that can be used to represent networks where the possible state of a node is a boolean value (0 or 1). Despite this strong simplification, BNs have been used to study both structural and dynamic properties of real as well as randomly generated GRNs.

**Results:**

In this paper we show how it is possible to include the post-transcriptional regulation mechanism (a key process mediated by small non-coding RNA molecules like the miRNAs) into the BN model of a GRN. The enhanced BN model is implemented in a software toolkit (EBNT) that allows to analyze boolean GRNs from both a structural and a dynamic point of view. The open-source toolkit is compatible with available visualization tools like Cytoscape and allows to run detailed analysis of the network topology as well as of its attractors, trajectories, and state-space. In the paper, a small GRN built around the mTOR gene is used to demonstrate the main capabilities of the toolkit.

**Conclusions:**

The extended model proposed in this paper opens new opportunities in the study of gene regulation. Several of the successful researches done with the support of BN to understand high-level characteristics of regulatory networks, can now be improved to better understand the role of post-transcriptional regulation for example as a network-wide noise-reduction or stabilization mechanisms.

## Introduction

In the last ten years the sequencing of the genome of several living organisms [1], as well as the identification and functional annotation of thousand of the proteins that these genomes encode [2,3], allowed remarkable advances in molecular biology. The identification of the genome is only the first step in understanding how cells work, and researchers are now switching to the next major challenge consisting in studying how the different actors (genes, proteins, and other cellular components) interact together and regulate each others to balance and synchronize all the biological activities of a cell [4]. The biggest methodological problem is that these type of interactions are typical of complex (or non-linear) systems, where important properties emerge from the interaction of their components, and cannot be predicted only from the study of the parts taken individually. In fact, in biological systems, decisions are reached and actions are taken by methods that are exceedingly parallel and extraordinarily integrated [5]. Complex Systems Biology aims at systematically studying complex interactions among components of biological systems, by means of theoretical instruments provided by the science of complex systems [6]. Consequently, to understand the nature of cellular functions, it is necessary to study the behavior of genes in a holistic rather than in an individual manner because the expressions and activities of genes are not isolated or independent of each other [7]. In this context, the definition of models and computational methods supporting the study of Gene Regulatory Networks (GRN) is a primary objective. GRNs are a general model, derived from the graph theory, used to represent regulatory interactions between genes, proteins, and other regulatory elements like, for example, small non-coding RNAs.

The more complex a network is, the simpler its model has to be in order to make its computational analysis feasible. Several computational approaches have been proposed and developed in literature [8] to model GRNs, and they mostly differ in the way they model the interaction between nodes: partial differential equations, ordinary differential equations, linear models [9-11], Bayesian networks [12,13], Boolean Networks [14] and Petri nets [15]. Depending on the chosen approach, the state of each network node can be considered as *discrete *or *continuos*. In the first case, each network node, i.e., genes/proteins, is supposed to assume only a small number of discrete states, avoiding intermediate expression levels. Consequently, the regulatory interactions between nodes are described by logical functions. Bayesian, Boolean and Petri networks support this approach. Instead, if the states are considered to be continuous functions in time, then their evolution is modeled by differential *rate *equations. Their punctual value is a function of the expression of the input components. Partial differential equations, (nonlinear) ordinary differential equations and linear models support this latter approach.

Each approach has limits in the size of the network and the type of computation that is able to handle. Consequently the choice of the best model depends on the type of analysis that needs to be performed. In this paper we concentrate on the study of the equilibrium states of GRNs and on the analysis of the dynamics that allow the network to evolve from an initial state to one or more of its steady states. Equilibrium states (known, in complex systems theory, as *attractors*) are particularly important because, in GRNs, they have been correlated with the gene expression profiles obtained by microarrays and other genomic experiments ( [16-19]).

On of the simplest yet effective models that can be used to study a complex network dynamics are Boolean Networks (BNs). Introduced by Kauffman [14] in 1969, they repeatedly proved successful in modeling real regulatory networks (see [4,20-25], and further references therein). A BN is a directed graph in which each node (gene) receives inputs from a fixed number of selected nodes (genes). The state of a gene is described by a Boolean variable that is active (ON, 1 value) or inactive (OFF, 0 value). The value of the state of each gene is computed by means of a Boolean function whose inputs are the state of its input nodes. Transitions between states are *deterministic*, which means that a single output state is the consequence of a given input. Although the approach seems to set a strong simplification towards reality, BNs enable to study high-level properties of a network, like its state-space, its robustness to background noise, or its behavior under different initial conditions. Recent researches suggest that also several realistic biological questions may be studied by looking at this simple Boolean formalism and in particular computing and analyzing the related network attractors (i.e., a state or a set of states towards which a system, that is moving according to its dynamic, evolves over time) [19,26,27]. However, most published models focus on the classic Gene/Protein model, neglecting other regulatory mechanisms like, for example, post-transcriptional regulation mediated by small non-coding RNA sequences such as microRNA (miRNA).

miRNA and non-coding RNA have demonstrated to play a central role in how the genome is regulated and how traits are passed on or eliminated by environmental and genetic factors.

In this paper we show how post-transcriptional regulatory interaction mediated by miRNA can be included in a Boolean Network model. We present a software tool, previously introduced in [28], able to simulate and analyze these models and to study the influence of post-transcriptional regulation on the dynamic properties of the networks like state-space, basins of attraction, and robustness. The main contribution of the paper is therefore a tool supporting an extended BN model that allows a more realistic representation of the cell regulatory activity that, in turn, allows improving the exploratory power of the BN formalism.

## Background

### Boolean networks

The attempt to model the most general aspects of gene regulatory networks dates back to the end of 1960s when Kauffman in [14] proposed a first idealized representation of a typical gene network. He modeled the regulatory interaction among genes as a directed graph in which each gene receives inputs from a fixed number of selected genes. The state of each regulatory entity, i.e., a gene, is represented as a Boolean value, either 1, representing the *activation *of the entity (e.g., a gene is expressed), or 0 representing its *inactivation *(e.g., a gene is not expressed). Connections between genes are directed, and an edge from node *x *to node *y *implies that *x *influences (activates or silences) the expression of *y*. Formally, given a set of *N *entities, such as genes, proteins etc., the *state *of the GRN is then naturally represented as a Boolean vector $\hat{X}=\left[x_{1},\cdots \;,x_{N}\right]$, that generates a space of 2*^N ^*possible states. The behavior of the state of each node *x_i _*is described using a Boolean function *f_i_*, which defines the value of the next state of *x_i _*using, as inputs, the states of its input nodes, i.e., those which directly affect its expression. Since the simulation of a BN is done in discrete time steps, the dynamics of a Boolean network modelling a regulatory system are described by:

(1) $$\hat{X}\left(t+1\right)=\hat{F}\left(\hat{X}\left(t\right)\right)$$

where $\hat{X}\left(t+1\right)$ is the next GRN state given the $\hat{F}$ vector of all functions *f_i _*that map the transition of a single node from the current state to the next one.

The transition between two states of a BN can be modeled in two ways: *asynchronously*, where each entity updates its state independently from the others, or *synchronously*, where all entities update their states together. The synchronous approach is the most widely used in literature [29,30]. In the synchronous model, a sequence of states connected by transitions forms a state-space *trajectory*. All trajectories always end into a steady state or a steady cycle. These steady (or equilibrium) states are commonly referred to as *point *or *dynamic attractors*, respectively. Point attractors consist of only one state: once the system reaches that state, it is "frozen" and no longer able to move elsewhere. On the contrary, dynamic (or periodic) attractors reveal a cyclic behavior of the system: once a trajectory falls into one of the states belonging the dynamic attractor, the system can only move between states belonging to the same attractor. For each attractor, the set of initial states that leads to it is called *basin *of attraction [31]. The analysis of the attractors characteristics (such as their size, or the size of their basin of attraction and their trajectories) are very important clues used to infer general GRN characteristics [31,32].

### Post-transcriptional modeling

**Figure 1:**
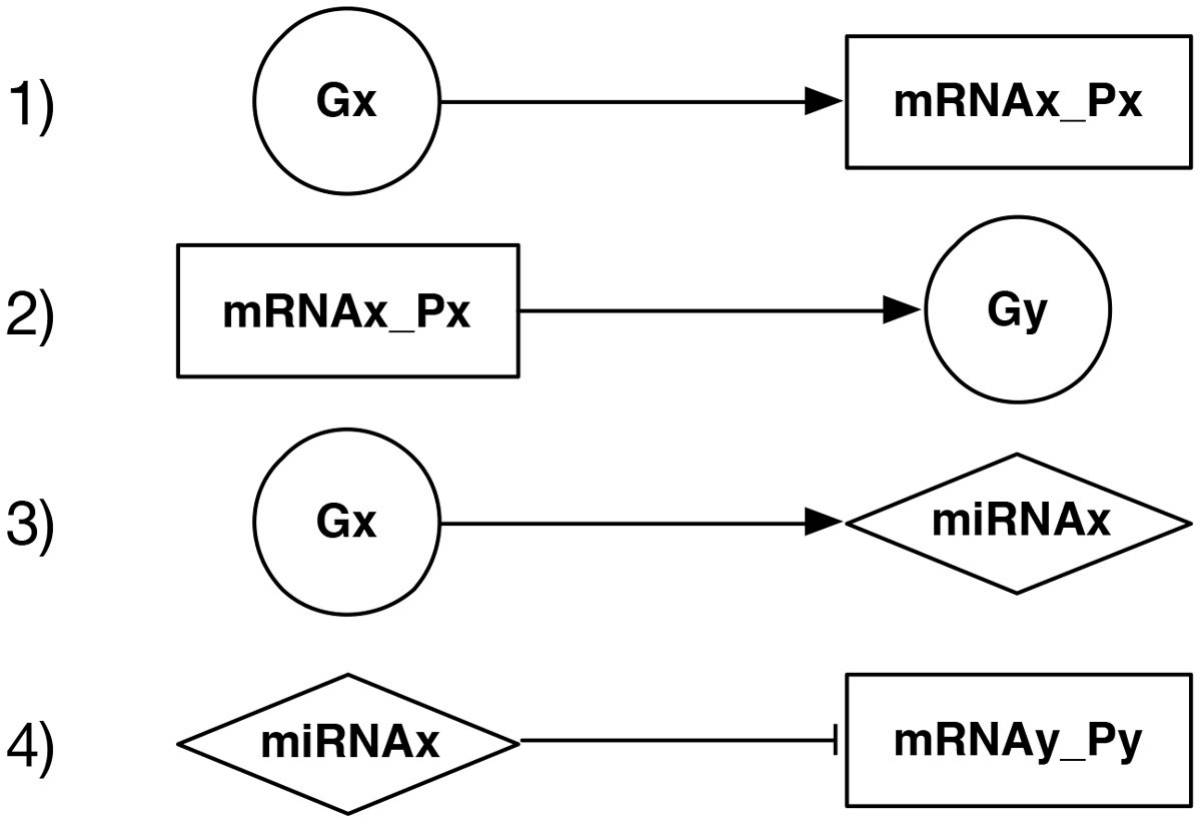
**Modeling regulatory mechanisms: 1) transcription/translation - 2) gene activation - 3) miRNA transcription - 4) post-transcriptional regulation**.

The starting point to model gene regulatory activities with Boolean Networks is the Gene Protein/Product Boolean Network model (GPBN) proposed by [32]. Differently from the previous approaches where regulatory networks were modeled using only genes, in this work the authors detail the regulatory genes' interactions by explicitly separating genes from their protein products (as separate nodes in the network). We now know that also miRNAs participate, post-transciptionally, in the regulation of almost every cellular process like, for instance, cell metabolism, signal transduction, cell differentiation, cell fate, and so on [33,34]. In the present work, with the introduction of miRNAs, we show how it is possible to include post-transcriptional regulation in the GPBN model. In general, miRNAs target mRNA molecules by interfering, using still poor understood mechanisms, with their translation, stability, or both [35]. Starting from the GPBN model, we extended the interaction between genes by explicitly introducing, as separate network nodes, also their non-coding RNA products [36,37]. In our extended GPBN model nodes are labeled in three possible ways: (1) *genes *(circular nodes), (2) *mRNA Protein *pairs (rectangular nodes), and (3) *miRNA *(rhomboidal nodes). There are consequently four possible types of edges between nodes (see Figure 1):

1. transcription/translation: an edge from a gene to a protein product; it represents the process that from the gene activation leads to the protein expression;

2. gene activation: an edge from a protein to a gene; it represents the activation of a gene by one or more protein products (Transcription Factors);

3. miRNA transcription: an edge from a host gene to a miRNA node; it means that the expression of the gene implies the transcription of miRNA molecules encoded in the DNA transcript;

4. post-transcriptional regulation: a (silencing) edge from a miRNA to a protein; it means that the protein is a miRNA target and therefore the protein translation is inhibited by the presence of the miRNA.

In order to properly model the post-transcriptional regulation mechanisms, it is necessary to carefully design the set of boolean functions that define the (next) state of each transcriptional product targeted by a miRNA node. Post-transcriptional regulation acts at mRNA level, hence, considering the final protein product, it has higher priority compared to gene expression activity. In terms of boolean networks, it can be modeled by placing the miRNA expression state in Boolean AND with the mRNA expression state.

As already mentioned in [32], the introduction of gene products also requires to take into account the time each product is synthesized in the BN timeline of states evolution. Since the update of inner node values is synchronous [31], the synthesis products require *s *time steps to be ready (expressed). In the same way, once a gene is no longer *expressed*, its related products are *silenced *after *d *time steps. In our work synthesis and decay times (*s *and *d *) are defined as unitary for all the entities so that, if a given gene is turned ON/OFF at time *t*, all its products will be accordingly turned ON/OFF at time *t *+ 1. The *lifecycle *of miRNAs is the same as all other gene products.

Although the introduction of miRNAs activity into the BN makes it possible to include their post-transcriptional effects into the dynamics of the system, it is not enough to properly model the whole post-transcriptional activity. At this point, not all the states are biologically valid. Even though, if well designed, the dynamics of the GRN makes it impossible to evolve into a biologically illegal state, there is no guarantee that an illegal state is not used as the initial state when simulating the network.

To avoid illegal states, the description of the BN is expanded to include a set of conditions identifying all illegal states of the network. These conditions are represented by an additional set of Boolean equations that must be evaluated every time an initial state of the network is considered. Using boolean basics, a state is considered *legal *if all conditions return zero, *illegal *otherwise. As an example, let us consider a gene Gx and its related protein *mRNAx_Px*. The protein can be synthesized only if the related gene has been expressed. So, any state in which *mRNAx_Px *is equal to 1 (expressed), while Gx is equal to 0 (not expressed) is considered as an illegal state.

## Methods

There are a number of software applications for experimenting with BNs [38-40]. Some of them are too narrow in scope, inefficient or difficult to customize. Since our primary goal was to integrate the post-transcriptional BN model into a flexible programming environment, we modified the Boolean Network Toolkit (BNT) presented in [30], which was very easy to customize thanks to the C++ open implementation of its core engine. The resulting toolkit, named Extended BN Toolkit (EBNT), is under development under GPL license and available at http://www.testgroup.polito.it/index.php/bio-menu-tools/item/208-boolean-regulatory-network-simulator. The code is supported by the BOOST C++ cross platform and multithread library [41], which allows high computational performances and code portability.

The input GRN description is a simple text file describing the nodes names, types, and interconnections. A separate text file contains the functional constraints described in the previous section. After being loaded in the BNT core, a boolean network is represented as a direct graph using adjacent lists. Each node is represented as a data structure containing different information such as the node name, type (e.g., gene, protein or miRNA), and other parameters useful to characterize the node from both a functional and graphical point of view. Any network output file is in xgmml format, an xml-like open format compatible with several visualization tools like Cytoscape [42], a flexible and open-source software platform for visualizing complex networks. This solution also allows to use the outputs of our tool with the many available Cytoscape plugins for network enrichment and topological analysis.

**Figure 2:**
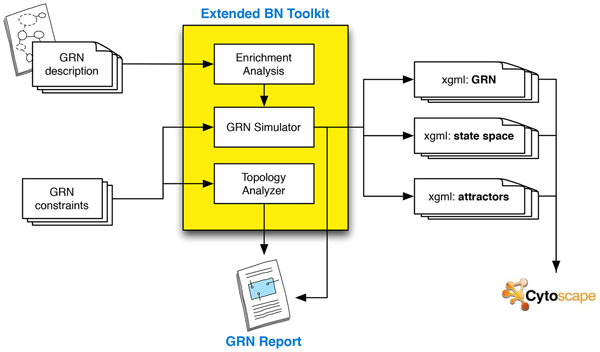
**The EBNT conceptual architecture: the Network Enrichment module, the GRN Simulator, and the Topology Analyzer**.

The EBNT is currently composed of three main modules (see Figure 2): a Network Enrichment module designed to help the researcher in modeling a more realistic network, a GRN Simulator, implemented to run dynamic analysis on the network, and a Topology Analyzer, able to compute static analysis on the considered GRN network.

**Figure 3:**
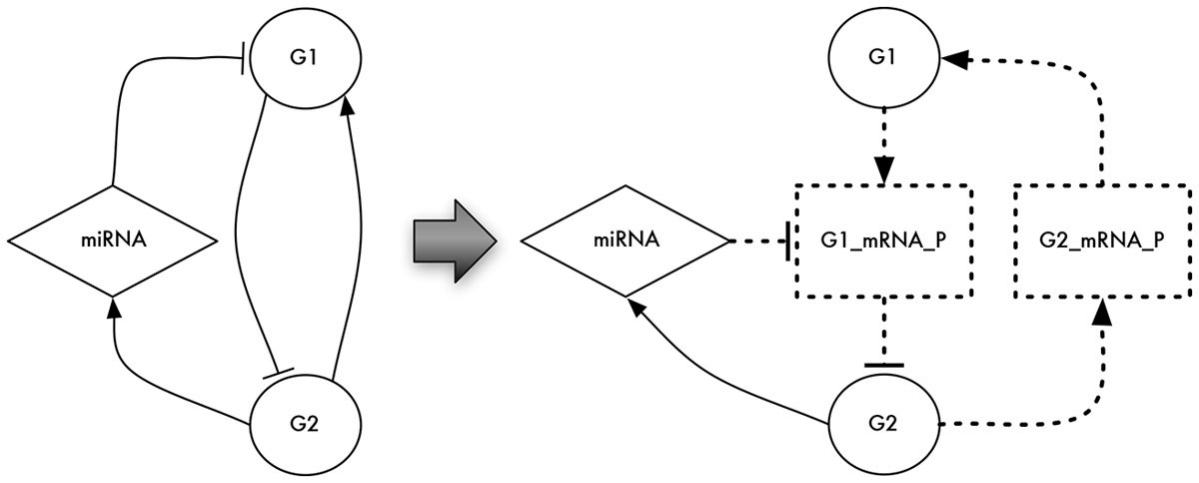
**The post-transcriptional BN enrichment process is able to verify the correct representation of the post-transcriptional mechanism**. If the BN includes a miRNA node targeting a gene instead of a protein, a new protein node is generated. This node is then connected to the parent gene and the miRNA target becomes the protein instead of the gene.

**Figure 4:**
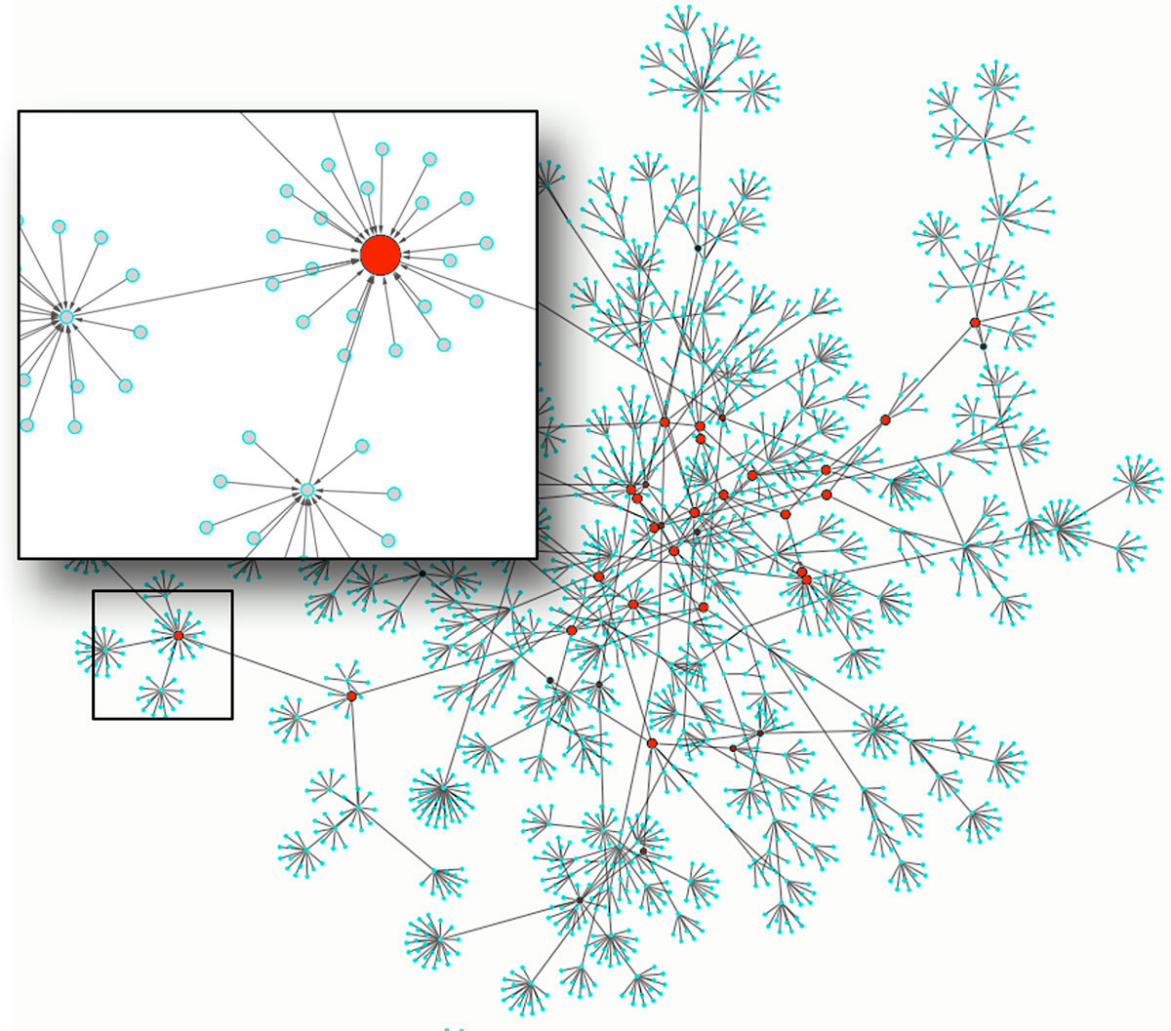
**Cytoscape rendering of the state-space of an example network where it is possible to identify the basins of attraction, and the corresponding network attractors (red nodes)**.

**Figure 5:**
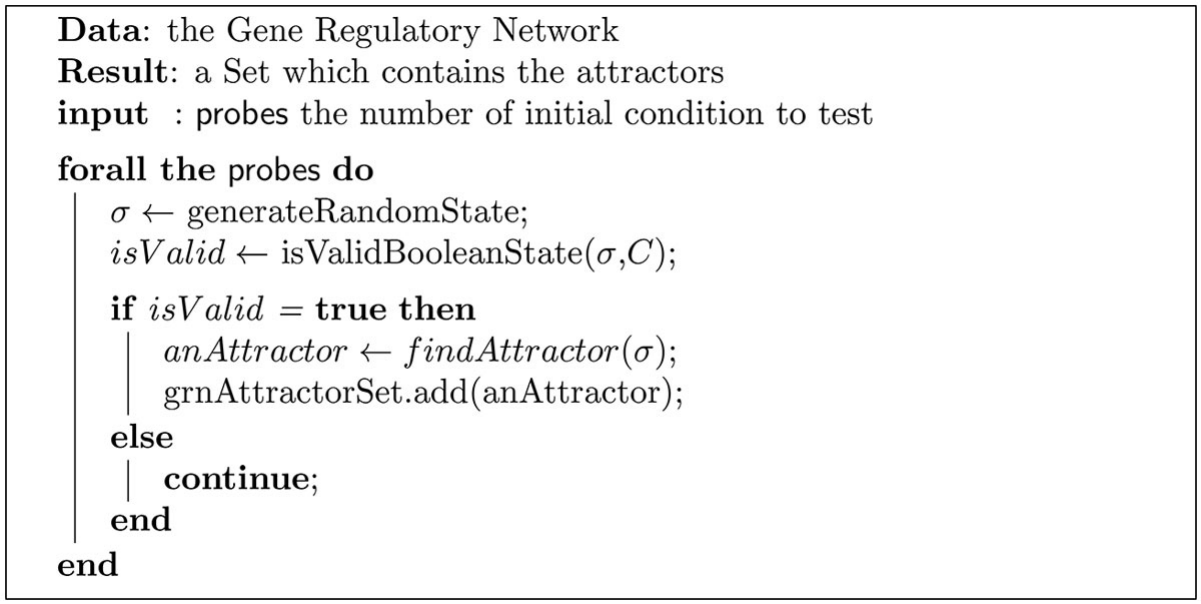
**Pseudocode of the attractors search algorithm, modified to work with the extended BN model**. The attractor search procedure is an iterative process involving *probes *network simulations. *P robes *is the number of initial states from which the network is evolved to identify its attractors.

• Network Enrichment: this module is designed to create a more reliable representation of the network. It is particularly useful when the goal is to analyze a realistic regulatory network (and not to analyze random networks with topological characteristics resembling the ones of real biological networks). The module is able to verify the correct representation of the post-transcriptional mechanism. According to [43], if the transcription/translation is active, mRNAs/proteins are synthesized in one time step. Thus, if the BN includes a miRNA node targeting a gene instead of a protein, a new protein node is generated. This node is then connected to the parent gene and the miRNA target becomes the protein instead of the gene. Also, all gene outgoing edges (that represent the synthesis of gene products) are re-arranged accordingly. The final resulting network still respects the assumption that transcription factors and proteins, undergoing post-transcriptional modification, decay in one time step if the corresponding mRNA is not anymore expressed [43]. Figure 3 shows the BN post-transcriptional enrichment process. The second task of this module (currently under development also as a standalone Cytoscape 3.0 plugin) is to analyze the post-transcriptional interactions modeled in the network against available online repositories. Existing miRNA targets can be verified or additional targets suggested in order to make the network as realistic and complete as possible (at least according to the available data).

• GRN Simulator: the simulator has been designed to compute the network dynamics by identifying its attractors and by mapping all network simulation trajectories into a state-space diagram. A state-space diagram is one of the only ways to provide a graphical output of the set of possible states of a network. Ideally, a realistic state-space representation should be done on an N-dimension diagram, where N is the number of network nodes. Since this is graphically unfeasible, a 2D graph representation is used, where each network state is represented by a node, and two nodes are connected if they represent two consecutive states in the network evolution (e.g., if node A is connected to node B, it means that the network can directly pass from state A to state B). In a synchronous network like the ones modeled in this work, in the state-space diagram it is possible to identify separate sets of connected nodes. Each set represents a set of trajectories (state sequences) ending in the same point or periodic attractor. Each separate set of connected nodes is called "basin of attraction", and the analysis of its size and characteristics can provide interesting additional clues on the original network dynamics (like robustness and resistance to gene expression noise). Figure 4 shows the Cytoscape rendering of the state-space of an example network where it is possible to identify the basins of attraction, and the corresponding network attractors (red nodes). To provide a better exploratory capability to the user, we also implemented a *Cytoscape plugin *that allows to easily navigate the network state-space and to identify trajectories and states of interest, and to zoom into each state or attractor to analyze the corresponding detailed network configuration in terms of expressed and silenced nodes. Figure 5 shows the overall attractor search process, modified to work with the extended BN model. The attractor search procedure is an iterative process involving *probes *network simulations. *P robes *is the number of initial states from which the network is evolved to identify its attractors. If the number of network nodes (*N*) makes considering all possible (2*^N^*) initial state candidates computationally unacceptable, then a reduced number of random initial state candidates can be selected. At each iteration, a new initial state is selected among the set of candidates, and its validity against the transcriptional and post-transcriptional constraints is verified. If the state is marked as *legal*, the network dynamics are simulated to search for an attractor on the selected trajectory; if instead at least one of the constraints is true, it means the given state is not valid and it needs to be discarded.

• Topology Analyzer: this module, under continuous development, integrates custom and freely available Cytoscape plugins to perform several topological analysis on both the original regulatory network and the state-space networks obtained during the simulations. It can be used, for example, to identify network bottlenecks and hubs, or measure the *network diameter *(the maximum distance between any two nodes in a network), the network *sensitivity *and *robustness *[44], or the *clustering coefficient *(the percentage of existing links among the neighborhood of one node). An useful capability is the ability to merge two state-spaces to highlight their differences in terms of attractors and simulation trajectories (see an example in the Results section).

## Results and discussion

To demonstrate the performances of the presented toolset, we performed two set of experiments. In the first, designed to better profile its scalability and the manageable network size, we applied the attractors search algorithm on a set of artificially generated GRNs that include post-transcriptional regulation mechanisms. The second experiment was performed to give readers an example of the type of analysis that the tool allows to perform on a small but realistic regulatory network involving the mTOR pathway.

## Performance characterization

**Figure 6:**
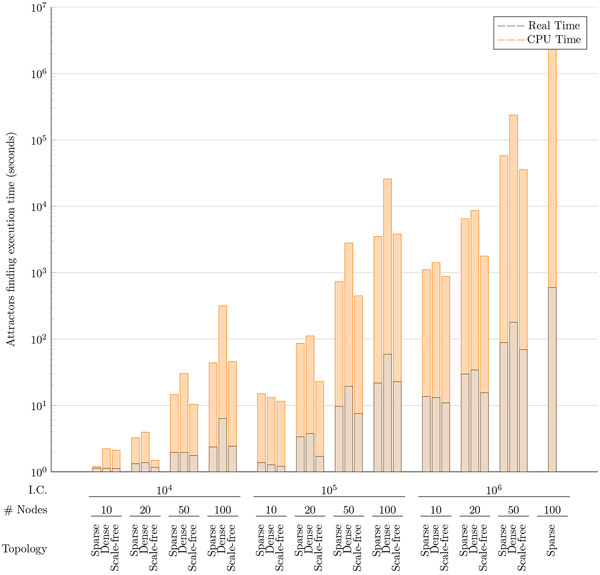
**Time performances of the network attractors search algorithm**. Experiments were performed on three different network types (Sparse, Dense, and Scale Free) with different number of nodes (10, 20, 50, and 100). For each network experiments were repeated with 10K, 100K, and 1M initial random states. Each bar represents the average of 10 experiments with the same number of random initial states; Reference Hardware: Intel(R) Core(TM) i7-2670QM CPU @ 2.20GHz ; 8GB RAM 1333MHz.

Experiments were performed on a 8-core workstation featuring multithreading programming on three different network types:

• *dense networks*: each node has an average in/out degree (number of input/output edges) equal to 25;

• *sparse networks*: each node has an average in/out degree equal to 5;

• *scale-invariant networks*: each node has an average in/out degree equal to 5; a select number of nodes are hubs with in/out degree greater than 25;

For each class we generated four networks with increasing number of nodes: 10, 20, 50, and 100 nodes. For each network we applied the attractor search algorithm considering increasing number of initial probes, and each experiment has been repeated 10 times to account for the casualty of the initial state generation (Figure 6). The attractor search algorithm is a multithreaded function able to explore the network states exploiting all available microprocessor cores. In this way, the search is 8-times faster then in a single-core implementation. The only actual limitation is the memory consumption since the search complexity considerably increases with the number of nodes and edges of the network. With 8GB of available RAM we noticed a performance breakdown when the GRN configuration reaches 100 nodes with an average number of incoming edges per node higher than 29. The results reported in Figure 6 show that attractors of networks up to 100 nodes are computed in a very reasonable time (Real Time is lower than CPU Time thanks to the multithreading approach).

### Simulation of the mTOR pathway

**Figure 7:**
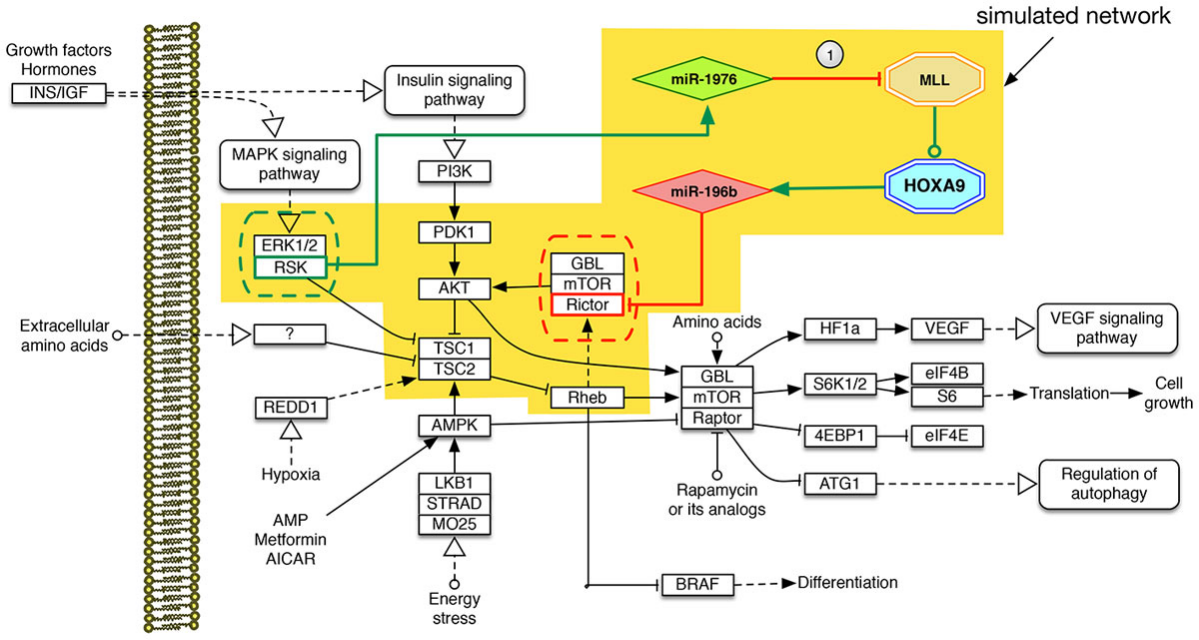
**The mTOR default network (empty nodes) enhanced with the PPL (filled nodes)**. For simulating misbehaviors we removed the edge marked as 1, thus impairing the miR-1976 post-trascriptional regulation of MLL. This acts as common pathological rearrangements of MLL, which lead to several malignancies.

To show an example of how the presented tool can provide biologically meaningful information, we performed a set of simulations on a slightly modified version of the mTOR pathway (#hsa04150 - http://www.genome.jp/kegg-bin/show_pathway?hsa04150), whose dysregulation is considered as a key factor in several malignancies. The mTOR signaling pathway combines the signals produced by several upstream pathways, like insulin, growth factors and amino acids [45], as well as cellular nutrition, energy levels and redox status [46]. Targeting this pathway with selected drugs showed promising results in the treatment of several types of cancer (e.g., leukemia, glioblastoma, myelodysplasia breast, hepatic and pancreatic [47,48]), in which the mTOR pathway has been found dysregulated [49]. In the following experiments we used a subset of the mTOR pathway of Figure 7; the pathway contains two main complexes: mTORC1 (composed of mTOR, GBL, and RAPTOR), and mTORC2 (composed of mTOR, GBL, and RICTOR). Our experiment focused on the mTORC2 complex (regulated by insulin, growth factors, serum, and nutrient levels [50]). In literature, several works suggest that the RICTOR gene is a possible responsible for metastasis and inhibition of growth factors [51]. When down-regulated, it seem to reduce the phosphorylation of AKT and PKC that impairs the differentiation of Th2 cells. These cells are important because they produce cytokines like IL-4, IL-5, IL-10, and IL-13, responsible for several protective functions as antibody production, eosinophil activation, and inhibition of several macrophage functions [52]. In a previous work ([53]), we hypothesized that the down-regulation of RICTOR could be caused by a cascading effect caused by the disruption of a protective feedback loop involving the RSK gene that hosts an intragenic miRNA (miR-1976) which is able to inhibit the expression of the MLL transcription factor. MLL is responsible for the transcription of HOXA9, which hosts miR-196b that, if expressed, would target RICTOR dysregulating the mTORC2 complex.

The analysis of this complex regulatory mechanism seemed an excellent candidate to test the proposed EBNT toolkit.

To setup the experiments we designed two versions of the yellow shaded portion of the mTOR pathway of Figure 7: the first version is fully working, whereas in the second we deleted the down-regulatory edge between miR-1976 and MLL (see Figure 7, edge marked "1"). Such modification mimics a set of known pathological MLL translocations (t(4;11), t(11;19), t(9;11) and t(1;11)), which may impair the inhibition capability of miR-1976. In fact, the MLL translocation may cause modifications in its miRNAs binding sites, further causing the ectopic expression of miR-196b ( [54]). For the simulated pathway to be active, the RSK/ERK and PDK1 (driven respectively by interferon and insulin) have to be expressed. Moreover, to see the effect of the regulatory loop, MLL has to be expressed; in this way it is possible to see the effect of miR1976 that, if working correctly, is able to inhibit the translation of the MLL mRNA into the MLL protein, thus blocking the expression of HOAX9 and of its intragenic miRNA miR196b that is then not able to down-regulate RICTOR.

**Figure 8:**
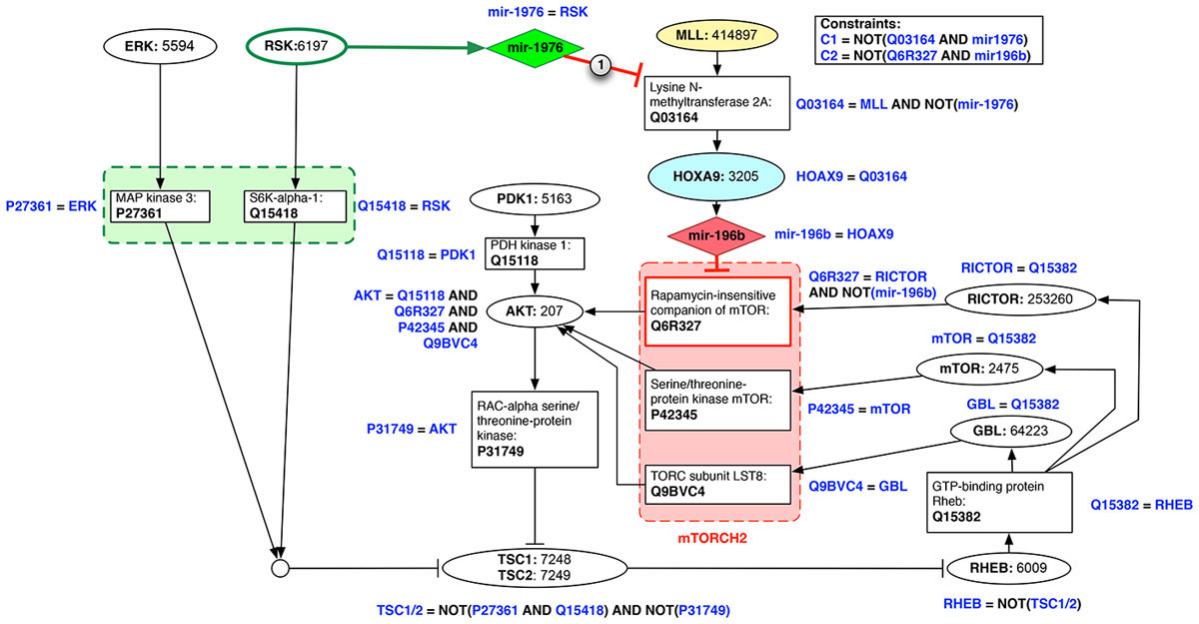
**The actual simulated network(s) with all the next-state boolean functions and the constraints used to validate the initial probes**.

After modeling both networks, we run the Network Enrichment module to add, for each gene, their related proteins and possible co-transcribed miRNAs; the final result, containing 22 nodes, is shown in Figure 8. For each node of the network the figure reports its name (the UNIPROT Id in case of proteins) and the implemented boolean function used, during the simulation, to compute the value of the next state of the node. In the top-right corner it is also possible to see the constraints applied to validate the initial random *probes*, as explained in the Methods section. In the figure is also evident the silencing edge (marked "1") from mir1976 to the MLL protein (Q03164), which is missing in the *Faulty *version of the network.

The simulation of the *Control *network allowed to identify 92 attractors, whereas the simulation of the *Faulty *network reported 73 attractors. In both experiments we exhaustively simulated all the possible 222 probes (initial states), and the simulations took less than 243 seconds for both networks (see Table 1).

**Table 1:** Summary of the mTOR pathway simulations: number of nodes, attractors, and execution time for the fully functional (Control) and modified (Faulty) network

	Control	Faulty
# nodes	22	22
# attractors	92	73
Time	242.37	241.12

In the Additional file 1 and Additional file 2 it is possible to see all of the identified point and periodic attractors, including the number of initial states that led to each of them (Hits).

Since we did not set any constraint on the value of the nodes that do not have any input node (those nodes that are not controlled by any other node), some of the identified attractors are not biologically significant for the experiment because they correspond to states where neither the pathway nor the regulatory loop are activated.

Given the set of attractors of the *Control *network, we focused on the first one, because it describes a stable state in which both the pathway and the regulatory loop are fully activated and is compatible with the information available from the literature concerning the correct expression of the mTOR pathway: the activating signals of the mTOR pathway (RSK/ERK and PDK1), all subunits of mTORC2 complex (GBL, mTOR, and RICTOR), and both AKT and RHEB are expressed; in this context, even if MLL is expressed, we have the malignant regulatory path composed by the MLL protein, HOXA9, and miR-196b blocked thanks to the protection of miR-1976 that, co-expressed by RSK, is able to inhibit the translation of the MLL protein that would activate the regulatory cascade that would eventually down-regulate RICTOR. Looking at the attractors of the *Faulty *network, again only the fist attractor corresponds to a situation in which both the pathway and the regulatory loop are activated (MLL, RSK/ERK, and PDK1 expressed). This time, as expected, the absence of a single regulatory edge, leads to the complete impairment of the mTORC2 complex. The malignant MLL's cascade, without the protective binding of miR-1976, expresses miR-196b, which interferes with the RICTOR protein translation, so inhibiting the mTORC2 complex formation. As already said RICTOR inhibition and the resulting mTORC2 knock-off, are two effects of the reduced phosphorylation of AKT and the impaired differentiation of Th2 cells [52].

The simulations results seem compatible with the data available in literature; they highlight the importance of MLL, the HOXA cluster (HOXA9), and both miR-196b and miR-1976, as presented by Schotte at al. [55,56] regarding Acute Lymphoblastic Leukemia (ALL). Also Popovic et al. [54] suggest that *"miR-196b function is necessary for MLL fusion-mediated immortalization and it may justify the fact that the mTOR pathway protects itself (with miR1976) by not allowing its expression. Similarly, the same work shows that the level of miR-196b is decreased up to 14-fold in the absence of MLL, thus confirming the down-regulatory role of miR-1976 on MLL"*.

**Figure 9:**
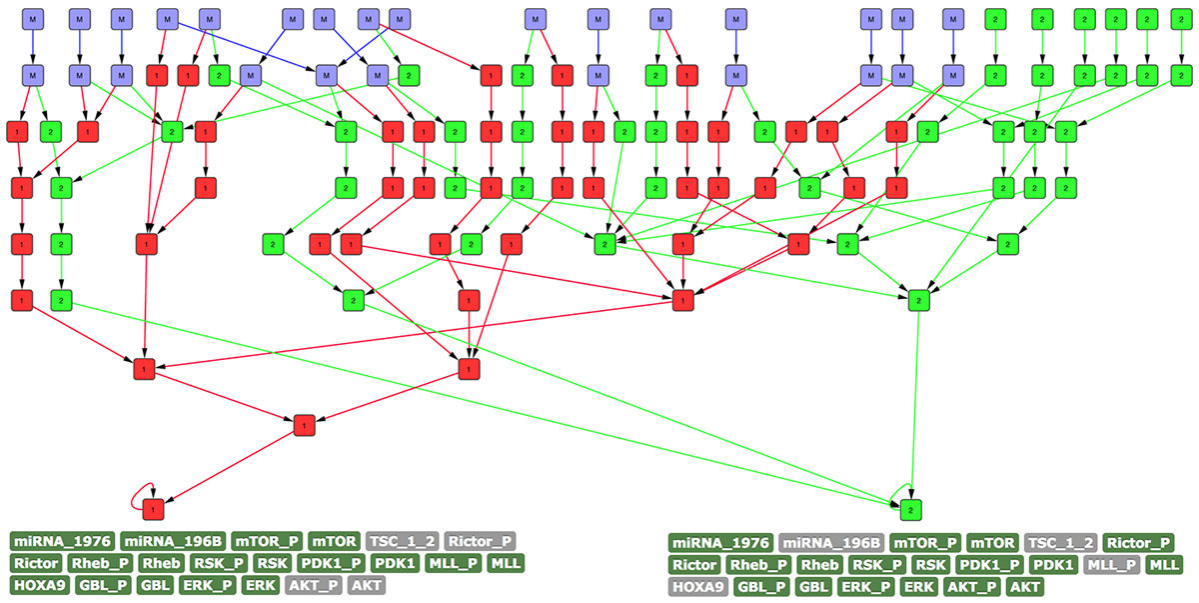
**State-space comparison between default and modified mTOR pathway**. Nodes in green (labeled "2") refer to states belonging to the default pathway, in red (labeled "1") states of the network without the protective edge, and in blue (labeled "M") the set of common states between the two state-spaces.

To better visualize the different behavior of the two networks, we run another experiment in which we used a reduced amount of probes (2000), and added a set of constraints that forced the expression of ERK/RSK, PDK1, and MLL. In this case we obtained only one attractor for each network, exactly corresponding to the ones discussed previously in this section. As an additional step, we used one of the latest functionalities of the Topology Analyzer to merge the two state-space diagrams obtained by the simulations. The result is presented in Figure 9: the nodes marked in green (labeled "2") correspond to states of the default network; the nodes marked in red (labeled "1") are states which belong to the network without the protective edge; nodes marked in blue (labeled "M") are the set of states that are common in the two networks (theoretically the two networks should have the same 222 states, but in this simulation we used 2000 random probes and therefore some of the initial states may be different between the two networks). Interestingly, all the common states ("M") are at the top of the resulting merged state-space diagram. However, after no more than two transitions, the control and faulty network trajectories appear completely disjunct, resulting in the two previously discussed attractors. This is a good clue of how deeply the pathway may be influenced by a single modification (i.e., MLL rearrangements modeled by a single edge deletion), which eventually leads to distinct attractors describing completely different phenotypes.

## Conclusions and future work

The BNT presented in this paper is an important step towards a more realistic analysis of the high-level functional and topological characteristics of GRNs. Resorting to the tool facilities, such as multicore implementation and support for common input/output formats, the dynamics of real networks of significant size can be analyzed. Thanks to the extended model that includes post-transcriptional regulation, exciting new research scenarios are opening up because the EBNT offers now a way not only to simulate a realistic size network, but also to gather new insights on the role of miRNAs from a functional as well as structural point of view. Our current efforts are geared toward verifying and better understanding the role of post-transcriptional regulation (and miRNAs in particular) as network-wide noise-reduction or stabilization mechanism.

## List of abbreviations used

GRN - Gene Regulatory Network

BN - Boolean Network

GPBN - Gene Protein/Product Boolean Network

BNT - Boolean Network Toolkit

EBNT - Extended Boolean Network Toolkit

## Authors' contributions

AB, SDC, GP and AS planned the study, participated in its design and coordination, and wrote the manuscript. AV implemented the model in software and ran the experiments. All authors read and approved the final manuscript.

## Competing interests

The authors declare that they have no competing financial interests or other conflicts of interest.

## Supplementary Material

Additional File 1 Additional file 1 contains the whole list of attractors for the default mTOR network Click here for file

Additional File 2 Additional file 2 contains the whole list of attractors for the mTOR network with deleted edge between miR-1976 and MLL Click here for file

## Declarations

Publication funding for this article has come from Grant No. CUP B15G13000010006 awarded by the Regione Valle d'Aosta for the project: "Open Health Care Network Analysis" and by the Italian Ministry of Education, University and Research (MIUR) (Project PRIN 2010, MIND).

This article has been published as part of *Theoretical Biology and Medical Modelling *Volume 11 Supplement 1, 2014: Selected articles from the 1st International Work-Conference on Bioinformatics and Biomedical Engineering-IWBBIO 2013. The full contents of the supplement are available online at http://www.tbiomed.com/supplements/11/S1.
