# Supplementary material for: An extended gene protein/products boolean network model including post-transcriptional regulation
Source: Theor Biol Med Model. 2014 May 7;11(Suppl 1):S5. doi: 10.1186/1742-4682-11-S1-S5 (PMC4108923; doi:10.1186/1742-4682-11-S1-S5)
Supplement: Additional File 1 — contains the whole list of attractors for the default mTOR network [file 1742-4682-11-S1-S5-S1.PDF]

Attractors Search Simulation

Description:

mTOR exhaustive attractors search (Default network)

Attractor Number	Hits	Data
92	31104	Basin Dimension Percentual: **24.33**
		**miRNA_1976 miRNA_196B mTOR_P mTOR TSC_1_2 Rictor_P Rictor Rheb_P Rheb RSK_P RSK PDK1_P PDK1 MLL_P MLL HOXA9 GBL_P GBL ERK_P ERK AKT_P AKT**
90	20736	Basin Dimension Percentual: **16.22**
		**miRNA_1976 miRNA_196B mTOR_P mTOR TSC_1_2 Rictor_P Rictor Rheb_P Rheb RSK_P RSK PDK1_P PDK1 MLL_P MLL HOXA9 GBL_P GBL ERK_P ERK AKT_P AKT**
88	15552	Basin Dimension Percentual: **12.17**
		**miRNA_1976 miRNA_196B mTOR_P mTOR TSC_1_2 Rictor_P Rictor Rheb_P Rheb RSK_P RSK PDK1_P PDK1 MLL_P MLL HOXA9 GBL_P GBL ERK_P ERK AKT_P AKT**
46	10368	Basin Dimension Percentual: **8.11**
		**miRNA_1976 miRNA_196B mTOR_P mTOR TSC_1_2 Rictor_P Rictor Rheb_P Rheb RSK_P RSK PDK1_P PDK1 MLL_P MLL HOXA9 GBL_P GBL ERK_P ERK AKT_P AKT**
87	10368	Basin Dimension Percentual: **8.11**
		**miRNA_1976 miRNA_196B mTOR_P mTOR TSC_1_2 Rictor_P Rictor Rheb_P Rheb RSK_P RSK PDK1_P PDK1 MLL_P MLL HOXA9 GBL_P GBL ERK_P ERK AKT_P AKT**
70	7776	Basin Dimension Percentual: **6.08**
		**miRNA_1976 miRNA_196B mTOR_P mTOR TSC_1_2 Rictor_P Rictor Rheb_P Rheb RSK_P RSK PDK1_P PDK1 MLL_P MLL HOXA9 GBL_P GBL ERK_P ERK AKT_P AKT**
44	5184	Basin Dimension Percentual: **4.06**
		**miRNA_1976 miRNA_196B mTOR_P mTOR TSC_1_2 Rictor_P Rictor Rheb_P Rheb RSK_P RSK PDK1_P PDK1 MLL_P MLL HOXA9 GBL_P GBL ERK_P ERK AKT_P AKT**

**45**	**5184**	Basin Dimension Percentual: **4.06**
		**miRNA_1976 miRNA_196B mTOR_P mTOR TSC_1_2 Rictor_P Rictor Rheb_P Rheb RSK_P RSK PDK1_P PDK1 MLL_P MLL HOXA9 GBL_P GBL ERK_P ERK AKT_P AKT**
**69**	**5184**	Basin Dimension Percentual: **4.06**
		**miRNA_1976 miRNA_196B mTOR_P mTOR TSC_1_2 Rictor_P Rictor Rheb_P Rheb RSK_P RSK PDK1_P PDK1 MLL_P MLL HOXA9 GBL_P GBL ERK_P ERK AKT_P AKT**
**51**	**3836**	Basin Dimension Percentual: **3.00**
		**miRNA_1976 miRNA_196B mTOR_P mTOR TSC_1_2 Rictor_P Rictor Rheb_P Rheb RSK_P RSK PDK1_P PDK1 MLL_P MLL HOXA9 GBL_P GBL ERK_P ERK AKT_P AKT**
		**miRNA_1976 miRNA_196B mTOR_P mTOR TSC_1_2 Rictor_P Rictor Rheb_P Rheb RSK_P RSK PDK1_P PDK1 MLL_P MLL HOXA9 GBL_P GBL ERK_P ERK AKT_P AKT**
		**miRNA_1976 miRNA_196B mTOR_P mTOR TSC_1_2 Rictor_P Rictor Rheb_P Rheb RSK_P RSK PDK1_P PDK1 MLL_P MLL HOXA9 GBL_P GBL ERK_P ERK AKT_P AKT**
		**miRNA_1976 miRNA_196B mTOR_P mTOR TSC_1_2 Rictor_P Rictor Rheb_P Rheb RSK_P RSK PDK1_P PDK1 MLL_P MLL HOXA9 GBL_P GBL ERK_P ERK AKT_P AKT**
		**miRNA_1976 miRNA_196B mTOR_P mTOR TSC_1_2 Rictor_P Rictor Rheb_P Rheb RSK_P RSK PDK1_P PDK1 MLL_P MLL HOXA9 GBL_P GBL ERK_P ERK AKT_P AKT**
		**miRNA_1976 miRNA_196B mTOR_P mTOR TSC_1_2 Rictor_P Rictor Rheb_P Rheb RSK_P RSK PDK1_P PDK1 MLL_P MLL HOXA9 GBL_P GBL ERK_P ERK AKT_P AKT**
		**miRNA_1976 miRNA_196B mTOR_P mTOR TSC_1_2 Rictor_P Rictor Rheb_P Rheb RSK_P RSK PDK1_P PDK1 MLL_P MLL HOXA9 GBL_P GBL ERK_P ERK AKT_P AKT**
**24**	**2592**	Basin Dimension Percentual: **2.03**
		**miRNA_1976 miRNA_196B mTOR_P mTOR TSC_1_2 Rictor_P Rictor Rheb_P Rheb RSK_P RSK PDK1_P PDK1 MLL_P MLL HOXA9 GBL_P GBL ERK_P ERK AKT_P AKT**
**43**	**2592**	Basin Dimension Percentual: **2.03**
		**miRNA_1976 miRNA_196B mTOR_P mTOR TSC_1_2 Rictor_P Rictor Rheb_P Rheb RSK_P RSK PDK1_P PDK1 MLL_P MLL**

		**HOXA9 GBL_P GBL ERK_P ERK AKT_P AKT**
**53**	**2556**	Basin Dimension Percentual: **2.00**
		**miRNA_1976 miRNA_196B mTOR_P mTOR TSC_1_2 Rictor_P Rictor Rheb_P Rheb RSK_P RSK PDK1_P PDK1 MLL_P MLL HOXA9 GBL_P GBL ERK_P ERK AKT_P AKT**
		**miRNA_1976 miRNA_196B mTOR_P mTOR TSC_1_2 Rictor_P Rictor Rheb_P Rheb RSK_P RSK PDK1_P PDK1 MLL_P MLL HOXA9 GBL_P GBL ERK_P ERK AKT_P AKT**
		**miRNA_1976 miRNA_196B mTOR_P mTOR TSC_1_2 Rictor_P Rictor Rheb_P Rheb RSK_P RSK PDK1_P PDK1 MLL_P MLL HOXA9 GBL_P GBL ERK_P ERK AKT_P AKT**
		**miRNA_1976 miRNA_196B mTOR_P mTOR TSC_1_2 Rictor_P Rictor Rheb_P Rheb RSK_P RSK PDK1_P PDK1 MLL_P MLL HOXA9 GBL_P GBL ERK_P ERK AKT_P AKT**
		**miRNA_1976 miRNA_196B mTOR_P mTOR TSC_1_2 Rictor_P Rictor Rheb_P Rheb RSK_P RSK PDK1_P PDK1 MLL_P MLL HOXA9 GBL_P GBL ERK_P ERK AKT_P AKT**
		**miRNA_1976 miRNA_196B mTOR_P mTOR TSC_1_2 Rictor_P Rictor Rheb_P Rheb RSK_P RSK PDK1_P PDK1 MLL_P MLL HOXA9 GBL_P GBL ERK_P ERK AKT_P AKT**
		**miRNA_1976 miRNA_196B mTOR_P mTOR TSC_1_2 Rictor_P Rictor Rheb_P Rheb RSK_P RSK PDK1_P PDK1 MLL_P MLL HOXA9 GBL_P GBL ERK_P ERK AKT_P AKT**
**72**	**1864**	Basin Dimension Percentual: **1.46**
		**miRNA_1976 miRNA_196B mTOR_P mTOR TSC_1_2 Rictor_P Rictor Rheb_P Rheb RSK_P RSK PDK1_P PDK1 MLL_P MLL HOXA9 GBL_P GBL ERK_P ERK AKT_P AKT**
**47**	**1760**	Basin Dimension Percentual: **1.38**
		**miRNA_1976 miRNA_196B mTOR_P mTOR TSC_1_2 Rictor_P Rictor Rheb_P Rheb RSK_P RSK PDK1_P PDK1 MLL_P MLL HOXA9 GBL_P GBL ERK_P ERK AKT_P AKT**
		**miRNA_1976 miRNA_196B mTOR_P mTOR TSC_1_2 Rictor_P Rictor Rheb_P Rheb RSK_P RSK PDK1_P PDK1 MLL_P MLL HOXA9 GBL_P GBL ERK_P ERK AKT_P AKT**
		**miRNA_1976 miRNA_196B mTOR_P mTOR TSC_1_2 Rictor_P Rictor Rheb_P Rheb RSK_P RSK PDK1_P PDK1 MLL_P MLL HOXA9 GBL_P GBL ERK_P ERK AKT_P AKT**

		**miRNA_1976 miRNA_196B mTOR_P mTOR TSC_1_2 Rictor_P Rictor Rheb_P Rheb RSK_P RSK PDK1_P PDK1 MLL_P MLL HOXA9 GBL_P GBL ERK_P ERK AKT_P AKT**
		**miRNA_1976 miRNA_196B mTOR_P mTOR TSC_1_2 Rictor_P Rictor Rheb_P Rheb RSK_P RSK PDK1_P PDK1 MLL_P MLL HOXA9 GBL_P GBL ERK_P ERK AKT_P AKT**
		**miRNA_1976 miRNA_196B mTOR_P mTOR TSC_1_2 Rictor_P Rictor Rheb_P Rheb RSK_P RSK PDK1_P PDK1 MLL_P MLL HOXA9 GBL_P GBL ERK_P ERK AKT_P AKT**
		**miRNA_1976 miRNA_196B mTOR_P mTOR TSC_1_2 Rictor_P Rictor Rheb_P Rheb RSK_P RSK PDK1_P PDK1 MLL_P MLL HOXA9 GBL_P GBL ERK_P ERK AKT_P AKT**
**49**	**1352**	Basin Dimension Percentual: **1.06**
		**miRNA_1976 miRNA_196B mTOR_P mTOR TSC_1_2 Rictor_P Rictor Rheb_P Rheb RSK_P RSK PDK1_P PDK1 MLL_P MLL HOXA9 GBL_P GBL ERK_P ERK AKT_P AKT**
		**miRNA_1976 miRNA_196B mTOR_P mTOR TSC_1_2 Rictor_P Rictor Rheb_P Rheb RSK_P RSK PDK1_P PDK1 MLL_P MLL HOXA9 GBL_P GBL ERK_P ERK AKT_P AKT**
		**miRNA_1976 miRNA_196B mTOR_P mTOR TSC_1_2 Rictor_P Rictor Rheb_P Rheb RSK_P RSK PDK1_P PDK1 MLL_P MLL HOXA9 GBL_P GBL ERK_P ERK AKT_P AKT**
		**miRNA_1976 miRNA_196B mTOR_P mTOR TSC_1_2 Rictor_P Rictor Rheb_P Rheb RSK_P RSK PDK1_P PDK1 MLL_P MLL HOXA9 GBL_P GBL ERK_P ERK AKT_P AKT**
		**miRNA_1976 miRNA_196B mTOR_P mTOR TSC_1_2 Rictor_P Rictor Rheb_P Rheb RSK_P RSK PDK1_P PDK1 MLL_P MLL HOXA9 GBL_P GBL ERK_P ERK AKT_P AKT**
		**miRNA_1976 miRNA_196B mTOR_P mTOR TSC_1_2 Rictor_P Rictor Rheb_P Rheb RSK_P RSK PDK1_P PDK1 MLL_P MLL HOXA9 GBL_P GBL ERK_P ERK AKT_P AKT**
		**miRNA_1976 miRNA_196B mTOR_P mTOR TSC_1_2 Rictor_P Rictor Rheb_P Rheb RSK_P RSK PDK1_P PDK1 MLL_P MLL HOXA9 GBL_P GBL ERK_P ERK AKT_P AKT**
**74**	**1328**	Basin Dimension Percentual: **1.04**
		**miRNA_1976 miRNA_196B mTOR_P mTOR TSC_1_2 Rictor_P Rictor Rheb_P Rheb RSK_P RSK PDK1_P PDK1 MLL_P MLL HOXA9 GBL_P GBL ERK_P ERK AKT_P AKT**

		**miRNA_1976 miRNA_196B mTOR_P mTOR TSC_1_2 Rictor_P Rictor Rheb_P Rheb RSK_P RSK PDK1_P PDK1 MLL_P MLL HOXA9 GBL_P GBL ERK_P ERK AKT_P AKT**
		**miRNA_1976 miRNA_196B mTOR_P mTOR TSC_1_2 Rictor_P Rictor Rheb_P Rheb RSK_P RSK PDK1_P PDK1 MLL_P MLL HOXA9 GBL_P GBL ERK_P ERK AKT_P AKT**
		**miRNA_1976 miRNA_196B mTOR_P mTOR TSC_1_2 Rictor_P Rictor Rheb_P Rheb RSK_P RSK PDK1_P PDK1 MLL_P MLL HOXA9 GBL_P GBL ERK_P ERK AKT_P AKT**
		**miRNA_1976 miRNA_196B mTOR_P mTOR TSC_1_2 Rictor_P Rictor Rheb_P Rheb RSK_P RSK PDK1_P PDK1 MLL_P MLL HOXA9 GBL_P GBL ERK_P ERK AKT_P AKT**
		**miRNA_1976 miRNA_196B mTOR_P mTOR TSC_1_2 Rictor_P Rictor Rheb_P Rheb RSK_P RSK PDK1_P PDK1 MLL_P MLL HOXA9 GBL_P GBL ERK_P ERK AKT_P AKT**
		**miRNA_1976 miRNA_196B mTOR_P mTOR TSC_1_2 Rictor_P Rictor Rheb_P Rheb RSK_P RSK PDK1_P PDK1 MLL_P MLL HOXA9 GBL_P GBL ERK_P ERK AKT_P AKT**
**23**	**1296**	Basin Dimension Percentual: **1.01**
		**miRNA_1976 miRNA_196B mTOR_P mTOR TSC_1_2 Rictor_P Rictor Rheb_P Rheb RSK_P RSK PDK1_P PDK1 MLL_P MLL HOXA9 GBL_P GBL ERK_P ERK AKT_P AKT**
**63**	**1266**	Basin Dimension Percentual: **0.99**
		**miRNA_1976 miRNA_196B mTOR_P mTOR TSC_1_2 Rictor_P Rictor Rheb_P Rheb RSK_P RSK PDK1_P PDK1 MLL_P MLL HOXA9 GBL_P GBL ERK_P ERK AKT_P AKT**
		**miRNA_1976 miRNA_196B mTOR_P mTOR TSC_1_2 Rictor_P Rictor Rheb_P Rheb RSK_P RSK PDK1_P PDK1 MLL_P MLL HOXA9 GBL_P GBL ERK_P ERK AKT_P AKT**
		**miRNA_1976 miRNA_196B mTOR_P mTOR TSC_1_2 Rictor_P Rictor Rheb_P Rheb RSK_P RSK PDK1_P PDK1 MLL_P MLL HOXA9 GBL_P GBL ERK_P ERK AKT_P AKT**
		**miRNA_1976 miRNA_196B mTOR_P mTOR TSC_1_2 Rictor_P Rictor Rheb_P Rheb RSK_P RSK PDK1_P PDK1 MLL_P MLL HOXA9 GBL_P GBL ERK_P ERK AKT_P AKT**
		**miRNA_1976 miRNA_196B mTOR_P mTOR TSC_1_2 Rictor_P Rictor Rheb_P Rheb RSK_P RSK PDK1_P PDK1 MLL_P MLL HOXA9 GBL_P GBL ERK_P ERK AKT_P AKT**

		**miRNA_1976 miRNA_196B mTOR_P mTOR TSC_1_2 Rictor_P Rictor Rheb_P Rheb RSK_P RSK PDK1_P PDK1 MLL_P MLL HOXA9 GBL_P GBL ERK_P ERK AKT_P AKT**
		**miRNA_1976 miRNA_196B mTOR_P mTOR TSC_1_2 Rictor_P Rictor Rheb_P Rheb RSK_P RSK PDK1_P PDK1 MLL_P MLL HOXA9 GBL_P GBL ERK_P ERK AKT_P AKT**
**52**	**974**	Basin Dimension Percentual: **0.76**
		**miRNA_1976 miRNA_196B mTOR_P mTOR TSC_1_2 Rictor_P Rictor Rheb_P Rheb RSK_P RSK PDK1_P PDK1 MLL_P MLL HOXA9 GBL_P GBL ERK_P ERK AKT_P AKT**
		**miRNA_1976 miRNA_196B mTOR_P mTOR TSC_1_2 Rictor_P Rictor Rheb_P Rheb RSK_P RSK PDK1_P PDK1 MLL_P MLL HOXA9 GBL_P GBL ERK_P ERK AKT_P AKT**
		**miRNA_1976 miRNA_196B mTOR_P mTOR TSC_1_2 Rictor_P Rictor Rheb_P Rheb RSK_P RSK PDK1_P PDK1 MLL_P MLL HOXA9 GBL_P GBL ERK_P ERK AKT_P AKT**
		**miRNA_1976 miRNA_196B mTOR_P mTOR TSC_1_2 Rictor_P Rictor Rheb_P Rheb RSK_P RSK PDK1_P PDK1 MLL_P MLL HOXA9 GBL_P GBL ERK_P ERK AKT_P AKT**
		**miRNA_1976 miRNA_196B mTOR_P mTOR TSC_1_2 Rictor_P Rictor Rheb_P Rheb RSK_P RSK PDK1_P PDK1 MLL_P MLL HOXA9 GBL_P GBL ERK_P ERK AKT_P AKT**
		**miRNA_1976 miRNA_196B mTOR_P mTOR TSC_1_2 Rictor_P Rictor Rheb_P Rheb RSK_P RSK PDK1_P PDK1 MLL_P MLL HOXA9 GBL_P GBL ERK_P ERK AKT_P AKT**
		**miRNA_1976 miRNA_196B mTOR_P mTOR TSC_1_2 Rictor_P Rictor Rheb_P Rheb RSK_P RSK PDK1_P PDK1 MLL_P MLL HOXA9 GBL_P GBL ERK_P ERK AKT_P AKT**
**60**	**924**	Basin Dimension Percentual: **0.72**
		**miRNA_1976 miRNA_196B mTOR_P mTOR TSC_1_2 Rictor_P Rictor Rheb_P Rheb RSK_P RSK PDK1_P PDK1 MLL_P MLL HOXA9 GBL_P GBL ERK_P ERK AKT_P AKT**
		**miRNA_1976 miRNA_196B mTOR_P mTOR TSC_1_2 Rictor_P Rictor Rheb_P Rheb RSK_P RSK PDK1_P PDK1 MLL_P MLL HOXA9 GBL_P GBL ERK_P ERK AKT_P AKT**
		**miRNA_1976 miRNA_196B mTOR_P mTOR TSC_1_2 Rictor_P Rictor Rheb_P Rheb RSK_P RSK PDK1_P PDK1 MLL_P MLL HOXA9 GBL_P GBL ERK_P ERK AKT_P AKT**

		**miRNA_1976 miRNA_196B mTOR_P mTOR TSC_1_2 Rictor_P Rictor Rheb_P Rheb RSK_P RSK PDK1_P PDK1 MLL_P MLL HOXA9 GBL_P GBL ERK_P ERK AKT_P AKT**
		**miRNA_1976 miRNA_196B mTOR_P mTOR TSC_1_2 Rictor_P Rictor Rheb_P Rheb RSK_P RSK PDK1_P PDK1 MLL_P MLL HOXA9 GBL_P GBL ERK_P ERK AKT_P AKT**
		**miRNA_1976 miRNA_196B mTOR_P mTOR TSC_1_2 Rictor_P Rictor Rheb_P Rheb RSK_P RSK PDK1_P PDK1 MLL_P MLL HOXA9 GBL_P GBL ERK_P ERK AKT_P AKT**
		**miRNA_1976 miRNA_196B mTOR_P mTOR TSC_1_2 Rictor_P Rictor Rheb_P Rheb RSK_P RSK PDK1_P PDK1 MLL_P MLL HOXA9 GBL_P GBL ERK_P ERK AKT_P AKT**
**5**	**880**	Basin Dimension Percentual: **0.69**
		**miRNA_1976 miRNA_196B mTOR_P mTOR TSC_1_2 Rictor_P Rictor Rheb_P Rheb RSK_P RSK PDK1_P PDK1 MLL_P MLL HOXA9 GBL_P GBL ERK_P ERK AKT_P AKT**
		**miRNA_1976 miRNA_196B mTOR_P mTOR TSC_1_2 Rictor_P Rictor Rheb_P Rheb RSK_P RSK PDK1_P PDK1 MLL_P MLL HOXA9 GBL_P GBL ERK_P ERK AKT_P AKT**
		**miRNA_1976 miRNA_196B mTOR_P mTOR TSC_1_2 Rictor_P Rictor Rheb_P Rheb RSK_P RSK PDK1_P PDK1 MLL_P MLL HOXA9 GBL_P GBL ERK_P ERK AKT_P AKT**
		**miRNA_1976 miRNA_196B mTOR_P mTOR TSC_1_2 Rictor_P Rictor Rheb_P Rheb RSK_P RSK PDK1_P PDK1 MLL_P MLL HOXA9 GBL_P GBL ERK_P ERK AKT_P AKT**
		**miRNA_1976 miRNA_196B mTOR_P mTOR TSC_1_2 Rictor_P Rictor Rheb_P Rheb RSK_P RSK PDK1_P PDK1 MLL_P MLL HOXA9 GBL_P GBL ERK_P ERK AKT_P AKT**
		**miRNA_1976 miRNA_196B mTOR_P mTOR TSC_1_2 Rictor_P Rictor Rheb_P Rheb RSK_P RSK PDK1_P PDK1 MLL_P MLL HOXA9 GBL_P GBL ERK_P ERK AKT_P AKT**
		**miRNA_1976 miRNA_196B mTOR_P mTOR TSC_1_2 Rictor_P Rictor Rheb_P Rheb RSK_P RSK PDK1_P PDK1 MLL_P MLL HOXA9 GBL_P GBL ERK_P ERK AKT_P AKT**
**55**	**876**	Basin Dimension Percentual: **0.69**
		**miRNA_1976 miRNA_196B mTOR_P mTOR TSC_1_2 Rictor_P Rictor Rheb_P Rheb RSK_P RSK PDK1_P PDK1 MLL_P MLL HOXA9 GBL_P GBL ERK_P ERK AKT_P AKT**

		**miRNA_1976 miRNA_196B mTOR_P mTOR TSC_1_2 Rictor_P Rictor Rheb_P Rheb RSK_P RSK PDK1_P PDK1 MLL_P MLL HOXA9 GBL_P GBL ERK_P ERK AKT_P AKT**
		**miRNA_1976 miRNA_196B mTOR_P mTOR TSC_1_2 Rictor_P Rictor Rheb_P Rheb RSK_P RSK PDK1_P PDK1 MLL_P MLL HOXA9 GBL_P GBL ERK_P ERK AKT_P AKT**
		**miRNA_1976 miRNA_196B mTOR_P mTOR TSC_1_2 Rictor_P Rictor Rheb_P Rheb RSK_P RSK PDK1_P PDK1 MLL_P MLL HOXA9 GBL_P GBL ERK_P ERK AKT_P AKT**
		**miRNA_1976 miRNA_196B mTOR_P mTOR TSC_1_2 Rictor_P Rictor Rheb_P Rheb RSK_P RSK PDK1_P PDK1 MLL_P MLL HOXA9 GBL_P GBL ERK_P ERK AKT_P AKT**
		**miRNA_1976 miRNA_196B mTOR_P mTOR TSC_1_2 Rictor_P Rictor Rheb_P Rheb RSK_P RSK PDK1_P PDK1 MLL_P MLL HOXA9 GBL_P GBL ERK_P ERK AKT_P AKT**
		**miRNA_1976 miRNA_196B mTOR_P mTOR TSC_1_2 Rictor_P Rictor Rheb_P Rheb RSK_P RSK PDK1_P PDK1 MLL_P MLL HOXA9 GBL_P GBL ERK_P ERK AKT_P AKT**
**57**	**870**	Basin Dimension Percentual: **0.68**
		**miRNA_1976 miRNA_196B mTOR_P mTOR TSC_1_2 Rictor_P Rictor Rheb_P Rheb RSK_P RSK PDK1_P PDK1 MLL_P MLL HOXA9 GBL_P GBL ERK_P ERK AKT_P AKT**
		**miRNA_1976 miRNA_196B mTOR_P mTOR TSC_1_2 Rictor_P Rictor Rheb_P Rheb RSK_P RSK PDK1_P PDK1 MLL_P MLL HOXA9 GBL_P GBL ERK_P ERK AKT_P AKT**
		**miRNA_1976 miRNA_196B mTOR_P mTOR TSC_1_2 Rictor_P Rictor Rheb_P Rheb RSK_P RSK PDK1_P PDK1 MLL_P MLL HOXA9 GBL_P GBL ERK_P ERK AKT_P AKT**
		**miRNA_1976 miRNA_196B mTOR_P mTOR TSC_1_2 Rictor_P Rictor Rheb_P Rheb RSK_P RSK PDK1_P PDK1 MLL_P MLL HOXA9 GBL_P GBL ERK_P ERK AKT_P AKT**
		**miRNA_1976 miRNA_196B mTOR_P mTOR TSC_1_2 Rictor_P Rictor Rheb_P Rheb RSK_P RSK PDK1_P PDK1 MLL_P MLL HOXA9 GBL_P GBL ERK_P ERK AKT_P AKT**
		**miRNA_1976 miRNA_196B mTOR_P mTOR TSC_1_2 Rictor_P Rictor Rheb_P Rheb RSK_P RSK PDK1_P PDK1 MLL_P MLL HOXA9 GBL_P GBL ERK_P ERK AKT_P AKT**
		**miRNA_1976 miRNA_196B mTOR_P mTOR TSC_1_2 Rictor_P Rictor Rheb_P Rheb RSK_P RSK PDK1_P PDK1 MLL_P MLL**

		**HOXA9 GBL_P GBL ERK_P ERK AKT_P AKT**
**73**	**864**	Basin Dimension Percentual: **0.68**
		**miRNA_1976 miRNA_196B mTOR_P mTOR TSC_1_2 Rictor_P Rictor Rheb_P Rheb RSK_P RSK PDK1_P PDK1 MLL_P MLL HOXA9 GBL_P GBL ERK_P ERK AKT_P AKT**
		**miRNA_1976 miRNA_196B mTOR_P mTOR TSC_1_2 Rictor_P Rictor Rheb_P Rheb RSK_P RSK PDK1_P PDK1 MLL_P MLL HOXA9 GBL_P GBL ERK_P ERK AKT_P AKT**
		**miRNA_1976 miRNA_196B mTOR_P mTOR TSC_1_2 Rictor_P Rictor Rheb_P Rheb RSK_P RSK PDK1_P PDK1 MLL_P MLL HOXA9 GBL_P GBL ERK_P ERK AKT_P AKT**
		**miRNA_1976 miRNA_196B mTOR_P mTOR TSC_1_2 Rictor_P Rictor Rheb_P Rheb RSK_P RSK PDK1_P PDK1 MLL_P MLL HOXA9 GBL_P GBL ERK_P ERK AKT_P AKT**
		**miRNA_1976 miRNA_196B mTOR_P mTOR TSC_1_2 Rictor_P Rictor Rheb_P Rheb RSK_P RSK PDK1_P PDK1 MLL_P MLL HOXA9 GBL_P GBL ERK_P ERK AKT_P AKT**
		**miRNA_1976 miRNA_196B mTOR_P mTOR TSC_1_2 Rictor_P Rictor Rheb_P Rheb RSK_P RSK PDK1_P PDK1 MLL_P MLL HOXA9 GBL_P GBL ERK_P ERK AKT_P AKT**
		**miRNA_1976 miRNA_196B mTOR_P mTOR TSC_1_2 Rictor_P Rictor Rheb_P Rheb RSK_P RSK PDK1_P PDK1 MLL_P MLL HOXA9 GBL_P GBL ERK_P ERK AKT_P AKT**
**56**	**816**	Basin Dimension Percentual: **0.64**
		**miRNA_1976 miRNA_196B mTOR_P mTOR TSC_1_2 Rictor_P Rictor Rheb_P Rheb RSK_P RSK PDK1_P PDK1 MLL_P MLL HOXA9 GBL_P GBL ERK_P ERK AKT_P AKT**
		**miRNA_1976 miRNA_196B mTOR_P mTOR TSC_1_2 Rictor_P Rictor Rheb_P Rheb RSK_P RSK PDK1_P PDK1 MLL_P MLL HOXA9 GBL_P GBL ERK_P ERK AKT_P AKT**
		**miRNA_1976 miRNA_196B mTOR_P mTOR TSC_1_2 Rictor_P Rictor Rheb_P Rheb RSK_P RSK PDK1_P PDK1 MLL_P MLL HOXA9 GBL_P GBL ERK_P ERK AKT_P AKT**
		**miRNA_1976 miRNA_196B mTOR_P mTOR TSC_1_2 Rictor_P Rictor Rheb_P Rheb RSK_P RSK PDK1_P PDK1 MLL_P MLL HOXA9 GBL_P GBL ERK_P ERK AKT_P AKT**
		**miRNA_1976 miRNA_196B mTOR_P mTOR TSC_1_2 Rictor_P Rictor Rheb_P Rheb RSK_P RSK PDK1_P PDK1 MLL_P MLL HOXA9 GBL_P GBL ERK_P ERK AKT_P AKT**

		**miRNA_1976 miRNA_196B mTOR_P mTOR TSC_1_2 Rictor_P Rictor Rheb_P Rheb RSK_P RSK PDK1_P PDK1 MLL_P MLL HOXA9 GBL_P GBL ERK_P ERK AKT_P AKT**
		**miRNA_1976 miRNA_196B mTOR_P mTOR TSC_1_2 Rictor_P Rictor Rheb_P Rheb RSK_P RSK PDK1_P PDK1 MLL_P MLL HOXA9 GBL_P GBL ERK_P ERK AKT_P AKT**
**48**	**748**	Basin Dimension Percentual: **0.59**
		**miRNA_1976 miRNA_196B mTOR_P mTOR TSC_1_2 Rictor_P Rictor Rheb_P Rheb RSK_P RSK PDK1_P PDK1 MLL_P MLL HOXA9 GBL_P GBL ERK_P ERK AKT_P AKT**
		**miRNA_1976 miRNA_196B mTOR_P mTOR TSC_1_2 Rictor_P Rictor Rheb_P Rheb RSK_P RSK PDK1_P PDK1 MLL_P MLL HOXA9 GBL_P GBL ERK_P ERK AKT_P AKT**
		**miRNA_1976 miRNA_196B mTOR_P mTOR TSC_1_2 Rictor_P Rictor Rheb_P Rheb RSK_P RSK PDK1_P PDK1 MLL_P MLL HOXA9 GBL_P GBL ERK_P ERK AKT_P AKT**
		**miRNA_1976 miRNA_196B mTOR_P mTOR TSC_1_2 Rictor_P Rictor Rheb_P Rheb RSK_P RSK PDK1_P PDK1 MLL_P MLL HOXA9 GBL_P GBL ERK_P ERK AKT_P AKT**
		**miRNA_1976 miRNA_196B mTOR_P mTOR TSC_1_2 Rictor_P Rictor Rheb_P Rheb RSK_P RSK PDK1_P PDK1 MLL_P MLL HOXA9 GBL_P GBL ERK_P ERK AKT_P AKT**
		**miRNA_1976 miRNA_196B mTOR_P mTOR TSC_1_2 Rictor_P Rictor Rheb_P Rheb RSK_P RSK PDK1_P PDK1 MLL_P MLL HOXA9 GBL_P GBL ERK_P ERK AKT_P AKT**
		**miRNA_1976 miRNA_196B mTOR_P mTOR TSC_1_2 Rictor_P Rictor Rheb_P Rheb RSK_P RSK PDK1_P PDK1 MLL_P MLL HOXA9 GBL_P GBL ERK_P ERK AKT_P AKT**
**7**	**676**	Basin Dimension Percentual: **0.53**
		**miRNA_1976 miRNA_196B mTOR_P mTOR TSC_1_2 Rictor_P Rictor Rheb_P Rheb RSK_P RSK PDK1_P PDK1 MLL_P MLL HOXA9 GBL_P GBL ERK_P ERK AKT_P AKT**
		**miRNA_1976 miRNA_196B mTOR_P mTOR TSC_1_2 Rictor_P Rictor Rheb_P Rheb RSK_P RSK PDK1_P PDK1 MLL_P MLL HOXA9 GBL_P GBL ERK_P ERK AKT_P AKT**
		**miRNA_1976 miRNA_196B mTOR_P mTOR TSC_1_2 Rictor_P Rictor Rheb_P Rheb RSK_P RSK PDK1_P PDK1 MLL_P MLL HOXA9 GBL_P GBL ERK_P ERK AKT_P AKT**

		**miRNA_1976 miRNA_196B mTOR_P mTOR TSC_1_2 Rictor_P Rictor Rheb_P Rheb RSK_P RSK PDK1_P PDK1 MLL_P MLL HOXA9 GBL_P GBL ERK_P ERK AKT_P AKT**
		**miRNA_1976 miRNA_196B mTOR_P mTOR TSC_1_2 Rictor_P Rictor Rheb_P Rheb RSK_P RSK PDK1_P PDK1 MLL_P MLL HOXA9 GBL_P GBL ERK_P ERK AKT_P AKT**
		**miRNA_1976 miRNA_196B mTOR_P mTOR TSC_1_2 Rictor_P Rictor Rheb_P Rheb RSK_P RSK PDK1_P PDK1 MLL_P MLL HOXA9 GBL_P GBL ERK_P ERK AKT_P AKT**
		**miRNA_1976 miRNA_196B mTOR_P mTOR TSC_1_2 Rictor_P Rictor Rheb_P Rheb RSK_P RSK PDK1_P PDK1 MLL_P MLL HOXA9 GBL_P GBL ERK_P ERK AKT_P AKT**
**50**	**668**	Basin Dimension Percentual: **0.52**
		**miRNA_1976 miRNA_196B mTOR_P mTOR TSC_1_2 Rictor_P Rictor Rheb_P Rheb RSK_P RSK PDK1_P PDK1 MLL_P MLL HOXA9 GBL_P GBL ERK_P ERK AKT_P AKT**
		**miRNA_1976 miRNA_196B mTOR_P mTOR TSC_1_2 Rictor_P Rictor Rheb_P Rheb RSK_P RSK PDK1_P PDK1 MLL_P MLL HOXA9 GBL_P GBL ERK_P ERK AKT_P AKT**
		**miRNA_1976 miRNA_196B mTOR_P mTOR TSC_1_2 Rictor_P Rictor Rheb_P Rheb RSK_P RSK PDK1_P PDK1 MLL_P MLL HOXA9 GBL_P GBL ERK_P ERK AKT_P AKT**
		**miRNA_1976 miRNA_196B mTOR_P mTOR TSC_1_2 Rictor_P Rictor Rheb_P Rheb RSK_P RSK PDK1_P PDK1 MLL_P MLL HOXA9 GBL_P GBL ERK_P ERK AKT_P AKT**
		**miRNA_1976 miRNA_196B mTOR_P mTOR TSC_1_2 Rictor_P Rictor Rheb_P Rheb RSK_P RSK PDK1_P PDK1 MLL_P MLL HOXA9 GBL_P GBL ERK_P ERK AKT_P AKT**
		**miRNA_1976 miRNA_196B mTOR_P mTOR TSC_1_2 Rictor_P Rictor Rheb_P Rheb RSK_P RSK PDK1_P PDK1 MLL_P MLL HOXA9 GBL_P GBL ERK_P ERK AKT_P AKT**
		**miRNA_1976 miRNA_196B mTOR_P mTOR TSC_1_2 Rictor_P Rictor Rheb_P Rheb RSK_P RSK PDK1_P PDK1 MLL_P MLL HOXA9 GBL_P GBL ERK_P ERK AKT_P AKT**
**17**	**462**	Basin Dimension Percentual: **0.36**
		**miRNA_1976 miRNA_196B mTOR_P mTOR TSC_1_2 Rictor_P Rictor Rheb_P Rheb RSK_P RSK PDK1_P PDK1 MLL_P MLL HOXA9 GBL_P GBL ERK_P ERK AKT_P AKT**

		**miRNA_1976 miRNA_196B mTOR_P mTOR TSC_1_2 Rictor_P Rictor Rheb_P Rheb RSK_P RSK PDK1_P PDK1 MLL_P MLL HOXA9 GBL_P GBL ERK_P ERK AKT_P AKT**
		**miRNA_1976 miRNA_196B mTOR_P mTOR TSC_1_2 Rictor_P Rictor Rheb_P Rheb RSK_P RSK PDK1_P PDK1 MLL_P MLL HOXA9 GBL_P GBL ERK_P ERK AKT_P AKT**
		**miRNA_1976 miRNA_196B mTOR_P mTOR TSC_1_2 Rictor_P Rictor Rheb_P Rheb RSK_P RSK PDK1_P PDK1 MLL_P MLL HOXA9 GBL_P GBL ERK_P ERK AKT_P AKT**
		**miRNA_1976 miRNA_196B mTOR_P mTOR TSC_1_2 Rictor_P Rictor Rheb_P Rheb RSK_P RSK PDK1_P PDK1 MLL_P MLL HOXA9 GBL_P GBL ERK_P ERK AKT_P AKT**
		**miRNA_1976 miRNA_196B mTOR_P mTOR TSC_1_2 Rictor_P Rictor Rheb_P Rheb RSK_P RSK PDK1_P PDK1 MLL_P MLL HOXA9 GBL_P GBL ERK_P ERK AKT_P AKT**
		**miRNA_1976 miRNA_196B mTOR_P mTOR TSC_1_2 Rictor_P Rictor Rheb_P Rheb RSK_P RSK PDK1_P PDK1 MLL_P MLL HOXA9 GBL_P GBL ERK_P ERK AKT_P AKT**
**71**	**448**	Basin Dimension Percentual: **0.35**
		**miRNA_1976 miRNA_196B mTOR_P mTOR TSC_1_2 Rictor_P Rictor Rheb_P Rheb RSK_P RSK PDK1_P PDK1 MLL_P MLL HOXA9 GBL_P GBL ERK_P ERK AKT_P AKT**
**58**	**444**	Basin Dimension Percentual: **0.35**
		**miRNA_1976 miRNA_196B mTOR_P mTOR TSC_1_2 Rictor_P Rictor Rheb_P Rheb RSK_P RSK PDK1_P PDK1 MLL_P MLL HOXA9 GBL_P GBL ERK_P ERK AKT_P AKT**
		**miRNA_1976 miRNA_196B mTOR_P mTOR TSC_1_2 Rictor_P Rictor Rheb_P Rheb RSK_P RSK PDK1_P PDK1 MLL_P MLL HOXA9 GBL_P GBL ERK_P ERK AKT_P AKT**
		**miRNA_1976 miRNA_196B mTOR_P mTOR TSC_1_2 Rictor_P Rictor Rheb_P Rheb RSK_P RSK PDK1_P PDK1 MLL_P MLL HOXA9 GBL_P GBL ERK_P ERK AKT_P AKT**
		**miRNA_1976 miRNA_196B mTOR_P mTOR TSC_1_2 Rictor_P Rictor Rheb_P Rheb RSK_P RSK PDK1_P PDK1 MLL_P MLL HOXA9 GBL_P GBL ERK_P ERK AKT_P AKT**
		**miRNA_1976 miRNA_196B mTOR_P mTOR TSC_1_2 Rictor_P Rictor Rheb_P Rheb RSK_P RSK PDK1_P PDK1 MLL_P MLL HOXA9 GBL_P GBL ERK_P ERK AKT_P AKT**
		**miRNA_1976 miRNA_196B mTOR_P mTOR TSC_1_2 Rictor_P**

		**Rictor Rheb_P Rheb RSK_P RSK PDK1_P PDK1 MLL_P MLL**<br>**HOXA9 GBL_P GBL ERK_P ERK AKT_P AKT**
		**miRNA_1976 miRNA_196B mTOR_P mTOR TSC_1_2 Rictor_P**<br>**Rictor Rheb_P Rheb RSK_P RSK PDK1_P PDK1 MLL_P MLL**<br>**HOXA9 GBL_P GBL ERK_P ERK AKT_P AKT**
**1**	**440**	Basin Dimension Percentual: **0.34**
		**miRNA_1976 miRNA_196B mTOR_P mTOR TSC_1_2 Rictor_P**<br>**Rictor Rheb_P Rheb RSK_P RSK PDK1_P PDK1 MLL_P MLL**<br>**HOXA9 GBL_P GBL ERK_P ERK AKT_P AKT**
		**miRNA_1976 miRNA_196B mTOR_P mTOR TSC_1_2 Rictor_P**<br>**Rictor Rheb_P Rheb RSK_P RSK PDK1_P PDK1 MLL_P MLL**<br>**HOXA9 GBL_P GBL ERK_P ERK AKT_P AKT**
		**miRNA_1976 miRNA_196B mTOR_P mTOR TSC_1_2 Rictor_P**<br>**Rictor Rheb_P Rheb RSK_P RSK PDK1_P PDK1 MLL_P MLL**<br>**HOXA9 GBL_P GBL ERK_P ERK AKT_P AKT**
		**miRNA_1976 miRNA_196B mTOR_P mTOR TSC_1_2 Rictor_P**<br>**Rictor Rheb_P Rheb RSK_P RSK PDK1_P PDK1 MLL_P MLL**<br>**HOXA9 GBL_P GBL ERK_P ERK AKT_P AKT**
		**miRNA_1976 miRNA_196B mTOR_P mTOR TSC_1_2 Rictor_P**<br>**Rictor Rheb_P Rheb RSK_P RSK PDK1_P PDK1 MLL_P MLL**<br>**HOXA9 GBL_P GBL ERK_P ERK AKT_P AKT**
		**miRNA_1976 miRNA_196B mTOR_P mTOR TSC_1_2 Rictor_P**<br>**Rictor Rheb_P Rheb RSK_P RSK PDK1_P PDK1 MLL_P MLL**<br>**HOXA9 GBL_P GBL ERK_P ERK AKT_P AKT**
		**miRNA_1976 miRNA_196B mTOR_P mTOR TSC_1_2 Rictor_P**<br>**Rictor Rheb_P Rheb RSK_P RSK PDK1_P PDK1 MLL_P MLL**<br>**HOXA9 GBL_P GBL ERK_P ERK AKT_P AKT**
**11**	**438**	Basin Dimension Percentual: **0.34**
		**miRNA_1976 miRNA_196B mTOR_P mTOR TSC_1_2 Rictor_P**<br>**Rictor Rheb_P Rheb RSK_P RSK PDK1_P PDK1 MLL_P MLL**<br>**HOXA9 GBL_P GBL ERK_P ERK AKT_P AKT**
		**miRNA_1976 miRNA_196B mTOR_P mTOR TSC_1_2 Rictor_P**<br>**Rictor Rheb_P Rheb RSK_P RSK PDK1_P PDK1 MLL_P MLL**<br>**HOXA9 GBL_P GBL ERK_P ERK AKT_P AKT**
		**miRNA_1976 miRNA_196B mTOR_P mTOR TSC_1_2 Rictor_P**<br>**Rictor Rheb_P Rheb RSK_P RSK PDK1_P PDK1 MLL_P MLL**<br>**HOXA9 GBL_P GBL ERK_P ERK AKT_P AKT**
		**miRNA_1976 miRNA_196B mTOR_P mTOR TSC_1_2 Rictor_P**<br>**Rictor Rheb_P Rheb RSK_P RSK PDK1_P PDK1 MLL_P MLL**

		**HOXA9 GBL_P GBL ERK_P ERK AKT_P AKT**
		**miRNA_1976 miRNA_196B mTOR_P mTOR TSC_1_2 Rictor_P Rictor Rheb_P Rheb RSK_P RSK PDK1_P PDK1 MLL_P MLL HOXA9 GBL_P GBL ERK_P ERK AKT_P AKT**
		**miRNA_1976 miRNA_196B mTOR_P mTOR TSC_1_2 Rictor_P Rictor Rheb_P Rheb RSK_P RSK PDK1_P PDK1 MLL_P MLL HOXA9 GBL_P GBL ERK_P ERK AKT_P AKT**
		**miRNA_1976 miRNA_196B mTOR_P mTOR TSC_1_2 Rictor_P Rictor Rheb_P Rheb RSK_P RSK PDK1_P PDK1 MLL_P MLL HOXA9 GBL_P GBL ERK_P ERK AKT_P AKT**
**28**	**432**	Basin Dimension Percentual: **0.34**
		**miRNA_1976 miRNA_196B mTOR_P mTOR TSC_1_2 Rictor_P Rictor Rheb_P Rheb RSK_P RSK PDK1_P PDK1 MLL_P MLL HOXA9 GBL_P GBL ERK_P ERK AKT_P AKT**
		**miRNA_1976 miRNA_196B mTOR_P mTOR TSC_1_2 Rictor_P Rictor Rheb_P Rheb RSK_P RSK PDK1_P PDK1 MLL_P MLL HOXA9 GBL_P GBL ERK_P ERK AKT_P AKT**
		**miRNA_1976 miRNA_196B mTOR_P mTOR TSC_1_2 Rictor_P Rictor Rheb_P Rheb RSK_P RSK PDK1_P PDK1 MLL_P MLL HOXA9 GBL_P GBL ERK_P ERK AKT_P AKT**
		**miRNA_1976 miRNA_196B mTOR_P mTOR TSC_1_2 Rictor_P Rictor Rheb_P Rheb RSK_P RSK PDK1_P PDK1 MLL_P MLL HOXA9 GBL_P GBL ERK_P ERK AKT_P AKT**
		**miRNA_1976 miRNA_196B mTOR_P mTOR TSC_1_2 Rictor_P Rictor Rheb_P Rheb RSK_P RSK PDK1_P PDK1 MLL_P MLL HOXA9 GBL_P GBL ERK_P ERK AKT_P AKT**
		**miRNA_1976 miRNA_196B mTOR_P mTOR TSC_1_2 Rictor_P Rictor Rheb_P Rheb RSK_P RSK PDK1_P PDK1 MLL_P MLL HOXA9 GBL_P GBL ERK_P ERK AKT_P AKT**
		**miRNA_1976 miRNA_196B mTOR_P mTOR TSC_1_2 Rictor_P Rictor Rheb_P Rheb RSK_P RSK PDK1_P PDK1 MLL_P MLL HOXA9 GBL_P GBL ERK_P ERK AKT_P AKT**
**54**	**426**	Basin Dimension Percentual: **0.33**
		**miRNA_1976 miRNA_196B mTOR_P mTOR TSC_1_2 Rictor_P Rictor Rheb_P Rheb RSK_P RSK PDK1_P PDK1 MLL_P MLL HOXA9 GBL_P GBL ERK_P ERK AKT_P AKT**
		**miRNA_1976 miRNA_196B mTOR_P mTOR TSC_1_2 Rictor_P Rictor Rheb_P Rheb RSK_P RSK PDK1_P PDK1 MLL_P MLL**

		**HOXA9 GBL_P GBL ERK_P ERK AKT_P AKT**
		**miRNA_1976 miRNA_196B mTOR_P mTOR TSC_1_2 Rictor_P Rictor Rheb_P Rheb RSK_P RSK PDK1_P PDK1 MLL_P MLL HOXA9 GBL_P GBL ERK_P ERK AKT_P AKT**
		**miRNA_1976 miRNA_196B mTOR_P mTOR TSC_1_2 Rictor_P Rictor Rheb_P Rheb RSK_P RSK PDK1_P PDK1 MLL_P MLL HOXA9 GBL_P GBL ERK_P ERK AKT_P AKT**
		**miRNA_1976 miRNA_196B mTOR_P mTOR TSC_1_2 Rictor_P Rictor Rheb_P Rheb RSK_P RSK PDK1_P PDK1 MLL_P MLL HOXA9 GBL_P GBL ERK_P ERK AKT_P AKT**
		**miRNA_1976 miRNA_196B mTOR_P mTOR TSC_1_2 Rictor_P Rictor Rheb_P Rheb RSK_P RSK PDK1_P PDK1 MLL_P MLL HOXA9 GBL_P GBL ERK_P ERK AKT_P AKT**
		**miRNA_1976 miRNA_196B mTOR_P mTOR TSC_1_2 Rictor_P Rictor Rheb_P Rheb RSK_P RSK PDK1_P PDK1 MLL_P MLL HOXA9 GBL_P GBL ERK_P ERK AKT_P AKT**
**12**	**408**	Basin Dimension Percentual: **0.32**
		**miRNA_1976 miRNA_196B mTOR_P mTOR TSC_1_2 Rictor_P Rictor Rheb_P Rheb RSK_P RSK PDK1_P PDK1 MLL_P MLL HOXA9 GBL_P GBL ERK_P ERK AKT_P AKT**
		**miRNA_1976 miRNA_196B mTOR_P mTOR TSC_1_2 Rictor_P Rictor Rheb_P Rheb RSK_P RSK PDK1_P PDK1 MLL_P MLL HOXA9 GBL_P GBL ERK_P ERK AKT_P AKT**
		**miRNA_1976 miRNA_196B mTOR_P mTOR TSC_1_2 Rictor_P Rictor Rheb_P Rheb RSK_P RSK PDK1_P PDK1 MLL_P MLL HOXA9 GBL_P GBL ERK_P ERK AKT_P AKT**
		**miRNA_1976 miRNA_196B mTOR_P mTOR TSC_1_2 Rictor_P Rictor Rheb_P Rheb RSK_P RSK PDK1_P PDK1 MLL_P MLL HOXA9 GBL_P GBL ERK_P ERK AKT_P AKT**
		**miRNA_1976 miRNA_196B mTOR_P mTOR TSC_1_2 Rictor_P Rictor Rheb_P Rheb RSK_P RSK PDK1_P PDK1 MLL_P MLL HOXA9 GBL_P GBL ERK_P ERK AKT_P AKT**
		**miRNA_1976 miRNA_196B mTOR_P mTOR TSC_1_2 Rictor_P Rictor Rheb_P Rheb RSK_P RSK PDK1_P PDK1 MLL_P MLL HOXA9 GBL_P GBL ERK_P ERK AKT_P AKT**
		**miRNA_1976 miRNA_196B mTOR_P mTOR TSC_1_2 Rictor_P Rictor Rheb_P Rheb RSK_P RSK PDK1_P PDK1 MLL_P MLL HOXA9 GBL_P GBL ERK_P ERK AKT_P AKT**

**76**	**384**	Basin Dimension Percentual: **0.30**
		**miRNA_1976 miRNA_196B mTOR_P mTOR TSC_1_2 Rictor_P Rictor Rheb_P Rheb RSK_P RSK PDK1_P PDK1 MLL_P MLL HOXA9 GBL_P GBL ERK_P ERK AKT_P AKT**
		**miRNA_1976 miRNA_196B mTOR_P mTOR TSC_1_2 Rictor_P Rictor Rheb_P Rheb RSK_P RSK PDK1_P PDK1 MLL_P MLL HOXA9 GBL_P GBL ERK_P ERK AKT_P AKT**
		**miRNA_1976 miRNA_196B mTOR_P mTOR TSC_1_2 Rictor_P Rictor Rheb_P Rheb RSK_P RSK PDK1_P PDK1 MLL_P MLL HOXA9 GBL_P GBL ERK_P ERK AKT_P AKT**
		**miRNA_1976 miRNA_196B mTOR_P mTOR TSC_1_2 Rictor_P Rictor Rheb_P Rheb RSK_P RSK PDK1_P PDK1 MLL_P MLL HOXA9 GBL_P GBL ERK_P ERK AKT_P AKT**
		**miRNA_1976 miRNA_196B mTOR_P mTOR TSC_1_2 Rictor_P Rictor Rheb_P Rheb RSK_P RSK PDK1_P PDK1 MLL_P MLL HOXA9 GBL_P GBL ERK_P ERK AKT_P AKT**
		**miRNA_1976 miRNA_196B mTOR_P mTOR TSC_1_2 Rictor_P Rictor Rheb_P Rheb RSK_P RSK PDK1_P PDK1 MLL_P MLL HOXA9 GBL_P GBL ERK_P ERK AKT_P AKT**
		**miRNA_1976 miRNA_196B mTOR_P mTOR TSC_1_2 Rictor_P Rictor Rheb_P Rheb RSK_P RSK PDK1_P PDK1 MLL_P MLL HOXA9 GBL_P GBL ERK_P ERK AKT_P AKT**
**6**	**374**	Basin Dimension Percentual: **0.29**
		**miRNA_1976 miRNA_196B mTOR_P mTOR TSC_1_2 Rictor_P Rictor Rheb_P Rheb RSK_P RSK PDK1_P PDK1 MLL_P MLL HOXA9 GBL_P GBL ERK_P ERK AKT_P AKT**
		**miRNA_1976 miRNA_196B mTOR_P mTOR TSC_1_2 Rictor_P Rictor Rheb_P Rheb RSK_P RSK PDK1_P PDK1 MLL_P MLL HOXA9 GBL_P GBL ERK_P ERK AKT_P AKT**
		**miRNA_1976 miRNA_196B mTOR_P mTOR TSC_1_2 Rictor_P Rictor Rheb_P Rheb RSK_P RSK PDK1_P PDK1 MLL_P MLL HOXA9 GBL_P GBL ERK_P ERK AKT_P AKT**
		**miRNA_1976 miRNA_196B mTOR_P mTOR TSC_1_2 Rictor_P Rictor Rheb_P Rheb RSK_P RSK PDK1_P PDK1 MLL_P MLL HOXA9 GBL_P GBL ERK_P ERK AKT_P AKT**
		**miRNA_1976 miRNA_196B mTOR_P mTOR TSC_1_2 Rictor_P Rictor Rheb_P Rheb RSK_P RSK PDK1_P PDK1 MLL_P MLL HOXA9 GBL_P GBL ERK_P ERK AKT_P AKT**

		**miRNA_1976 miRNA_196B mTOR_P mTOR TSC_1_2 Rictor_P Rictor Rheb_P Rheb RSK_P RSK PDK1_P PDK1 MLL_P MLL HOXA9 GBL_P GBL ERK_P ERK AKT_P AKT**
		**miRNA_1976 miRNA_196B mTOR_P mTOR TSC_1_2 Rictor_P Rictor Rheb_P Rheb RSK_P RSK PDK1_P PDK1 MLL_P MLL HOXA9 GBL_P GBL ERK_P ERK AKT_P AKT**
**79**	**360**	Basin Dimension Percentual: **0.28**
		**miRNA_1976 miRNA_196B mTOR_P mTOR TSC_1_2 Rictor_P Rictor Rheb_P Rheb RSK_P RSK PDK1_P PDK1 MLL_P MLL HOXA9 GBL_P GBL ERK_P ERK AKT_P AKT**
		**miRNA_1976 miRNA_196B mTOR_P mTOR TSC_1_2 Rictor_P Rictor Rheb_P Rheb RSK_P RSK PDK1_P PDK1 MLL_P MLL HOXA9 GBL_P GBL ERK_P ERK AKT_P AKT**
		**miRNA_1976 miRNA_196B mTOR_P mTOR TSC_1_2 Rictor_P Rictor Rheb_P Rheb RSK_P RSK PDK1_P PDK1 MLL_P MLL HOXA9 GBL_P GBL ERK_P ERK AKT_P AKT**
		**miRNA_1976 miRNA_196B mTOR_P mTOR TSC_1_2 Rictor_P Rictor Rheb_P Rheb RSK_P RSK PDK1_P PDK1 MLL_P MLL HOXA9 GBL_P GBL ERK_P ERK AKT_P AKT**
		**miRNA_1976 miRNA_196B mTOR_P mTOR TSC_1_2 Rictor_P Rictor Rheb_P Rheb RSK_P RSK PDK1_P PDK1 MLL_P MLL HOXA9 GBL_P GBL ERK_P ERK AKT_P AKT**
		**miRNA_1976 miRNA_196B mTOR_P mTOR TSC_1_2 Rictor_P Rictor Rheb_P Rheb RSK_P RSK PDK1_P PDK1 MLL_P MLL HOXA9 GBL_P GBL ERK_P ERK AKT_P AKT**
		**miRNA_1976 miRNA_196B mTOR_P mTOR TSC_1_2 Rictor_P Rictor Rheb_P Rheb RSK_P RSK PDK1_P PDK1 MLL_P MLL HOXA9 GBL_P GBL ERK_P ERK AKT_P AKT**
**81**	**360**	Basin Dimension Percentual: **0.28**
		**miRNA_1976 miRNA_196B mTOR_P mTOR TSC_1_2 Rictor_P Rictor Rheb_P Rheb RSK_P RSK PDK1_P PDK1 MLL_P MLL HOXA9 GBL_P GBL ERK_P ERK AKT_P AKT**
		**miRNA_1976 miRNA_196B mTOR_P mTOR TSC_1_2 Rictor_P Rictor Rheb_P Rheb RSK_P RSK PDK1_P PDK1 MLL_P MLL HOXA9 GBL_P GBL ERK_P ERK AKT_P AKT**
		**miRNA_1976 miRNA_196B mTOR_P mTOR TSC_1_2 Rictor_P Rictor Rheb_P Rheb RSK_P RSK PDK1_P PDK1 MLL_P MLL HOXA9 GBL_P GBL ERK_P ERK AKT_P AKT**

		**miRNA_1976 miRNA_196B mTOR_P mTOR TSC_1_2 Rictor_P Rictor Rheb_P Rheb RSK_P RSK PDK1_P PDK1 MLL_P MLL HOXA9 GBL_P GBL ERK_P ERK AKT_P AKT**
		**miRNA_1976 miRNA_196B mTOR_P mTOR TSC_1_2 Rictor_P Rictor Rheb_P Rheb RSK_P RSK PDK1_P PDK1 MLL_P MLL HOXA9 GBL_P GBL ERK_P ERK AKT_P AKT**
		**miRNA_1976 miRNA_196B mTOR_P mTOR TSC_1_2 Rictor_P Rictor Rheb_P Rheb RSK_P RSK PDK1_P PDK1 MLL_P MLL HOXA9 GBL_P GBL ERK_P ERK AKT_P AKT**
		**miRNA_1976 miRNA_196B mTOR_P mTOR TSC_1_2 Rictor_P Rictor Rheb_P Rheb RSK_P RSK PDK1_P PDK1 MLL_P MLL HOXA9 GBL_P GBL ERK_P ERK AKT_P AKT**
**3**	**338**	Basin Dimension Percentual: **0.26**
		**miRNA_1976 miRNA_196B mTOR_P mTOR TSC_1_2 Rictor_P Rictor Rheb_P Rheb RSK_P RSK PDK1_P PDK1 MLL_P MLL HOXA9 GBL_P GBL ERK_P ERK AKT_P AKT**
		**miRNA_1976 miRNA_196B mTOR_P mTOR TSC_1_2 Rictor_P Rictor Rheb_P Rheb RSK_P RSK PDK1_P PDK1 MLL_P MLL HOXA9 GBL_P GBL ERK_P ERK AKT_P AKT**
		**miRNA_1976 miRNA_196B mTOR_P mTOR TSC_1_2 Rictor_P Rictor Rheb_P Rheb RSK_P RSK PDK1_P PDK1 MLL_P MLL HOXA9 GBL_P GBL ERK_P ERK AKT_P AKT**
		**miRNA_1976 miRNA_196B mTOR_P mTOR TSC_1_2 Rictor_P Rictor Rheb_P Rheb RSK_P RSK PDK1_P PDK1 MLL_P MLL HOXA9 GBL_P GBL ERK_P ERK AKT_P AKT**
		**miRNA_1976 miRNA_196B mTOR_P mTOR TSC_1_2 Rictor_P Rictor Rheb_P Rheb RSK_P RSK PDK1_P PDK1 MLL_P MLL HOXA9 GBL_P GBL ERK_P ERK AKT_P AKT**
		**miRNA_1976 miRNA_196B mTOR_P mTOR TSC_1_2 Rictor_P Rictor Rheb_P Rheb RSK_P RSK PDK1_P PDK1 MLL_P MLL HOXA9 GBL_P GBL ERK_P ERK AKT_P AKT**
		**miRNA_1976 miRNA_196B mTOR_P mTOR TSC_1_2 Rictor_P Rictor Rheb_P Rheb RSK_P RSK PDK1_P PDK1 MLL_P MLL HOXA9 GBL_P GBL ERK_P ERK AKT_P AKT**
**8**	**334**	Basin Dimension Percentual: **0.26**
		**miRNA_1976 miRNA_196B mTOR_P mTOR TSC_1_2 Rictor_P Rictor Rheb_P Rheb RSK_P RSK PDK1_P PDK1 MLL_P MLL HOXA9 GBL_P GBL ERK_P ERK AKT_P AKT**

		**miRNA_1976 miRNA_196B mTOR_P mTOR TSC_1_2 Rictor_P Rictor Rheb_P Rheb RSK_P RSK PDK1_P PDK1 MLL_P MLL HOXA9 GBL_P GBL ERK_P ERK AKT_P AKT**
		**miRNA_1976 miRNA_196B mTOR_P mTOR TSC_1_2 Rictor_P Rictor Rheb_P Rheb RSK_P RSK PDK1_P PDK1 MLL_P MLL HOXA9 GBL_P GBL ERK_P ERK AKT_P AKT**
		**miRNA_1976 miRNA_196B mTOR_P mTOR TSC_1_2 Rictor_P Rictor Rheb_P Rheb RSK_P RSK PDK1_P PDK1 MLL_P MLL HOXA9 GBL_P GBL ERK_P ERK AKT_P AKT**
		**miRNA_1976 miRNA_196B mTOR_P mTOR TSC_1_2 Rictor_P Rictor Rheb_P Rheb RSK_P RSK PDK1_P PDK1 MLL_P MLL HOXA9 GBL_P GBL ERK_P ERK AKT_P AKT**
		**miRNA_1976 miRNA_196B mTOR_P mTOR TSC_1_2 Rictor_P Rictor Rheb_P Rheb RSK_P RSK PDK1_P PDK1 MLL_P MLL HOXA9 GBL_P GBL ERK_P ERK AKT_P AKT**
		**miRNA_1976 miRNA_196B mTOR_P mTOR TSC_1_2 Rictor_P Rictor Rheb_P Rheb RSK_P RSK PDK1_P PDK1 MLL_P MLL HOXA9 GBL_P GBL ERK_P ERK AKT_P AKT**
**62**	**330**	Basin Dimension Percentual: **0.26**
		**miRNA_1976 miRNA_196B mTOR_P mTOR TSC_1_2 Rictor_P Rictor Rheb_P Rheb RSK_P RSK PDK1_P PDK1 MLL_P MLL HOXA9 GBL_P GBL ERK_P ERK AKT_P AKT**
		**miRNA_1976 miRNA_196B mTOR_P mTOR TSC_1_2 Rictor_P Rictor Rheb_P Rheb RSK_P RSK PDK1_P PDK1 MLL_P MLL HOXA9 GBL_P GBL ERK_P ERK AKT_P AKT**
		**miRNA_1976 miRNA_196B mTOR_P mTOR TSC_1_2 Rictor_P Rictor Rheb_P Rheb RSK_P RSK PDK1_P PDK1 MLL_P MLL HOXA9 GBL_P GBL ERK_P ERK AKT_P AKT**
		**miRNA_1976 miRNA_196B mTOR_P mTOR TSC_1_2 Rictor_P Rictor Rheb_P Rheb RSK_P RSK PDK1_P PDK1 MLL_P MLL HOXA9 GBL_P GBL ERK_P ERK AKT_P AKT**
		**miRNA_1976 miRNA_196B mTOR_P mTOR TSC_1_2 Rictor_P Rictor Rheb_P Rheb RSK_P RSK PDK1_P PDK1 MLL_P MLL HOXA9 GBL_P GBL ERK_P ERK AKT_P AKT**
		**miRNA_1976 miRNA_196B mTOR_P mTOR TSC_1_2 Rictor_P Rictor Rheb_P Rheb RSK_P RSK PDK1_P PDK1 MLL_P MLL HOXA9 GBL_P GBL ERK_P ERK AKT_P AKT**
		**miRNA_1976 miRNA_196B mTOR_P mTOR TSC_1_2 Rictor_P**

		**Rictor Rheb_P Rheb RSK_P RSK PDK1_P PDK1 MLL_P MLL HOXA9 GBL_P GBL ERK_P ERK AKT_P AKT**
**59**	**300**	Basin Dimension Percentual: **0.23**
		**miRNA_1976 miRNA_196B mTOR_P mTOR TSC_1_2 Rictor_P Rictor Rheb_P Rheb RSK_P RSK PDK1_P PDK1 MLL_P MLL HOXA9 GBL_P GBL ERK_P ERK AKT_P AKT**
		**miRNA_1976 miRNA_196B mTOR_P mTOR TSC_1_2 Rictor_P Rictor Rheb_P Rheb RSK_P RSK PDK1_P PDK1 MLL_P MLL HOXA9 GBL_P GBL ERK_P ERK AKT_P AKT**
		**miRNA_1976 miRNA_196B mTOR_P mTOR TSC_1_2 Rictor_P Rictor Rheb_P Rheb RSK_P RSK PDK1_P PDK1 MLL_P MLL HOXA9 GBL_P GBL ERK_P ERK AKT_P AKT**
		**miRNA_1976 miRNA_196B mTOR_P mTOR TSC_1_2 Rictor_P Rictor Rheb_P Rheb RSK_P RSK PDK1_P PDK1 MLL_P MLL HOXA9 GBL_P GBL ERK_P ERK AKT_P AKT**
		**miRNA_1976 miRNA_196B mTOR_P mTOR TSC_1_2 Rictor_P Rictor Rheb_P Rheb RSK_P RSK PDK1_P PDK1 MLL_P MLL HOXA9 GBL_P GBL ERK_P ERK AKT_P AKT**
		**miRNA_1976 miRNA_196B mTOR_P mTOR TSC_1_2 Rictor_P Rictor Rheb_P Rheb RSK_P RSK PDK1_P PDK1 MLL_P MLL HOXA9 GBL_P GBL ERK_P ERK AKT_P AKT**
		**miRNA_1976 miRNA_196B mTOR_P mTOR TSC_1_2 Rictor_P Rictor Rheb_P Rheb RSK_P RSK PDK1_P PDK1 MLL_P MLL HOXA9 GBL_P GBL ERK_P ERK AKT_P AKT**
**78**	**294**	Basin Dimension Percentual: **0.23**
		**miRNA_1976 miRNA_196B mTOR_P mTOR TSC_1_2 Rictor_P Rictor Rheb_P Rheb RSK_P RSK PDK1_P PDK1 MLL_P MLL HOXA9 GBL_P GBL ERK_P ERK AKT_P AKT**
		**miRNA_1976 miRNA_196B mTOR_P mTOR TSC_1_2 Rictor_P Rictor Rheb_P Rheb RSK_P RSK PDK1_P PDK1 MLL_P MLL HOXA9 GBL_P GBL ERK_P ERK AKT_P AKT**
		**miRNA_1976 miRNA_196B mTOR_P mTOR TSC_1_2 Rictor_P Rictor Rheb_P Rheb RSK_P RSK PDK1_P PDK1 MLL_P MLL HOXA9 GBL_P GBL ERK_P ERK AKT_P AKT**
		**miRNA_1976 miRNA_196B mTOR_P mTOR TSC_1_2 Rictor_P Rictor Rheb_P Rheb RSK_P RSK PDK1_P PDK1 MLL_P MLL HOXA9 GBL_P GBL ERK_P ERK AKT_P AKT**
		**miRNA_1976 miRNA_196B mTOR_P mTOR TSC_1_2 Rictor_P Rictor Rheb_P Rheb RSK_P RSK PDK1_P PDK1 MLL_P MLL**

		**HOXA9 GBL_P GBL ERK_P ERK AKT_P AKT**
		**miRNA_1976 miRNA_196B mTOR_P mTOR TSC_1_2 Rictor_P Rictor Rheb_P Rheb RSK_P RSK PDK1_P PDK1 MLL_P MLL HOXA9 GBL_P GBL ERK_P ERK AKT_P AKT**
		**miRNA_1976 miRNA_196B mTOR_P mTOR TSC_1_2 Rictor_P Rictor Rheb_P Rheb RSK_P RSK PDK1_P PDK1 MLL_P MLL HOXA9 GBL_P GBL ERK_P ERK AKT_P AKT**
**65**	**284**	Basin Dimension Percentual: **0.22**
		**miRNA_1976 miRNA_196B mTOR_P mTOR TSC_1_2 Rictor_P Rictor Rheb_P Rheb RSK_P RSK PDK1_P PDK1 MLL_P MLL HOXA9 GBL_P GBL ERK_P ERK AKT_P AKT**
		**miRNA_1976 miRNA_196B mTOR_P mTOR TSC_1_2 Rictor_P Rictor Rheb_P Rheb RSK_P RSK PDK1_P PDK1 MLL_P MLL HOXA9 GBL_P GBL ERK_P ERK AKT_P AKT**
		**miRNA_1976 miRNA_196B mTOR_P mTOR TSC_1_2 Rictor_P Rictor Rheb_P Rheb RSK_P RSK PDK1_P PDK1 MLL_P MLL HOXA9 GBL_P GBL ERK_P ERK AKT_P AKT**
		**miRNA_1976 miRNA_196B mTOR_P mTOR TSC_1_2 Rictor_P Rictor Rheb_P Rheb RSK_P RSK PDK1_P PDK1 MLL_P MLL HOXA9 GBL_P GBL ERK_P ERK AKT_P AKT**
		**miRNA_1976 miRNA_196B mTOR_P mTOR TSC_1_2 Rictor_P Rictor Rheb_P Rheb RSK_P RSK PDK1_P PDK1 MLL_P MLL HOXA9 GBL_P GBL ERK_P ERK AKT_P AKT**
		**miRNA_1976 miRNA_196B mTOR_P mTOR TSC_1_2 Rictor_P Rictor Rheb_P Rheb RSK_P RSK PDK1_P PDK1 MLL_P MLL HOXA9 GBL_P GBL ERK_P ERK AKT_P AKT**
		**miRNA_1976 miRNA_196B mTOR_P mTOR TSC_1_2 Rictor_P Rictor Rheb_P Rheb RSK_P RSK PDK1_P PDK1 MLL_P MLL HOXA9 GBL_P GBL ERK_P ERK AKT_P AKT**
**80**	**252**	Basin Dimension Percentual: **0.20**
		**miRNA_1976 miRNA_196B mTOR_P mTOR TSC_1_2 Rictor_P Rictor Rheb_P Rheb RSK_P RSK PDK1_P PDK1 MLL_P MLL HOXA9 GBL_P GBL ERK_P ERK AKT_P AKT**
		**miRNA_1976 miRNA_196B mTOR_P mTOR TSC_1_2 Rictor_P Rictor Rheb_P Rheb RSK_P RSK PDK1_P PDK1 MLL_P MLL HOXA9 GBL_P GBL ERK_P ERK AKT_P AKT**
		**miRNA_1976 miRNA_196B mTOR_P mTOR TSC_1_2 Rictor_P Rictor Rheb_P Rheb RSK_P RSK PDK1_P PDK1 MLL_P MLL**

		**HOXA9 GBL_P GBL ERK_P ERK AKT_P AKT**
		**miRNA_1976 miRNA_196B mTOR_P mTOR TSC_1_2 Rictor_P**<br>**Rictor Rheb_P Rheb RSK_P RSK PDK1_P PDK1 MLL_P MLL**<br>**HOXA9 GBL_P GBL ERK_P ERK AKT_P AKT**
		**miRNA_1976 miRNA_196B mTOR_P mTOR TSC_1_2 Rictor_P**<br>**Rictor Rheb_P Rheb RSK_P RSK PDK1_P PDK1 MLL_P MLL**<br>**HOXA9 GBL_P GBL ERK_P ERK AKT_P AKT**
		**miRNA_1976 miRNA_196B mTOR_P mTOR TSC_1_2 Rictor_P**<br>**Rictor Rheb_P Rheb RSK_P RSK PDK1_P PDK1 MLL_P MLL**<br>**HOXA9 GBL_P GBL ERK_P ERK AKT_P AKT**
		**miRNA_1976 miRNA_196B mTOR_P mTOR TSC_1_2 Rictor_P**<br>**Rictor Rheb_P Rheb RSK_P RSK PDK1_P PDK1 MLL_P MLL**<br>**HOXA9 GBL_P GBL ERK_P ERK AKT_P AKT**
**14**	**231**	Basin Dimension Percentual: **0.18**
		**miRNA_1976 miRNA_196B mTOR_P mTOR TSC_1_2 Rictor_P**<br>**Rictor Rheb_P Rheb RSK_P RSK PDK1_P PDK1 MLL_P MLL**<br>**HOXA9 GBL_P GBL ERK_P ERK AKT_P AKT**
		**miRNA_1976 miRNA_196B mTOR_P mTOR TSC_1_2 Rictor_P**<br>**Rictor Rheb_P Rheb RSK_P RSK PDK1_P PDK1 MLL_P MLL**<br>**HOXA9 GBL_P GBL ERK_P ERK AKT_P AKT**
		**miRNA_1976 miRNA_196B mTOR_P mTOR TSC_1_2 Rictor_P**<br>**Rictor Rheb_P Rheb RSK_P RSK PDK1_P PDK1 MLL_P MLL**<br>**HOXA9 GBL_P GBL ERK_P ERK AKT_P AKT**
		**miRNA_1976 miRNA_196B mTOR_P mTOR TSC_1_2 Rictor_P**<br>**Rictor Rheb_P Rheb RSK_P RSK PDK1_P PDK1 MLL_P MLL**<br>**HOXA9 GBL_P GBL ERK_P ERK AKT_P AKT**
		**miRNA_1976 miRNA_196B mTOR_P mTOR TSC_1_2 Rictor_P**<br>**Rictor Rheb_P Rheb RSK_P RSK PDK1_P PDK1 MLL_P MLL**<br>**HOXA9 GBL_P GBL ERK_P ERK AKT_P AKT**
		**miRNA_1976 miRNA_196B mTOR_P mTOR TSC_1_2 Rictor_P**<br>**Rictor Rheb_P Rheb RSK_P RSK PDK1_P PDK1 MLL_P MLL**<br>**HOXA9 GBL_P GBL ERK_P ERK AKT_P AKT**
		**miRNA_1976 miRNA_196B mTOR_P mTOR TSC_1_2 Rictor_P**<br>**Rictor Rheb_P Rheb RSK_P RSK PDK1_P PDK1 MLL_P MLL**<br>**HOXA9 GBL_P GBL ERK_P ERK AKT_P AKT**
**26**	**224**	Basin Dimension Percentual: **0.18**
		**miRNA_1976 miRNA_196B mTOR_P mTOR TSC_1_2 Rictor_P**<br>**Rictor Rheb_P Rheb RSK_P RSK PDK1_P PDK1 MLL_P MLL**

		**HOXA9 GBL_P GBL ERK_P ERK AKT_P AKT**
**9**	**219**	Basin Dimension Percentual: **0.17**
		**miRNA_1976 miRNA_196B mTOR_P mTOR TSC_1_2 Rictor_P Rictor Rheb_P Rheb RSK_P RSK PDK1_P PDK1 MLL_P MLL HOXA9 GBL_P GBL ERK_P ERK AKT_P AKT**
		**miRNA_1976 miRNA_196B mTOR_P mTOR TSC_1_2 Rictor_P Rictor Rheb_P Rheb RSK_P RSK PDK1_P PDK1 MLL_P MLL HOXA9 GBL_P GBL ERK_P ERK AKT_P AKT**
		**miRNA_1976 miRNA_196B mTOR_P mTOR TSC_1_2 Rictor_P Rictor Rheb_P Rheb RSK_P RSK PDK1_P PDK1 MLL_P MLL HOXA9 GBL_P GBL ERK_P ERK AKT_P AKT**
		**miRNA_1976 miRNA_196B mTOR_P mTOR TSC_1_2 Rictor_P Rictor Rheb_P Rheb RSK_P RSK PDK1_P PDK1 MLL_P MLL HOXA9 GBL_P GBL ERK_P ERK AKT_P AKT**
		**miRNA_1976 miRNA_196B mTOR_P mTOR TSC_1_2 Rictor_P Rictor Rheb_P Rheb RSK_P RSK PDK1_P PDK1 MLL_P MLL HOXA9 GBL_P GBL ERK_P ERK AKT_P AKT**
		**miRNA_1976 miRNA_196B mTOR_P mTOR TSC_1_2 Rictor_P Rictor Rheb_P Rheb RSK_P RSK PDK1_P PDK1 MLL_P MLL HOXA9 GBL_P GBL ERK_P ERK AKT_P AKT**
		**miRNA_1976 miRNA_196B mTOR_P mTOR TSC_1_2 Rictor_P Rictor Rheb_P Rheb RSK_P RSK PDK1_P PDK1 MLL_P MLL HOXA9 GBL_P GBL ERK_P ERK AKT_P AKT**
**27**	**216**	Basin Dimension Percentual: **0.17**
		**miRNA_1976 miRNA_196B mTOR_P mTOR TSC_1_2 Rictor_P Rictor Rheb_P Rheb RSK_P RSK PDK1_P PDK1 MLL_P MLL HOXA9 GBL_P GBL ERK_P ERK AKT_P AKT**
		**miRNA_1976 miRNA_196B mTOR_P mTOR TSC_1_2 Rictor_P Rictor Rheb_P Rheb RSK_P RSK PDK1_P PDK1 MLL_P MLL HOXA9 GBL_P GBL ERK_P ERK AKT_P AKT**
		**miRNA_1976 miRNA_196B mTOR_P mTOR TSC_1_2 Rictor_P Rictor Rheb_P Rheb RSK_P RSK PDK1_P PDK1 MLL_P MLL HOXA9 GBL_P GBL ERK_P ERK AKT_P AKT**
		**miRNA_1976 miRNA_196B mTOR_P mTOR TSC_1_2 Rictor_P Rictor Rheb_P Rheb RSK_P RSK PDK1_P PDK1 MLL_P MLL HOXA9 GBL_P GBL ERK_P ERK AKT_P AKT**
		**miRNA_1976 miRNA_196B mTOR_P mTOR TSC_1_2 Rictor_P Rictor Rheb_P Rheb RSK_P RSK PDK1_P PDK1 MLL_P MLL**

		**HOXA9 GBL_P GBL ERK_P ERK AKT_P AKT**
		**miRNA_1976 miRNA_196B mTOR_P mTOR TSC_1_2 Rictor_P Rictor Rheb_P Rheb RSK_P RSK PDK1_P PDK1 MLL_P MLL HOXA9 GBL_P GBL ERK_P ERK AKT_P AKT**
		**miRNA_1976 miRNA_196B mTOR_P mTOR TSC_1_2 Rictor_P Rictor Rheb_P Rheb RSK_P RSK PDK1_P PDK1 MLL_P MLL HOXA9 GBL_P GBL ERK_P ERK AKT_P AKT**
**10**	**204**	Basin Dimension Percentual: **0.16**
		**miRNA_1976 miRNA_196B mTOR_P mTOR TSC_1_2 Rictor_P Rictor Rheb_P Rheb RSK_P RSK PDK1_P PDK1 MLL_P MLL HOXA9 GBL_P GBL ERK_P ERK AKT_P AKT**
		**miRNA_1976 miRNA_196B mTOR_P mTOR TSC_1_2 Rictor_P Rictor Rheb_P Rheb RSK_P RSK PDK1_P PDK1 MLL_P MLL HOXA9 GBL_P GBL ERK_P ERK AKT_P AKT**
		**miRNA_1976 miRNA_196B mTOR_P mTOR TSC_1_2 Rictor_P Rictor Rheb_P Rheb RSK_P RSK PDK1_P PDK1 MLL_P MLL HOXA9 GBL_P GBL ERK_P ERK AKT_P AKT**
		**miRNA_1976 miRNA_196B mTOR_P mTOR TSC_1_2 Rictor_P Rictor Rheb_P Rheb RSK_P RSK PDK1_P PDK1 MLL_P MLL HOXA9 GBL_P GBL ERK_P ERK AKT_P AKT**
		**miRNA_1976 miRNA_196B mTOR_P mTOR TSC_1_2 Rictor_P Rictor Rheb_P Rheb RSK_P RSK PDK1_P PDK1 MLL_P MLL HOXA9 GBL_P GBL ERK_P ERK AKT_P AKT**
		**miRNA_1976 miRNA_196B mTOR_P mTOR TSC_1_2 Rictor_P Rictor Rheb_P Rheb RSK_P RSK PDK1_P PDK1 MLL_P MLL HOXA9 GBL_P GBL ERK_P ERK AKT_P AKT**
		**miRNA_1976 miRNA_196B mTOR_P mTOR TSC_1_2 Rictor_P Rictor Rheb_P Rheb RSK_P RSK PDK1_P PDK1 MLL_P MLL HOXA9 GBL_P GBL ERK_P ERK AKT_P AKT**
**2**	**187**	Basin Dimension Percentual: **0.15**
		**miRNA_1976 miRNA_196B mTOR_P mTOR TSC_1_2 Rictor_P Rictor Rheb_P Rheb RSK_P RSK PDK1_P PDK1 MLL_P MLL HOXA9 GBL_P GBL ERK_P ERK AKT_P AKT**
		**miRNA_1976 miRNA_196B mTOR_P mTOR TSC_1_2 Rictor_P Rictor Rheb_P Rheb RSK_P RSK PDK1_P PDK1 MLL_P MLL HOXA9 GBL_P GBL ERK_P ERK AKT_P AKT**
		**miRNA_1976 miRNA_196B mTOR_P mTOR TSC_1_2 Rictor_P Rictor Rheb_P Rheb RSK_P RSK PDK1_P PDK1 MLL_P MLL**

		**HOXA9 GBL_P GBL ERK_P ERK AKT_P AKT**
		**miRNA_1976 miRNA_196B mTOR_P mTOR TSC_1_2 Rictor_P Rictor Rheb_P Rheb RSK_P RSK PDK1_P PDK1 MLL_P MLL HOXA9 GBL_P GBL ERK_P ERK AKT_P AKT**
		**miRNA_1976 miRNA_196B mTOR_P mTOR TSC_1_2 Rictor_P Rictor Rheb_P Rheb RSK_P RSK PDK1_P PDK1 MLL_P MLL HOXA9 GBL_P GBL ERK_P ERK AKT_P AKT**
		**miRNA_1976 miRNA_196B mTOR_P mTOR TSC_1_2 Rictor_P Rictor Rheb_P Rheb RSK_P RSK PDK1_P PDK1 MLL_P MLL HOXA9 GBL_P GBL ERK_P ERK AKT_P AKT**
		**miRNA_1976 miRNA_196B mTOR_P mTOR TSC_1_2 Rictor_P Rictor Rheb_P Rheb RSK_P RSK PDK1_P PDK1 MLL_P MLL HOXA9 GBL_P GBL ERK_P ERK AKT_P AKT**
**35**	**180**	Basin Dimension Percentual: **0.14**
		**miRNA_1976 miRNA_196B mTOR_P mTOR TSC_1_2 Rictor_P Rictor Rheb_P Rheb RSK_P RSK PDK1_P PDK1 MLL_P MLL HOXA9 GBL_P GBL ERK_P ERK AKT_P AKT**
		**miRNA_1976 miRNA_196B mTOR_P mTOR TSC_1_2 Rictor_P Rictor Rheb_P Rheb RSK_P RSK PDK1_P PDK1 MLL_P MLL HOXA9 GBL_P GBL ERK_P ERK AKT_P AKT**
		**miRNA_1976 miRNA_196B mTOR_P mTOR TSC_1_2 Rictor_P Rictor Rheb_P Rheb RSK_P RSK PDK1_P PDK1 MLL_P MLL HOXA9 GBL_P GBL ERK_P ERK AKT_P AKT**
		**miRNA_1976 miRNA_196B mTOR_P mTOR TSC_1_2 Rictor_P Rictor Rheb_P Rheb RSK_P RSK PDK1_P PDK1 MLL_P MLL HOXA9 GBL_P GBL ERK_P ERK AKT_P AKT**
		**miRNA_1976 miRNA_196B mTOR_P mTOR TSC_1_2 Rictor_P Rictor Rheb_P Rheb RSK_P RSK PDK1_P PDK1 MLL_P MLL HOXA9 GBL_P GBL ERK_P ERK AKT_P AKT**
		**miRNA_1976 miRNA_196B mTOR_P mTOR TSC_1_2 Rictor_P Rictor Rheb_P Rheb RSK_P RSK PDK1_P PDK1 MLL_P MLL HOXA9 GBL_P GBL ERK_P ERK AKT_P AKT**
		**miRNA_1976 miRNA_196B mTOR_P mTOR TSC_1_2 Rictor_P Rictor Rheb_P Rheb RSK_P RSK PDK1_P PDK1 MLL_P MLL HOXA9 GBL_P GBL ERK_P ERK AKT_P AKT**
**66**	**168**	Basin Dimension Percentual: **0.13**
		**miRNA_1976 miRNA_196B mTOR_P mTOR TSC_1_2 Rictor_P Rictor Rheb_P Rheb RSK_P RSK PDK1_P PDK1 MLL_P MLL**

		**HOXA9 GBL_P GBL ERK_P ERK AKT_P AKT**
		**miRNA_1976 miRNA_196B mTOR_P mTOR TSC_1_2 Rictor_P Rictor Rheb_P Rheb RSK_P RSK PDK1_P PDK1 MLL_P MLL HOXA9 GBL_P GBL ERK_P ERK AKT_P AKT**
		**miRNA_1976 miRNA_196B mTOR_P mTOR TSC_1_2 Rictor_P Rictor Rheb_P Rheb RSK_P RSK PDK1_P PDK1 MLL_P MLL HOXA9 GBL_P GBL ERK_P ERK AKT_P AKT**
		**miRNA_1976 miRNA_196B mTOR_P mTOR TSC_1_2 Rictor_P Rictor Rheb_P Rheb RSK_P RSK PDK1_P PDK1 MLL_P MLL HOXA9 GBL_P GBL ERK_P ERK AKT_P AKT**
		**miRNA_1976 miRNA_196B mTOR_P mTOR TSC_1_2 Rictor_P Rictor Rheb_P Rheb RSK_P RSK PDK1_P PDK1 MLL_P MLL HOXA9 GBL_P GBL ERK_P ERK AKT_P AKT**
		**miRNA_1976 miRNA_196B mTOR_P mTOR TSC_1_2 Rictor_P Rictor Rheb_P Rheb RSK_P RSK PDK1_P PDK1 MLL_P MLL HOXA9 GBL_P GBL ERK_P ERK AKT_P AKT**
		**miRNA_1976 miRNA_196B mTOR_P mTOR TSC_1_2 Rictor_P Rictor Rheb_P Rheb RSK_P RSK PDK1_P PDK1 MLL_P MLL HOXA9 GBL_P GBL ERK_P ERK AKT_P AKT**
**4**	**167**	Basin Dimension Percentual: **0.13**
		**miRNA_1976 miRNA_196B mTOR_P mTOR TSC_1_2 Rictor_P Rictor Rheb_P Rheb RSK_P RSK PDK1_P PDK1 MLL_P MLL HOXA9 GBL_P GBL ERK_P ERK AKT_P AKT**
		**miRNA_1976 miRNA_196B mTOR_P mTOR TSC_1_2 Rictor_P Rictor Rheb_P Rheb RSK_P RSK PDK1_P PDK1 MLL_P MLL HOXA9 GBL_P GBL ERK_P ERK AKT_P AKT**
		**miRNA_1976 miRNA_196B mTOR_P mTOR TSC_1_2 Rictor_P Rictor Rheb_P Rheb RSK_P RSK PDK1_P PDK1 MLL_P MLL HOXA9 GBL_P GBL ERK_P ERK AKT_P AKT**
		**miRNA_1976 miRNA_196B mTOR_P mTOR TSC_1_2 Rictor_P Rictor Rheb_P Rheb RSK_P RSK PDK1_P PDK1 MLL_P MLL HOXA9 GBL_P GBL ERK_P ERK AKT_P AKT**
		**miRNA_1976 miRNA_196B mTOR_P mTOR TSC_1_2 Rictor_P Rictor Rheb_P Rheb RSK_P RSK PDK1_P PDK1 MLL_P MLL HOXA9 GBL_P GBL ERK_P ERK AKT_P AKT**
		**miRNA_1976 miRNA_196B mTOR_P mTOR TSC_1_2 Rictor_P Rictor Rheb_P Rheb RSK_P RSK PDK1_P PDK1 MLL_P MLL HOXA9 GBL_P GBL ERK_P ERK AKT_P AKT**

		**miRNA_1976 miRNA_196B mTOR_P mTOR TSC_1_2 Rictor_P Rictor Rheb_P Rheb RSK_P RSK PDK1_P PDK1 MLL_P MLL HOXA9 GBL_P GBL ERK_P ERK AKT_P AKT**
**77**	**164**	Basin Dimension Percentual: **0.13**
		**miRNA_1976 miRNA_196B mTOR_P mTOR TSC_1_2 Rictor_P Rictor Rheb_P Rheb RSK_P RSK PDK1_P PDK1 MLL_P MLL HOXA9 GBL_P GBL ERK_P ERK AKT_P AKT**
		**miRNA_1976 miRNA_196B mTOR_P mTOR TSC_1_2 Rictor_P Rictor Rheb_P Rheb RSK_P RSK PDK1_P PDK1 MLL_P MLL HOXA9 GBL_P GBL ERK_P ERK AKT_P AKT**
		**miRNA_1976 miRNA_196B mTOR_P mTOR TSC_1_2 Rictor_P Rictor Rheb_P Rheb RSK_P RSK PDK1_P PDK1 MLL_P MLL HOXA9 GBL_P GBL ERK_P ERK AKT_P AKT**
		**miRNA_1976 miRNA_196B mTOR_P mTOR TSC_1_2 Rictor_P Rictor Rheb_P Rheb RSK_P RSK PDK1_P PDK1 MLL_P MLL HOXA9 GBL_P GBL ERK_P ERK AKT_P AKT**
		**miRNA_1976 miRNA_196B mTOR_P mTOR TSC_1_2 Rictor_P Rictor Rheb_P Rheb RSK_P RSK PDK1_P PDK1 MLL_P MLL HOXA9 GBL_P GBL ERK_P ERK AKT_P AKT**
		**miRNA_1976 miRNA_196B mTOR_P mTOR TSC_1_2 Rictor_P Rictor Rheb_P Rheb RSK_P RSK PDK1_P PDK1 MLL_P MLL HOXA9 GBL_P GBL ERK_P ERK AKT_P AKT**
		**miRNA_1976 miRNA_196B mTOR_P mTOR TSC_1_2 Rictor_P Rictor Rheb_P Rheb RSK_P RSK PDK1_P PDK1 MLL_P MLL HOXA9 GBL_P GBL ERK_P ERK AKT_P AKT**
**75**	**160**	Basin Dimension Percentual: **0.13**
		**miRNA_1976 miRNA_196B mTOR_P mTOR TSC_1_2 Rictor_P Rictor Rheb_P Rheb RSK_P RSK PDK1_P PDK1 MLL_P MLL HOXA9 GBL_P GBL ERK_P ERK AKT_P AKT**
		**miRNA_1976 miRNA_196B mTOR_P mTOR TSC_1_2 Rictor_P Rictor Rheb_P Rheb RSK_P RSK PDK1_P PDK1 MLL_P MLL HOXA9 GBL_P GBL ERK_P ERK AKT_P AKT**
		**miRNA_1976 miRNA_196B mTOR_P mTOR TSC_1_2 Rictor_P Rictor Rheb_P Rheb RSK_P RSK PDK1_P PDK1 MLL_P MLL HOXA9 GBL_P GBL ERK_P ERK AKT_P AKT**
		**miRNA_1976 miRNA_196B mTOR_P mTOR TSC_1_2 Rictor_P Rictor Rheb_P Rheb RSK_P RSK PDK1_P PDK1 MLL_P MLL HOXA9 GBL_P GBL ERK_P ERK AKT_P AKT**

		**miRNA_1976 miRNA_196B mTOR_P mTOR TSC_1_2 Rictor_P Rictor Rheb_P Rheb RSK_P RSK PDK1_P PDK1 MLL_P MLL HOXA9 GBL_P GBL ERK_P ERK AKT_P AKT**
		**miRNA_1976 miRNA_196B mTOR_P mTOR TSC_1_2 Rictor_P Rictor Rheb_P Rheb RSK_P RSK PDK1_P PDK1 MLL_P MLL HOXA9 GBL_P GBL ERK_P ERK AKT_P AKT**
		**miRNA_1976 miRNA_196B mTOR_P mTOR TSC_1_2 Rictor_P Rictor Rheb_P Rheb RSK_P RSK PDK1_P PDK1 MLL_P MLL HOXA9 GBL_P GBL ERK_P ERK AKT_P AKT**
**67**	**154**	Basin Dimension Percentual: **0.12**
		**miRNA_1976 miRNA_196B mTOR_P mTOR TSC_1_2 Rictor_P Rictor Rheb_P Rheb RSK_P RSK PDK1_P PDK1 MLL_P MLL HOXA9 GBL_P GBL ERK_P ERK AKT_P AKT**
		**miRNA_1976 miRNA_196B mTOR_P mTOR TSC_1_2 Rictor_P Rictor Rheb_P Rheb RSK_P RSK PDK1_P PDK1 MLL_P MLL HOXA9 GBL_P GBL ERK_P ERK AKT_P AKT**
		**miRNA_1976 miRNA_196B mTOR_P mTOR TSC_1_2 Rictor_P Rictor Rheb_P Rheb RSK_P RSK PDK1_P PDK1 MLL_P MLL HOXA9 GBL_P GBL ERK_P ERK AKT_P AKT**
		**miRNA_1976 miRNA_196B mTOR_P mTOR TSC_1_2 Rictor_P Rictor Rheb_P Rheb RSK_P RSK PDK1_P PDK1 MLL_P MLL HOXA9 GBL_P GBL ERK_P ERK AKT_P AKT**
		**miRNA_1976 miRNA_196B mTOR_P mTOR TSC_1_2 Rictor_P Rictor Rheb_P Rheb RSK_P RSK PDK1_P PDK1 MLL_P MLL HOXA9 GBL_P GBL ERK_P ERK AKT_P AKT**
		**miRNA_1976 miRNA_196B mTOR_P mTOR TSC_1_2 Rictor_P Rictor Rheb_P Rheb RSK_P RSK PDK1_P PDK1 MLL_P MLL HOXA9 GBL_P GBL ERK_P ERK AKT_P AKT**
		**miRNA_1976 miRNA_196B mTOR_P mTOR TSC_1_2 Rictor_P Rictor Rheb_P Rheb RSK_P RSK PDK1_P PDK1 MLL_P MLL HOXA9 GBL_P GBL ERK_P ERK AKT_P AKT**
**16**	**150**	Basin Dimension Percentual: **0.12**
		**miRNA_1976 miRNA_196B mTOR_P mTOR TSC_1_2 Rictor_P Rictor Rheb_P Rheb RSK_P RSK PDK1_P PDK1 MLL_P MLL HOXA9 GBL_P GBL ERK_P ERK AKT_P AKT**
		**miRNA_1976 miRNA_196B mTOR_P mTOR TSC_1_2 Rictor_P Rictor Rheb_P Rheb RSK_P RSK PDK1_P PDK1 MLL_P MLL HOXA9 GBL_P GBL ERK_P ERK AKT_P AKT**

		**miRNA_1976 miRNA_196B mTOR_P mTOR TSC_1_2 Rictor_P Rictor Rheb_P Rheb RSK_P RSK PDK1_P PDK1 MLL_P MLL HOXA9 GBL_P GBL ERK_P ERK AKT_P AKT**
		**miRNA_1976 miRNA_196B mTOR_P mTOR TSC_1_2 Rictor_P Rictor Rheb_P Rheb RSK_P RSK PDK1_P PDK1 MLL_P MLL HOXA9 GBL_P GBL ERK_P ERK AKT_P AKT**
		**miRNA_1976 miRNA_196B mTOR_P mTOR TSC_1_2 Rictor_P Rictor Rheb_P Rheb RSK_P RSK PDK1_P PDK1 MLL_P MLL HOXA9 GBL_P GBL ERK_P ERK AKT_P AKT**
		**miRNA_1976 miRNA_196B mTOR_P mTOR TSC_1_2 Rictor_P Rictor Rheb_P Rheb RSK_P RSK PDK1_P PDK1 MLL_P MLL HOXA9 GBL_P GBL ERK_P ERK AKT_P AKT**
		**miRNA_1976 miRNA_196B mTOR_P mTOR TSC_1_2 Rictor_P Rictor Rheb_P Rheb RSK_P RSK PDK1_P PDK1 MLL_P MLL HOXA9 GBL_P GBL ERK_P ERK AKT_P AKT**
**21**	**142**	Basin Dimension Percentual: **0.11**
		**miRNA_1976 miRNA_196B mTOR_P mTOR TSC_1_2 Rictor_P Rictor Rheb_P Rheb RSK_P RSK PDK1_P PDK1 MLL_P MLL HOXA9 GBL_P GBL ERK_P ERK AKT_P AKT**
		**miRNA_1976 miRNA_196B mTOR_P mTOR TSC_1_2 Rictor_P Rictor Rheb_P Rheb RSK_P RSK PDK1_P PDK1 MLL_P MLL HOXA9 GBL_P GBL ERK_P ERK AKT_P AKT**
		**miRNA_1976 miRNA_196B mTOR_P mTOR TSC_1_2 Rictor_P Rictor Rheb_P Rheb RSK_P RSK PDK1_P PDK1 MLL_P MLL HOXA9 GBL_P GBL ERK_P ERK AKT_P AKT**
		**miRNA_1976 miRNA_196B mTOR_P mTOR TSC_1_2 Rictor_P Rictor Rheb_P Rheb RSK_P RSK PDK1_P PDK1 MLL_P MLL HOXA9 GBL_P GBL ERK_P ERK AKT_P AKT**
		**miRNA_1976 miRNA_196B mTOR_P mTOR TSC_1_2 Rictor_P Rictor Rheb_P Rheb RSK_P RSK PDK1_P PDK1 MLL_P MLL HOXA9 GBL_P GBL ERK_P ERK AKT_P AKT**
		**miRNA_1976 miRNA_196B mTOR_P mTOR TSC_1_2 Rictor_P Rictor Rheb_P Rheb RSK_P RSK PDK1_P PDK1 MLL_P MLL HOXA9 GBL_P GBL ERK_P ERK AKT_P AKT**
**82**	**138**	Basin Dimension Percentual: **0.11**

		**miRNA_1976 miRNA_196B mTOR_P mTOR TSC_1_2 Rictor_P Rictor Rheb_P Rheb RSK_P RSK PDK1_P PDK1 MLL_P MLL HOXA9 GBL_P GBL ERK_P ERK AKT_P AKT**
		**miRNA_1976 miRNA_196B mTOR_P mTOR TSC_1_2 Rictor_P Rictor Rheb_P Rheb RSK_P RSK PDK1_P PDK1 MLL_P MLL HOXA9 GBL_P GBL ERK_P ERK AKT_P AKT**
		**miRNA_1976 miRNA_196B mTOR_P mTOR TSC_1_2 Rictor_P Rictor Rheb_P Rheb RSK_P RSK PDK1_P PDK1 MLL_P MLL HOXA9 GBL_P GBL ERK_P ERK AKT_P AKT**
		**miRNA_1976 miRNA_196B mTOR_P mTOR TSC_1_2 Rictor_P Rictor Rheb_P Rheb RSK_P RSK PDK1_P PDK1 MLL_P MLL HOXA9 GBL_P GBL ERK_P ERK AKT_P AKT**
		**miRNA_1976 miRNA_196B mTOR_P mTOR TSC_1_2 Rictor_P Rictor Rheb_P Rheb RSK_P RSK PDK1_P PDK1 MLL_P MLL HOXA9 GBL_P GBL ERK_P ERK AKT_P AKT**
		**miRNA_1976 miRNA_196B mTOR_P mTOR TSC_1_2 Rictor_P Rictor Rheb_P Rheb RSK_P RSK PDK1_P PDK1 MLL_P MLL HOXA9 GBL_P GBL ERK_P ERK AKT_P AKT**
		**miRNA_1976 miRNA_196B mTOR_P mTOR TSC_1_2 Rictor_P Rictor Rheb_P Rheb RSK_P RSK PDK1_P PDK1 MLL_P MLL HOXA9 GBL_P GBL ERK_P ERK AKT_P AKT**
**36**	**126**	Basin Dimension Percentual: **0.10**
		**miRNA_1976 miRNA_196B mTOR_P mTOR TSC_1_2 Rictor_P Rictor Rheb_P Rheb RSK_P RSK PDK1_P PDK1 MLL_P MLL HOXA9 GBL_P GBL ERK_P ERK AKT_P AKT**
		**miRNA_1976 miRNA_196B mTOR_P mTOR TSC_1_2 Rictor_P Rictor Rheb_P Rheb RSK_P RSK PDK1_P PDK1 MLL_P MLL HOXA9 GBL_P GBL ERK_P ERK AKT_P AKT**
		**miRNA_1976 miRNA_196B mTOR_P mTOR TSC_1_2 Rictor_P Rictor Rheb_P Rheb RSK_P RSK PDK1_P PDK1 MLL_P MLL HOXA9 GBL_P GBL ERK_P ERK AKT_P AKT**
		**miRNA_1976 miRNA_196B mTOR_P mTOR TSC_1_2 Rictor_P Rictor Rheb_P Rheb RSK_P RSK PDK1_P PDK1 MLL_P MLL HOXA9 GBL_P GBL ERK_P ERK AKT_P AKT**
		**miRNA_1976 miRNA_196B mTOR_P mTOR TSC_1_2 Rictor_P Rictor Rheb_P Rheb RSK_P RSK PDK1_P PDK1 MLL_P MLL HOXA9 GBL_P GBL ERK_P ERK AKT_P AKT**
		**miRNA_1976 miRNA_196B mTOR_P mTOR TSC_1_2 Rictor_P Rictor Rheb_P Rheb RSK_P RSK PDK1_P PDK1 MLL_P MLL**

		**HOXA9 GBL_P GBL ERK_P ERK AKT_P AKT**
		**miRNA_1976 miRNA_196B mTOR_P mTOR TSC_1_2 Rictor_P Rictor Rheb_P Rheb RSK_P RSK PDK1_P PDK1 MLL_P MLL HOXA9 GBL_P GBL ERK_P ERK AKT_P AKT**
**25**	**112**	Basin Dimension Percentual: **0.09**
		**miRNA_1976 miRNA_196B mTOR_P mTOR TSC_1_2 Rictor_P Rictor Rheb_P Rheb RSK_P RSK PDK1_P PDK1 MLL_P MLL HOXA9 GBL_P GBL ERK_P ERK AKT_P AKT**
**84**	**96**	Basin Dimension Percentual: **0.08**
		**miRNA_1976 miRNA_196B mTOR_P mTOR TSC_1_2 Rictor_P Rictor Rheb_P Rheb RSK_P RSK PDK1_P PDK1 MLL_P MLL HOXA9 GBL_P GBL ERK_P ERK AKT_P AKT**
		**miRNA_1976 miRNA_196B mTOR_P mTOR TSC_1_2 Rictor_P Rictor Rheb_P Rheb RSK_P RSK PDK1_P PDK1 MLL_P MLL HOXA9 GBL_P GBL ERK_P ERK AKT_P AKT**
		**miRNA_1976 miRNA_196B mTOR_P mTOR TSC_1_2 Rictor_P Rictor Rheb_P Rheb RSK_P RSK PDK1_P PDK1 MLL_P MLL HOXA9 GBL_P GBL ERK_P ERK AKT_P AKT**
		**miRNA_1976 miRNA_196B mTOR_P mTOR TSC_1_2 Rictor_P Rictor Rheb_P Rheb RSK_P RSK PDK1_P PDK1 MLL_P MLL HOXA9 GBL_P GBL ERK_P ERK AKT_P AKT**
		**miRNA_1976 miRNA_196B mTOR_P mTOR TSC_1_2 Rictor_P Rictor Rheb_P Rheb RSK_P RSK PDK1_P PDK1 MLL_P MLL HOXA9 GBL_P GBL ERK_P ERK AKT_P AKT**
		**miRNA_1976 miRNA_196B mTOR_P mTOR TSC_1_2 Rictor_P Rictor Rheb_P Rheb RSK_P RSK PDK1_P PDK1 MLL_P MLL HOXA9 GBL_P GBL ERK_P ERK AKT_P AKT**
		**miRNA_1976 miRNA_196B mTOR_P mTOR TSC_1_2 Rictor_P Rictor Rheb_P Rheb RSK_P RSK PDK1_P PDK1 MLL_P MLL HOXA9 GBL_P GBL ERK_P ERK AKT_P AKT**
**64**	**94**	Basin Dimension Percentual: **0.07**
		**miRNA_1976 miRNA_196B mTOR_P mTOR TSC_1_2 Rictor_P Rictor Rheb_P Rheb RSK_P RSK PDK1_P PDK1 MLL_P MLL HOXA9 GBL_P GBL ERK_P ERK AKT_P AKT**
		**miRNA_1976 miRNA_196B mTOR_P mTOR TSC_1_2 Rictor_P Rictor Rheb_P Rheb RSK_P RSK PDK1_P PDK1 MLL_P MLL HOXA9 GBL_P GBL ERK_P ERK AKT_P AKT**
		**miRNA_1976 miRNA_196B mTOR_P mTOR TSC_1_2 Rictor_P**

		**Rictor Rheb_P Rheb RSK_P RSK PDK1_P PDK1 MLL_P MLL HOXA9 GBL_P GBL ERK_P ERK AKT_P AKT**
		**miRNA_1976 miRNA_196B mTOR_P mTOR TSC_1_2 Rictor_P Rictor Rheb_P Rheb RSK_P RSK PDK1_P PDK1 MLL_P MLL HOXA9 GBL_P GBL ERK_P ERK AKT_P AKT**
		**miRNA_1976 miRNA_196B mTOR_P mTOR TSC_1_2 Rictor_P Rictor Rheb_P Rheb RSK_P RSK PDK1_P PDK1 MLL_P MLL HOXA9 GBL_P GBL ERK_P ERK AKT_P AKT**
		**miRNA_1976 miRNA_196B mTOR_P mTOR TSC_1_2 Rictor_P Rictor Rheb_P Rheb RSK_P RSK PDK1_P PDK1 MLL_P MLL HOXA9 GBL_P GBL ERK_P ERK AKT_P AKT**
		**miRNA_1976 miRNA_196B mTOR_P mTOR TSC_1_2 Rictor_P Rictor Rheb_P Rheb RSK_P RSK PDK1_P PDK1 MLL_P MLL HOXA9 GBL_P GBL ERK_P ERK AKT_P AKT**
**68**	**92**	Basin Dimension Percentual: **0.07**
		**miRNA_1976 miRNA_196B mTOR_P mTOR TSC_1_2 Rictor_P Rictor Rheb_P Rheb RSK_P RSK PDK1_P PDK1 MLL_P MLL HOXA9 GBL_P GBL ERK_P ERK AKT_P AKT**
		**miRNA_1976 miRNA_196B mTOR_P mTOR TSC_1_2 Rictor_P Rictor Rheb_P Rheb RSK_P RSK PDK1_P PDK1 MLL_P MLL HOXA9 GBL_P GBL ERK_P ERK AKT_P AKT**
		**miRNA_1976 miRNA_196B mTOR_P mTOR TSC_1_2 Rictor_P Rictor Rheb_P Rheb RSK_P RSK PDK1_P PDK1 MLL_P MLL HOXA9 GBL_P GBL ERK_P ERK AKT_P AKT**
		**miRNA_1976 miRNA_196B mTOR_P mTOR TSC_1_2 Rictor_P Rictor Rheb_P Rheb RSK_P RSK PDK1_P PDK1 MLL_P MLL HOXA9 GBL_P GBL ERK_P ERK AKT_P AKT**
		**miRNA_1976 miRNA_196B mTOR_P mTOR TSC_1_2 Rictor_P Rictor Rheb_P Rheb RSK_P RSK PDK1_P PDK1 MLL_P MLL HOXA9 GBL_P GBL ERK_P ERK AKT_P AKT**
		**miRNA_1976 miRNA_196B mTOR_P mTOR TSC_1_2 Rictor_P Rictor Rheb_P Rheb RSK_P RSK PDK1_P PDK1 MLL_P MLL HOXA9 GBL_P GBL ERK_P ERK AKT_P AKT**
		**miRNA_1976 miRNA_196B mTOR_P mTOR TSC_1_2 Rictor_P Rictor Rheb_P Rheb RSK_P RSK PDK1_P PDK1 MLL_P MLL HOXA9 GBL_P GBL ERK_P ERK AKT_P AKT**
**33**	**90**	Basin Dimension Percentual: **0.07**
		**miRNA_1976 miRNA_196B mTOR_P mTOR TSC_1_2 Rictor_P Rictor Rheb_P Rheb RSK_P RSK PDK1_P PDK1 MLL_P MLL**

		**HOXA9 GBL_P GBL ERK_P ERK AKT_P AKT**
		**miRNA_1976 miRNA_196B mTOR_P mTOR TSC_1_2 Rictor_P Rictor Rheb_P Rheb RSK_P RSK PDK1_P PDK1 MLL_P MLL HOXA9 GBL_P GBL ERK_P ERK AKT_P AKT**
		**miRNA_1976 miRNA_196B mTOR_P mTOR TSC_1_2 Rictor_P Rictor Rheb_P Rheb RSK_P RSK PDK1_P PDK1 MLL_P MLL HOXA9 GBL_P GBL ERK_P ERK AKT_P AKT**
		**miRNA_1976 miRNA_196B mTOR_P mTOR TSC_1_2 Rictor_P Rictor Rheb_P Rheb RSK_P RSK PDK1_P PDK1 MLL_P MLL HOXA9 GBL_P GBL ERK_P ERK AKT_P AKT**
		**miRNA_1976 miRNA_196B mTOR_P mTOR TSC_1_2 Rictor_P Rictor Rheb_P Rheb RSK_P RSK PDK1_P PDK1 MLL_P MLL HOXA9 GBL_P GBL ERK_P ERK AKT_P AKT**
		**miRNA_1976 miRNA_196B mTOR_P mTOR TSC_1_2 Rictor_P Rictor Rheb_P Rheb RSK_P RSK PDK1_P PDK1 MLL_P MLL HOXA9 GBL_P GBL ERK_P ERK AKT_P AKT**
		**miRNA_1976 miRNA_196B mTOR_P mTOR TSC_1_2 Rictor_P Rictor Rheb_P Rheb RSK_P RSK PDK1_P PDK1 MLL_P MLL HOXA9 GBL_P GBL ERK_P ERK AKT_P AKT**
**22**	**84**	Basin Dimension Percentual: **0.07**
		**miRNA_1976 miRNA_196B mTOR_P mTOR TSC_1_2 Rictor_P Rictor Rheb_P Rheb RSK_P RSK PDK1_P PDK1 MLL_P MLL HOXA9 GBL_P GBL ERK_P ERK AKT_P AKT**
		**miRNA_1976 miRNA_196B mTOR_P mTOR TSC_1_2 Rictor_P Rictor Rheb_P Rheb RSK_P RSK PDK1_P PDK1 MLL_P MLL HOXA9 GBL_P GBL ERK_P ERK AKT_P AKT**
		**miRNA_1976 miRNA_196B mTOR_P mTOR TSC_1_2 Rictor_P Rictor Rheb_P Rheb RSK_P RSK PDK1_P PDK1 MLL_P MLL HOXA9 GBL_P GBL ERK_P ERK AKT_P AKT**
		**miRNA_1976 miRNA_196B mTOR_P mTOR TSC_1_2 Rictor_P Rictor Rheb_P Rheb RSK_P RSK PDK1_P PDK1 MLL_P MLL HOXA9 GBL_P GBL ERK_P ERK AKT_P AKT**
		**miRNA_1976 miRNA_196B mTOR_P mTOR TSC_1_2 Rictor_P Rictor Rheb_P Rheb RSK_P RSK PDK1_P PDK1 MLL_P MLL HOXA9 GBL_P GBL ERK_P ERK AKT_P AKT**
		**miRNA_1976 miRNA_196B mTOR_P mTOR TSC_1_2 Rictor_P Rictor Rheb_P Rheb RSK_P RSK PDK1_P PDK1 MLL_P MLL HOXA9 GBL_P GBL ERK_P ERK AKT_P AKT**

		**miRNA_1976 miRNA_196B mTOR_P mTOR TSC_1_2 Rictor_P Rictor Rheb_P Rheb RSK_P RSK PDK1_P PDK1 MLL_P MLL HOXA9 GBL_P GBL ERK_P ERK AKT_P AKT**
**32**	**82**	Basin Dimension Percentual: **0.06**
		**miRNA_1976 miRNA_196B mTOR_P mTOR TSC_1_2 Rictor_P Rictor Rheb_P Rheb RSK_P RSK PDK1_P PDK1 MLL_P MLL HOXA9 GBL_P GBL ERK_P ERK AKT_P AKT**
		**miRNA_1976 miRNA_196B mTOR_P mTOR TSC_1_2 Rictor_P Rictor Rheb_P Rheb RSK_P RSK PDK1_P PDK1 MLL_P MLL HOXA9 GBL_P GBL ERK_P ERK AKT_P AKT**
		**miRNA_1976 miRNA_196B mTOR_P mTOR TSC_1_2 Rictor_P Rictor Rheb_P Rheb RSK_P RSK PDK1_P PDK1 MLL_P MLL HOXA9 GBL_P GBL ERK_P ERK AKT_P AKT**
		**miRNA_1976 miRNA_196B mTOR_P mTOR TSC_1_2 Rictor_P Rictor Rheb_P Rheb RSK_P RSK PDK1_P PDK1 MLL_P MLL HOXA9 GBL_P GBL ERK_P ERK AKT_P AKT**
		**miRNA_1976 miRNA_196B mTOR_P mTOR TSC_1_2 Rictor_P Rictor Rheb_P Rheb RSK_P RSK PDK1_P PDK1 MLL_P MLL HOXA9 GBL_P GBL ERK_P ERK AKT_P AKT**
		**miRNA_1976 miRNA_196B mTOR_P mTOR TSC_1_2 Rictor_P Rictor Rheb_P Rheb RSK_P RSK PDK1_P PDK1 MLL_P MLL HOXA9 GBL_P GBL ERK_P ERK AKT_P AKT**
		**miRNA_1976 miRNA_196B mTOR_P mTOR TSC_1_2 Rictor_P Rictor Rheb_P Rheb RSK_P RSK PDK1_P PDK1 MLL_P MLL HOXA9 GBL_P GBL ERK_P ERK AKT_P AKT**
**30**	**80**	Basin Dimension Percentual: **0.06**
		**miRNA_1976 miRNA_196B mTOR_P mTOR TSC_1_2 Rictor_P Rictor Rheb_P Rheb RSK_P RSK PDK1_P PDK1 MLL_P MLL HOXA9 GBL_P GBL ERK_P ERK AKT_P AKT**
		**miRNA_1976 miRNA_196B mTOR_P mTOR TSC_1_2 Rictor_P Rictor Rheb_P Rheb RSK_P RSK PDK1_P PDK1 MLL_P MLL HOXA9 GBL_P GBL ERK_P ERK AKT_P AKT**
		**miRNA_1976 miRNA_196B mTOR_P mTOR TSC_1_2 Rictor_P Rictor Rheb_P Rheb RSK_P RSK PDK1_P PDK1 MLL_P MLL HOXA9 GBL_P GBL ERK_P ERK AKT_P AKT**
		**miRNA_1976 miRNA_196B mTOR_P mTOR TSC_1_2 Rictor_P Rictor Rheb_P Rheb RSK_P RSK PDK1_P PDK1 MLL_P MLL HOXA9 GBL_P GBL ERK_P ERK AKT_P AKT**
		**miRNA_1976 miRNA_196B mTOR_P mTOR TSC_1_2 Rictor_P**

		**Rictor Rheb_P Rheb RSK_P RSK PDK1_P PDK1 MLL_P MLL HOXA9 GBL_P GBL ERK_P ERK AKT_P AKT**
		**miRNA_1976 miRNA_196B mTOR_P mTOR TSC_1_2 Rictor_P Rictor Rheb_P Rheb RSK_P RSK PDK1_P PDK1 MLL_P MLL HOXA9 GBL_P GBL ERK_P ERK AKT_P AKT**
		**miRNA_1976 miRNA_196B mTOR_P mTOR TSC_1_2 Rictor_P Rictor Rheb_P Rheb RSK_P RSK PDK1_P PDK1 MLL_P MLL HOXA9 GBL_P GBL ERK_P ERK AKT_P AKT**
**85**	**80**	Basin Dimension Percentual: **0.06**
		**miRNA_1976 miRNA_196B mTOR_P mTOR TSC_1_2 Rictor_P Rictor Rheb_P Rheb RSK_P RSK PDK1_P PDK1 MLL_P MLL HOXA9 GBL_P GBL ERK_P ERK AKT_P AKT**
		**miRNA_1976 miRNA_196B mTOR_P mTOR TSC_1_2 Rictor_P Rictor Rheb_P Rheb RSK_P RSK PDK1_P PDK1 MLL_P MLL HOXA9 GBL_P GBL ERK_P ERK AKT_P AKT**
		**miRNA_1976 miRNA_196B mTOR_P mTOR TSC_1_2 Rictor_P Rictor Rheb_P Rheb RSK_P RSK PDK1_P PDK1 MLL_P MLL HOXA9 GBL_P GBL ERK_P ERK AKT_P AKT**
		**miRNA_1976 miRNA_196B mTOR_P mTOR TSC_1_2 Rictor_P Rictor Rheb_P Rheb RSK_P RSK PDK1_P PDK1 MLL_P MLL HOXA9 GBL_P GBL ERK_P ERK AKT_P AKT**
		**miRNA_1976 miRNA_196B mTOR_P mTOR TSC_1_2 Rictor_P Rictor Rheb_P Rheb RSK_P RSK PDK1_P PDK1 MLL_P MLL HOXA9 GBL_P GBL ERK_P ERK AKT_P AKT**
		**miRNA_1976 miRNA_196B mTOR_P mTOR TSC_1_2 Rictor_P Rictor Rheb_P Rheb RSK_P RSK PDK1_P PDK1 MLL_P MLL HOXA9 GBL_P GBL ERK_P ERK AKT_P AKT**
		**miRNA_1976 miRNA_196B mTOR_P mTOR TSC_1_2 Rictor_P Rictor Rheb_P Rheb RSK_P RSK PDK1_P PDK1 MLL_P MLL HOXA9 GBL_P GBL ERK_P ERK AKT_P AKT**
**13**	**75**	Basin Dimension Percentual: **0.06**
		**miRNA_1976 miRNA_196B mTOR_P mTOR TSC_1_2 Rictor_P Rictor Rheb_P Rheb RSK_P RSK PDK1_P PDK1 MLL_P MLL HOXA9 GBL_P GBL ERK_P ERK AKT_P AKT**
		**miRNA_1976 miRNA_196B mTOR_P mTOR TSC_1_2 Rictor_P Rictor Rheb_P Rheb RSK_P RSK PDK1_P PDK1 MLL_P MLL HOXA9 GBL_P GBL ERK_P ERK AKT_P AKT**
		**miRNA_1976 miRNA_196B mTOR_P mTOR TSC_1_2 Rictor_P**

		**Rictor Rheb_P Rheb RSK_P RSK PDK1_P PDK1 MLL_P MLL HOXA9 GBL_P GBL ERK_P ERK AKT_P AKT**
		**miRNA_1976 miRNA_196B mTOR_P mTOR TSC_1_2 Rictor_P Rictor Rheb_P Rheb RSK_P RSK PDK1_P PDK1 MLL_P MLL HOXA9 GBL_P GBL ERK_P ERK AKT_P AKT**
		**miRNA_1976 miRNA_196B mTOR_P mTOR TSC_1_2 Rictor_P Rictor Rheb_P Rheb RSK_P RSK PDK1_P PDK1 MLL_P MLL HOXA9 GBL_P GBL ERK_P ERK AKT_P AKT**
		**miRNA_1976 miRNA_196B mTOR_P mTOR TSC_1_2 Rictor_P Rictor Rheb_P Rheb RSK_P RSK PDK1_P PDK1 MLL_P MLL HOXA9 GBL_P GBL ERK_P ERK AKT_P AKT**
		**miRNA_1976 miRNA_196B mTOR_P mTOR TSC_1_2 Rictor_P Rictor Rheb_P Rheb RSK_P RSK PDK1_P PDK1 MLL_P MLL HOXA9 GBL_P GBL ERK_P ERK AKT_P AKT**
**83**	**72**	Basin Dimension Percentual: **0.06**
		**miRNA_1976 miRNA_196B mTOR_P mTOR TSC_1_2 Rictor_P Rictor Rheb_P Rheb RSK_P RSK PDK1_P PDK1 MLL_P MLL HOXA9 GBL_P GBL ERK_P ERK AKT_P AKT**
		**miRNA_1976 miRNA_196B mTOR_P mTOR TSC_1_2 Rictor_P Rictor Rheb_P Rheb RSK_P RSK PDK1_P PDK1 MLL_P MLL HOXA9 GBL_P GBL ERK_P ERK AKT_P AKT**
		**miRNA_1976 miRNA_196B mTOR_P mTOR TSC_1_2 Rictor_P Rictor Rheb_P Rheb RSK_P RSK PDK1_P PDK1 MLL_P MLL HOXA9 GBL_P GBL ERK_P ERK AKT_P AKT**
		**miRNA_1976 miRNA_196B mTOR_P mTOR TSC_1_2 Rictor_P Rictor Rheb_P Rheb RSK_P RSK PDK1_P PDK1 MLL_P MLL HOXA9 GBL_P GBL ERK_P ERK AKT_P AKT**
		**miRNA_1976 miRNA_196B mTOR_P mTOR TSC_1_2 Rictor_P Rictor Rheb_P Rheb RSK_P RSK PDK1_P PDK1 MLL_P MLL HOXA9 GBL_P GBL ERK_P ERK AKT_P AKT**
		**miRNA_1976 miRNA_196B mTOR_P mTOR TSC_1_2 Rictor_P Rictor Rheb_P Rheb RSK_P RSK PDK1_P PDK1 MLL_P MLL HOXA9 GBL_P GBL ERK_P ERK AKT_P AKT**
		**miRNA_1976 miRNA_196B mTOR_P mTOR TSC_1_2 Rictor_P Rictor Rheb_P Rheb RSK_P RSK PDK1_P PDK1 MLL_P MLL HOXA9 GBL_P GBL ERK_P ERK AKT_P AKT**
**19**	**71**	Basin Dimension Percentual: **0.06**
		**miRNA_1976 miRNA_196B mTOR_P mTOR TSC_1_2 Rictor_P Rictor Rheb_P Rheb RSK_P RSK PDK1_P PDK1 MLL_P MLL**

		**HOXA9 GBL_P GBL ERK_P ERK AKT_P AKT**
		**miRNA_1976 miRNA_196B mTOR_P mTOR TSC_1_2 Rictor_P Rictor Rheb_P Rheb RSK_P RSK PDK1_P PDK1 MLL_P MLL HOXA9 GBL_P GBL ERK_P ERK AKT_P AKT**
		**miRNA_1976 miRNA_196B mTOR_P mTOR TSC_1_2 Rictor_P Rictor Rheb_P Rheb RSK_P RSK PDK1_P PDK1 MLL_P MLL HOXA9 GBL_P GBL ERK_P ERK AKT_P AKT**
		**miRNA_1976 miRNA_196B mTOR_P mTOR TSC_1_2 Rictor_P Rictor Rheb_P Rheb RSK_P RSK PDK1_P PDK1 MLL_P MLL HOXA9 GBL_P GBL ERK_P ERK AKT_P AKT**
		**miRNA_1976 miRNA_196B mTOR_P mTOR TSC_1_2 Rictor_P Rictor Rheb_P Rheb RSK_P RSK PDK1_P PDK1 MLL_P MLL HOXA9 GBL_P GBL ERK_P ERK AKT_P AKT**
		**miRNA_1976 miRNA_196B mTOR_P mTOR TSC_1_2 Rictor_P Rictor Rheb_P Rheb RSK_P RSK PDK1_P PDK1 MLL_P MLL HOXA9 GBL_P GBL ERK_P ERK AKT_P AKT**
		**miRNA_1976 miRNA_196B mTOR_P mTOR TSC_1_2 Rictor_P Rictor Rheb_P Rheb RSK_P RSK PDK1_P PDK1 MLL_P MLL HOXA9 GBL_P GBL ERK_P ERK AKT_P AKT**
**61**	**68**	Basin Dimension Percentual: **0.05**
		**miRNA_1976 miRNA_196B mTOR_P mTOR TSC_1_2 Rictor_P Rictor Rheb_P Rheb RSK_P RSK PDK1_P PDK1 MLL_P MLL HOXA9 GBL_P GBL ERK_P ERK AKT_P AKT**
		**miRNA_1976 miRNA_196B mTOR_P mTOR TSC_1_2 Rictor_P Rictor Rheb_P Rheb RSK_P RSK PDK1_P PDK1 MLL_P MLL HOXA9 GBL_P GBL ERK_P ERK AKT_P AKT**
		**miRNA_1976 miRNA_196B mTOR_P mTOR TSC_1_2 Rictor_P Rictor Rheb_P Rheb RSK_P RSK PDK1_P PDK1 MLL_P MLL HOXA9 GBL_P GBL ERK_P ERK AKT_P AKT**
		**miRNA_1976 miRNA_196B mTOR_P mTOR TSC_1_2 Rictor_P Rictor Rheb_P Rheb RSK_P RSK PDK1_P PDK1 MLL_P MLL HOXA9 GBL_P GBL ERK_P ERK AKT_P AKT**
		**miRNA_1976 miRNA_196B mTOR_P mTOR TSC_1_2 Rictor_P Rictor Rheb_P Rheb RSK_P RSK PDK1_P PDK1 MLL_P MLL HOXA9 GBL_P GBL ERK_P ERK AKT_P AKT**
		**miRNA_1976 miRNA_196B mTOR_P mTOR TSC_1_2 Rictor_P Rictor Rheb_P Rheb RSK_P RSK PDK1_P PDK1 MLL_P MLL HOXA9 GBL_P GBL ERK_P ERK AKT_P AKT**

		**miRNA_1976 miRNA_196B mTOR_P mTOR TSC_1_2 Rictor_P Rictor Rheb_P Rheb RSK_P RSK PDK1_P PDK1 MLL_P MLL HOXA9 GBL_P GBL ERK_P ERK AKT_P AKT**
**34**	**63**	Basin Dimension Percentual: **0.05**
		**miRNA_1976 miRNA_196B mTOR_P mTOR TSC_1_2 Rictor_P Rictor Rheb_P Rheb RSK_P RSK PDK1_P PDK1 MLL_P MLL HOXA9 GBL_P GBL ERK_P ERK AKT_P AKT**
		**miRNA_1976 miRNA_196B mTOR_P mTOR TSC_1_2 Rictor_P Rictor Rheb_P Rheb RSK_P RSK PDK1_P PDK1 MLL_P MLL HOXA9 GBL_P GBL ERK_P ERK AKT_P AKT**
		**miRNA_1976 miRNA_196B mTOR_P mTOR TSC_1_2 Rictor_P Rictor Rheb_P Rheb RSK_P RSK PDK1_P PDK1 MLL_P MLL HOXA9 GBL_P GBL ERK_P ERK AKT_P AKT**
		**miRNA_1976 miRNA_196B mTOR_P mTOR TSC_1_2 Rictor_P Rictor Rheb_P Rheb RSK_P RSK PDK1_P PDK1 MLL_P MLL HOXA9 GBL_P GBL ERK_P ERK AKT_P AKT**
		**miRNA_1976 miRNA_196B mTOR_P mTOR TSC_1_2 Rictor_P Rictor Rheb_P Rheb RSK_P RSK PDK1_P PDK1 MLL_P MLL HOXA9 GBL_P GBL ERK_P ERK AKT_P AKT**
		**miRNA_1976 miRNA_196B mTOR_P mTOR TSC_1_2 Rictor_P Rictor Rheb_P Rheb RSK_P RSK PDK1_P PDK1 MLL_P MLL HOXA9 GBL_P GBL ERK_P ERK AKT_P AKT**
		**miRNA_1976 miRNA_196B mTOR_P mTOR TSC_1_2 Rictor_P Rictor Rheb_P Rheb RSK_P RSK PDK1_P PDK1 MLL_P MLL HOXA9 GBL_P GBL ERK_P ERK AKT_P AKT**
**86**	**44**	Basin Dimension Percentual: **0.03**
		**miRNA_1976 miRNA_196B mTOR_P mTOR TSC_1_2 Rictor_P Rictor Rheb_P Rheb RSK_P RSK PDK1_P PDK1 MLL_P MLL HOXA9 GBL_P GBL ERK_P ERK AKT_P AKT**
		**miRNA_1976 miRNA_196B mTOR_P mTOR TSC_1_2 Rictor_P Rictor Rheb_P Rheb RSK_P RSK PDK1_P PDK1 MLL_P MLL HOXA9 GBL_P GBL ERK_P ERK AKT_P AKT**
		**miRNA_1976 miRNA_196B mTOR_P mTOR TSC_1_2 Rictor_P Rictor Rheb_P Rheb RSK_P RSK PDK1_P PDK1 MLL_P MLL HOXA9 GBL_P GBL ERK_P ERK AKT_P AKT**
		**miRNA_1976 miRNA_196B mTOR_P mTOR TSC_1_2 Rictor_P Rictor Rheb_P Rheb RSK_P RSK PDK1_P PDK1 MLL_P MLL HOXA9 GBL_P GBL ERK_P ERK AKT_P AKT**

		**miRNA_1976 miRNA_196B mTOR_P mTOR TSC_1_2 Rictor_P Rictor Rheb_P Rheb RSK_P RSK PDK1_P PDK1 MLL_P MLL HOXA9 GBL_P GBL ERK_P ERK AKT_P AKT**
		**miRNA_1976 miRNA_196B mTOR_P mTOR TSC_1_2 Rictor_P Rictor Rheb_P Rheb RSK_P RSK PDK1_P PDK1 MLL_P MLL HOXA9 GBL_P GBL ERK_P ERK AKT_P AKT**
		**miRNA_1976 miRNA_196B mTOR_P mTOR TSC_1_2 Rictor_P Rictor Rheb_P Rheb RSK_P RSK PDK1_P PDK1 MLL_P MLL HOXA9 GBL_P GBL ERK_P ERK AKT_P AKT**
**20**	**42**	Basin Dimension Percentual: **0.03**
		**miRNA_1976 miRNA_196B mTOR_P mTOR TSC_1_2 Rictor_P Rictor Rheb_P Rheb RSK_P RSK PDK1_P PDK1 MLL_P MLL HOXA9 GBL_P GBL ERK_P ERK AKT_P AKT**
		**miRNA_1976 miRNA_196B mTOR_P mTOR TSC_1_2 Rictor_P Rictor Rheb_P Rheb RSK_P RSK PDK1_P PDK1 MLL_P MLL HOXA9 GBL_P GBL ERK_P ERK AKT_P AKT**
		**miRNA_1976 miRNA_196B mTOR_P mTOR TSC_1_2 Rictor_P Rictor Rheb_P Rheb RSK_P RSK PDK1_P PDK1 MLL_P MLL HOXA9 GBL_P GBL ERK_P ERK AKT_P AKT**
		**miRNA_1976 miRNA_196B mTOR_P mTOR TSC_1_2 Rictor_P Rictor Rheb_P Rheb RSK_P RSK PDK1_P PDK1 MLL_P MLL HOXA9 GBL_P GBL ERK_P ERK AKT_P AKT**
		**miRNA_1976 miRNA_196B mTOR_P mTOR TSC_1_2 Rictor_P Rictor Rheb_P Rheb RSK_P RSK PDK1_P PDK1 MLL_P MLL HOXA9 GBL_P GBL ERK_P ERK AKT_P AKT**
		**miRNA_1976 miRNA_196B mTOR_P mTOR TSC_1_2 Rictor_P Rictor Rheb_P Rheb RSK_P RSK PDK1_P PDK1 MLL_P MLL HOXA9 GBL_P GBL ERK_P ERK AKT_P AKT**
		**miRNA_1976 miRNA_196B mTOR_P mTOR TSC_1_2 Rictor_P Rictor Rheb_P Rheb RSK_P RSK PDK1_P PDK1 MLL_P MLL HOXA9 GBL_P GBL ERK_P ERK AKT_P AKT**
**31**	**41**	Basin Dimension Percentual: **0.03**
		**miRNA_1976 miRNA_196B mTOR_P mTOR TSC_1_2 Rictor_P Rictor Rheb_P Rheb RSK_P RSK PDK1_P PDK1 MLL_P MLL HOXA9 GBL_P GBL ERK_P ERK AKT_P AKT**
		**miRNA_1976 miRNA_196B mTOR_P mTOR TSC_1_2 Rictor_P Rictor Rheb_P Rheb RSK_P RSK PDK1_P PDK1 MLL_P MLL HOXA9 GBL_P GBL ERK_P ERK AKT_P AKT**

		**miRNA_1976 miRNA_196B mTOR_P mTOR TSC_1_2 Rictor_P Rictor Rheb_P Rheb RSK_P RSK PDK1_P PDK1 MLL_P MLL HOXA9 GBL_P GBL ERK_P ERK AKT_P AKT**
		**miRNA_1976 miRNA_196B mTOR_P mTOR TSC_1_2 Rictor_P Rictor Rheb_P Rheb RSK_P RSK PDK1_P PDK1 MLL_P MLL HOXA9 GBL_P GBL ERK_P ERK AKT_P AKT**
		**miRNA_1976 miRNA_196B mTOR_P mTOR TSC_1_2 Rictor_P Rictor Rheb_P Rheb RSK_P RSK PDK1_P PDK1 MLL_P MLL HOXA9 GBL_P GBL ERK_P ERK AKT_P AKT**
		**miRNA_1976 miRNA_196B mTOR_P mTOR TSC_1_2 Rictor_P Rictor Rheb_P Rheb RSK_P RSK PDK1_P PDK1 MLL_P MLL HOXA9 GBL_P GBL ERK_P ERK AKT_P AKT**
		**miRNA_1976 miRNA_196B mTOR_P mTOR TSC_1_2 Rictor_P Rictor Rheb_P Rheb RSK_P RSK PDK1_P PDK1 MLL_P MLL HOXA9 GBL_P GBL ERK_P ERK AKT_P AKT**
**29**	**40**	Basin Dimension Percentual: **0.03**
		**miRNA_1976 miRNA_196B mTOR_P mTOR TSC_1_2 Rictor_P Rictor Rheb_P Rheb RSK_P RSK PDK1_P PDK1 MLL_P MLL HOXA9 GBL_P GBL ERK_P ERK AKT_P AKT**
		**miRNA_1976 miRNA_196B mTOR_P mTOR TSC_1_2 Rictor_P Rictor Rheb_P Rheb RSK_P RSK PDK1_P PDK1 MLL_P MLL HOXA9 GBL_P GBL ERK_P ERK AKT_P AKT**
		**miRNA_1976 miRNA_196B mTOR_P mTOR TSC_1_2 Rictor_P Rictor Rheb_P Rheb RSK_P RSK PDK1_P PDK1 MLL_P MLL HOXA9 GBL_P GBL ERK_P ERK AKT_P AKT**
		**miRNA_1976 miRNA_196B mTOR_P mTOR TSC_1_2 Rictor_P Rictor Rheb_P Rheb RSK_P RSK PDK1_P PDK1 MLL_P MLL HOXA9 GBL_P GBL ERK_P ERK AKT_P AKT**
		**miRNA_1976 miRNA_196B mTOR_P mTOR TSC_1_2 Rictor_P Rictor Rheb_P Rheb RSK_P RSK PDK1_P PDK1 MLL_P MLL HOXA9 GBL_P GBL ERK_P ERK AKT_P AKT**
		**miRNA_1976 miRNA_196B mTOR_P mTOR TSC_1_2 Rictor_P Rictor Rheb_P Rheb RSK_P RSK PDK1_P PDK1 MLL_P MLL HOXA9 GBL_P GBL ERK_P ERK AKT_P AKT**
		**miRNA_1976 miRNA_196B mTOR_P mTOR TSC_1_2 Rictor_P Rictor Rheb_P Rheb RSK_P RSK PDK1_P PDK1 MLL_P MLL HOXA9 GBL_P GBL ERK_P ERK AKT_P AKT**
**40**	**40**	Basin Dimension Percentual: **0.03**

		**miRNA_1976 miRNA_196B mTOR_P mTOR TSC_1_2 Rictor_P Rictor Rheb_P Rheb RSK_P RSK PDK1_P PDK1 MLL_P MLL HOXA9 GBL_P GBL ERK_P ERK AKT_P AKT**
		**miRNA_1976 miRNA_196B mTOR_P mTOR TSC_1_2 Rictor_P Rictor Rheb_P Rheb RSK_P RSK PDK1_P PDK1 MLL_P MLL HOXA9 GBL_P GBL ERK_P ERK AKT_P AKT**
		**miRNA_1976 miRNA_196B mTOR_P mTOR TSC_1_2 Rictor_P Rictor Rheb_P Rheb RSK_P RSK PDK1_P PDK1 MLL_P MLL HOXA9 GBL_P GBL ERK_P ERK AKT_P AKT**
		**miRNA_1976 miRNA_196B mTOR_P mTOR TSC_1_2 Rictor_P Rictor Rheb_P Rheb RSK_P RSK PDK1_P PDK1 MLL_P MLL HOXA9 GBL_P GBL ERK_P ERK AKT_P AKT**
		**miRNA_1976 miRNA_196B mTOR_P mTOR TSC_1_2 Rictor_P Rictor Rheb_P Rheb RSK_P RSK PDK1_P PDK1 MLL_P MLL HOXA9 GBL_P GBL ERK_P ERK AKT_P AKT**
		**miRNA_1976 miRNA_196B mTOR_P mTOR TSC_1_2 Rictor_P Rictor Rheb_P Rheb RSK_P RSK PDK1_P PDK1 MLL_P MLL HOXA9 GBL_P GBL ERK_P ERK AKT_P AKT**
		**miRNA_1976 miRNA_196B mTOR_P mTOR TSC_1_2 Rictor_P Rictor Rheb_P Rheb RSK_P RSK PDK1_P PDK1 MLL_P MLL HOXA9 GBL_P GBL ERK_P ERK AKT_P AKT**
**38**	**36**	Basin Dimension Percentual: **0.03**
		**miRNA_1976 miRNA_196B mTOR_P mTOR TSC_1_2 Rictor_P Rictor Rheb_P Rheb RSK_P RSK PDK1_P PDK1 MLL_P MLL HOXA9 GBL_P GBL ERK_P ERK AKT_P AKT**
		**miRNA_1976 miRNA_196B mTOR_P mTOR TSC_1_2 Rictor_P Rictor Rheb_P Rheb RSK_P RSK PDK1_P PDK1 MLL_P MLL HOXA9 GBL_P GBL ERK_P ERK AKT_P AKT**
		**miRNA_1976 miRNA_196B mTOR_P mTOR TSC_1_2 Rictor_P Rictor Rheb_P Rheb RSK_P RSK PDK1_P PDK1 MLL_P MLL HOXA9 GBL_P GBL ERK_P ERK AKT_P AKT**
		**miRNA_1976 miRNA_196B mTOR_P mTOR TSC_1_2 Rictor_P Rictor Rheb_P Rheb RSK_P RSK PDK1_P PDK1 MLL_P MLL HOXA9 GBL_P GBL ERK_P ERK AKT_P AKT**
		**miRNA_1976 miRNA_196B mTOR_P mTOR TSC_1_2 Rictor_P Rictor Rheb_P Rheb RSK_P RSK PDK1_P PDK1 MLL_P MLL HOXA9 GBL_P GBL ERK_P ERK AKT_P AKT**
		**miRNA_1976 miRNA_196B mTOR_P mTOR TSC_1_2 Rictor_P Rictor Rheb_P Rheb RSK_P RSK PDK1_P PDK1 MLL_P MLL**

		**HOXA9 GBL_P GBL ERK_P ERK AKT_P AKT**
		**miRNA_1976 miRNA_196B mTOR_P mTOR TSC_1_2 Rictor_P<br>Rictor Rheb_P Rheb RSK_P RSK PDK1_P PDK1 MLL_P MLL<br>HOXA9 GBL_P GBL ERK_P ERK AKT_P AKT**
**18**	**34**	Basin Dimension Percentual: **0.03**
		**miRNA_1976 miRNA_196B mTOR_P mTOR TSC_1_2 Rictor_P<br>Rictor Rheb_P Rheb RSK_P RSK PDK1_P PDK1 MLL_P MLL<br>HOXA9 GBL_P GBL ERK_P ERK AKT_P AKT**
		**miRNA_1976 miRNA_196B mTOR_P mTOR TSC_1_2 Rictor_P<br>Rictor Rheb_P Rheb RSK_P RSK PDK1_P PDK1 MLL_P MLL<br>HOXA9 GBL_P GBL ERK_P ERK AKT_P AKT**
		**miRNA_1976 miRNA_196B mTOR_P mTOR TSC_1_2 Rictor_P<br>Rictor Rheb_P Rheb RSK_P RSK PDK1_P PDK1 MLL_P MLL<br>HOXA9 GBL_P GBL ERK_P ERK AKT_P AKT**
		**miRNA_1976 miRNA_196B mTOR_P mTOR TSC_1_2 Rictor_P<br>Rictor Rheb_P Rheb RSK_P RSK PDK1_P PDK1 MLL_P MLL<br>HOXA9 GBL_P GBL ERK_P ERK AKT_P AKT**
		**miRNA_1976 miRNA_196B mTOR_P mTOR TSC_1_2 Rictor_P<br>Rictor Rheb_P Rheb RSK_P RSK PDK1_P PDK1 MLL_P MLL<br>HOXA9 GBL_P GBL ERK_P ERK AKT_P AKT**
		**miRNA_1976 miRNA_196B mTOR_P mTOR TSC_1_2 Rictor_P<br>Rictor Rheb_P Rheb RSK_P RSK PDK1_P PDK1 MLL_P MLL<br>HOXA9 GBL_P GBL ERK_P ERK AKT_P AKT**
		**miRNA_1976 miRNA_196B mTOR_P mTOR TSC_1_2 Rictor_P<br>Rictor Rheb_P Rheb RSK_P RSK PDK1_P PDK1 MLL_P MLL<br>HOXA9 GBL_P GBL ERK_P ERK AKT_P AKT**
**39**	**20**	Basin Dimension Percentual: **0.02**
		**miRNA_1976 miRNA_196B mTOR_P mTOR TSC_1_2 Rictor_P<br>Rictor Rheb_P Rheb RSK_P RSK PDK1_P PDK1 MLL_P MLL<br>HOXA9 GBL_P GBL ERK_P ERK AKT_P AKT**
		**miRNA_1976 miRNA_196B mTOR_P mTOR TSC_1_2 Rictor_P<br>Rictor Rheb_P Rheb RSK_P RSK PDK1_P PDK1 MLL_P MLL<br>HOXA9 GBL_P GBL ERK_P ERK AKT_P AKT**
		**miRNA_1976 miRNA_196B mTOR_P mTOR TSC_1_2 Rictor_P<br>Rictor Rheb_P Rheb RSK_P RSK PDK1_P PDK1 MLL_P MLL<br>HOXA9 GBL_P GBL ERK_P ERK AKT_P AKT**
		**miRNA_1976 miRNA_196B mTOR_P mTOR TSC_1_2 Rictor_P<br>Rictor Rheb_P Rheb RSK_P RSK PDK1_P PDK1 MLL_P MLL**

		**HOXA9 GBL_P GBL ERK_P ERK AKT_P AKT**
		**miRNA_1976 miRNA_196B mTOR_P mTOR TSC_1_2 Rictor_P Rictor Rheb_P Rheb RSK_P RSK PDK1_P PDK1 MLL_P MLL HOXA9 GBL_P GBL ERK_P ERK AKT_P AKT**
		**miRNA_1976 miRNA_196B mTOR_P mTOR TSC_1_2 Rictor_P Rictor Rheb_P Rheb RSK_P RSK PDK1_P PDK1 MLL_P MLL HOXA9 GBL_P GBL ERK_P ERK AKT_P AKT**
		**miRNA_1976 miRNA_196B mTOR_P mTOR TSC_1_2 Rictor_P Rictor Rheb_P Rheb RSK_P RSK PDK1_P PDK1 MLL_P MLL HOXA9 GBL_P GBL ERK_P ERK AKT_P AKT**
**37**	**18**	Basin Dimension Percentual: **0.01**
		**miRNA_1976 miRNA_196B mTOR_P mTOR TSC_1_2 Rictor_P Rictor Rheb_P Rheb RSK_P RSK PDK1_P PDK1 MLL_P MLL HOXA9 GBL_P GBL ERK_P ERK AKT_P AKT**
		**miRNA_1976 miRNA_196B mTOR_P mTOR TSC_1_2 Rictor_P Rictor Rheb_P Rheb RSK_P RSK PDK1_P PDK1 MLL_P MLL HOXA9 GBL_P GBL ERK_P ERK AKT_P AKT**
		**miRNA_1976 miRNA_196B mTOR_P mTOR TSC_1_2 Rictor_P Rictor Rheb_P Rheb RSK_P RSK PDK1_P PDK1 MLL_P MLL HOXA9 GBL_P GBL ERK_P ERK AKT_P AKT**
		**miRNA_1976 miRNA_196B mTOR_P mTOR TSC_1_2 Rictor_P Rictor Rheb_P Rheb RSK_P RSK PDK1_P PDK1 MLL_P MLL HOXA9 GBL_P GBL ERK_P ERK AKT_P AKT**
		**miRNA_1976 miRNA_196B mTOR_P mTOR TSC_1_2 Rictor_P Rictor Rheb_P Rheb RSK_P RSK PDK1_P PDK1 MLL_P MLL HOXA9 GBL_P GBL ERK_P ERK AKT_P AKT**
		**miRNA_1976 miRNA_196B mTOR_P mTOR TSC_1_2 Rictor_P Rictor Rheb_P Rheb RSK_P RSK PDK1_P PDK1 MLL_P MLL HOXA9 GBL_P GBL ERK_P ERK AKT_P AKT**
		**miRNA_1976 miRNA_196B mTOR_P mTOR TSC_1_2 Rictor_P Rictor Rheb_P Rheb RSK_P RSK PDK1_P PDK1 MLL_P MLL HOXA9 GBL_P GBL ERK_P ERK AKT_P AKT**
**15**	**17**	Basin Dimension Percentual: **0.01**
		**miRNA_1976 miRNA_196B mTOR_P mTOR TSC_1_2 Rictor_P Rictor Rheb_P Rheb RSK_P RSK PDK1_P PDK1 MLL_P MLL HOXA9 GBL_P GBL ERK_P ERK AKT_P AKT**
		**miRNA_1976 miRNA_196B mTOR_P mTOR TSC_1_2 Rictor_P Rictor Rheb_P Rheb RSK_P RSK PDK1_P PDK1 MLL_P MLL**

		**HOXA9 GBL_P GBL ERK_P ERK AKT_P AKT**
		**miRNA_1976 miRNA_196B mTOR_P mTOR TSC_1_2 Rictor_P Rictor Rheb_P Rheb RSK_P RSK PDK1_P PDK1 MLL_P MLL HOXA9 GBL_P GBL ERK_P ERK AKT_P AKT**
		**miRNA_1976 miRNA_196B mTOR_P mTOR TSC_1_2 Rictor_P Rictor Rheb_P Rheb RSK_P RSK PDK1_P PDK1 MLL_P MLL HOXA9 GBL_P GBL ERK_P ERK AKT_P AKT**
		**miRNA_1976 miRNA_196B mTOR_P mTOR TSC_1_2 Rictor_P Rictor Rheb_P Rheb RSK_P RSK PDK1_P PDK1 MLL_P MLL HOXA9 GBL_P GBL ERK_P ERK AKT_P AKT**
		**miRNA_1976 miRNA_196B mTOR_P mTOR TSC_1_2 Rictor_P Rictor Rheb_P Rheb RSK_P RSK PDK1_P PDK1 MLL_P MLL HOXA9 GBL_P GBL ERK_P ERK AKT_P AKT**
		**miRNA_1976 miRNA_196B mTOR_P mTOR TSC_1_2 Rictor_P Rictor Rheb_P Rheb RSK_P RSK PDK1_P PDK1 MLL_P MLL HOXA9 GBL_P GBL ERK_P ERK AKT_P AKT**
**89**	**4**	Basin Dimension Percentual: **0.00**
		**miRNA_1976 miRNA_196B mTOR_P mTOR TSC_1_2 Rictor_P Rictor Rheb_P Rheb RSK_P RSK PDK1_P PDK1 MLL_P MLL HOXA9 GBL_P GBL ERK_P ERK AKT_P AKT**
**42**	**2**	Basin Dimension Percentual: **0.00**
		**miRNA_1976 miRNA_196B mTOR_P mTOR TSC_1_2 Rictor_P Rictor Rheb_P Rheb RSK_P RSK PDK1_P PDK1 MLL_P MLL HOXA9 GBL_P GBL ERK_P ERK AKT_P AKT**
**91**	**2**	Basin Dimension Percentual: **0.00**
		**miRNA_1976 miRNA_196B mTOR_P mTOR TSC_1_2 Rictor_P Rictor Rheb_P Rheb RSK_P RSK PDK1_P PDK1 MLL_P MLL HOXA9 GBL_P GBL ERK_P ERK AKT_P AKT**
**41**	**1**	Basin Dimension Percentual: **0.00**
		**miRNA_1976 miRNA_196B mTOR_P mTOR TSC_1_2 Rictor_P Rictor Rheb_P Rheb RSK_P RSK PDK1_P PDK1 MLL_P MLL HOXA9 GBL_P GBL ERK_P ERK AKT_P AKT**
