# Supplementary material for: An extended gene protein/products boolean network model including post-transcriptional regulation
Source: Theor Biol Med Model. 2014 May 7;11(Suppl 1):S5. doi: 10.1186/1742-4682-11-S1-S5 (PMC4108923; doi:10.1186/1742-4682-11-S1-S5)
Supplement: Additional File 2 — contains the whole list of attractors for the mTOR network with deleted edge between miR-1976 and MLL [file 1742-4682-11-S1-S5-S2.PDF]

Attractors Search Simulation

Description:

mTOR exhaustive attractors search (Faulty network)

Attractor Number	Hits	Data
**73**	**41472**	Basin Dimension Percentual: **41.61**
		**miRNA_1976 miRNA_196B mTOR_P mTOR TSC_1_2 Rictor_P Rictor Rheb_P Rheb RSK_P RSK PDK1_P PDK1 MLL_P MLL HOXA9 GBL_P GBL ERK_P ERK AKT_P AKT**
**69**	**20736**	Basin Dimension Percentual: **20.80**
		**miRNA_1976 miRNA_196B mTOR_P mTOR TSC_1_2 Rictor_P Rictor Rheb_P Rheb RSK_P RSK PDK1_P PDK1 MLL_P MLL HOXA9 GBL_P GBL ERK_P ERK AKT_P AKT**
**71**	**20736**	Basin Dimension Percentual: **20.80**
		**miRNA_1976 miRNA_196B mTOR_P mTOR TSC_1_2 Rictor_P Rictor Rheb_P Rheb RSK_P RSK PDK1_P PDK1 MLL_P MLL HOXA9 GBL_P GBL ERK_P ERK AKT_P AKT**
**72**	**20736**	Basin Dimension Percentual: **20.80**
		**miRNA_1976 miRNA_196B mTOR_P mTOR TSC_1_2 Rictor_P Rictor Rheb_P Rheb RSK_P RSK PDK1_P PDK1 MLL_P MLL HOXA9 GBL_P GBL ERK_P ERK AKT_P AKT**
**46**	**10368**	Basin Dimension Percentual: **10.40**
		**miRNA_1976 miRNA_196B mTOR_P mTOR TSC_1_2 Rictor_P Rictor Rheb_P Rheb RSK_P RSK PDK1_P PDK1 MLL_P MLL HOXA9 GBL_P GBL ERK_P ERK AKT_P AKT**
**67**	**10368**	Basin Dimension Percentual: **10.40**
		**miRNA_1976 miRNA_196B mTOR_P mTOR TSC_1_2 Rictor_P Rictor Rheb_P Rheb RSK_P RSK PDK1_P PDK1 MLL_P MLL HOXA9 GBL_P GBL ERK_P ERK AKT_P AKT**
**70**	**10368**	Basin Dimension Percentual: **10.40**
		**miRNA_1976 miRNA_196B mTOR_P mTOR TSC_1_2 Rictor_P Rictor Rheb_P Rheb RSK_P RSK PDK1_P PDK1 MLL_P MLL HOXA9 GBL_P GBL ERK_P ERK AKT_P AKT**

**44**	**5184**	Basin Dimension Percentual: **5.20**
		**miRNA_1976 miRNA_196B mTOR_P mTOR TSC_1_2 Rictor_P Rictor Rheb_P Rheb RSK_P RSK PDK1_P PDK1 MLL_P MLL HOXA9 GBL_P GBL ERK_P ERK AKT_P AKT**
**45**	**5184**	Basin Dimension Percentual: **5.20**
		**miRNA_1976 miRNA_196B mTOR_P mTOR TSC_1_2 Rictor_P Rictor Rheb_P Rheb RSK_P RSK PDK1_P PDK1 MLL_P MLL HOXA9 GBL_P GBL ERK_P ERK AKT_P AKT**
**58**	**5184**	Basin Dimension Percentual: **5.20**
		**miRNA_1976 miRNA_196B mTOR_P mTOR TSC_1_2 Rictor_P Rictor Rheb_P Rheb RSK_P RSK PDK1_P PDK1 MLL_P MLL HOXA9 GBL_P GBL ERK_P ERK AKT_P AKT**
**24**	**2592**	Basin Dimension Percentual: **2.60**
		**miRNA_1976 miRNA_196B mTOR_P mTOR TSC_1_2 Rictor_P Rictor Rheb_P Rheb RSK_P RSK PDK1_P PDK1 MLL_P MLL HOXA9 GBL_P GBL ERK_P ERK AKT_P AKT**
**43**	**2592**	Basin Dimension Percentual: **2.60**
		**miRNA_1976 miRNA_196B mTOR_P mTOR TSC_1_2 Rictor_P Rictor Rheb_P Rheb RSK_P RSK PDK1_P PDK1 MLL_P MLL HOXA9 GBL_P GBL ERK_P ERK AKT_P AKT**
**47**	**1760**	Basin Dimension Percentual: **1.77**
		**miRNA_1976 miRNA_196B mTOR_P mTOR TSC_1_2 Rictor_P Rictor Rheb_P Rheb RSK_P RSK PDK1_P PDK1 MLL_P MLL HOXA9 GBL_P GBL ERK_P ERK AKT_P AKT**
		**miRNA_1976 miRNA_196B mTOR_P mTOR TSC_1_2 Rictor_P Rictor Rheb_P Rheb RSK_P RSK PDK1_P PDK1 MLL_P MLL HOXA9 GBL_P GBL ERK_P ERK AKT_P AKT**
		**miRNA_1976 miRNA_196B mTOR_P mTOR TSC_1_2 Rictor_P Rictor Rheb_P Rheb RSK_P RSK PDK1_P PDK1 MLL_P MLL HOXA9 GBL_P GBL ERK_P ERK AKT_P AKT**
		**miRNA_1976 miRNA_196B mTOR_P mTOR TSC_1_2 Rictor_P Rictor Rheb_P Rheb RSK_P RSK PDK1_P PDK1 MLL_P MLL HOXA9 GBL_P GBL ERK_P ERK AKT_P AKT**
		**miRNA_1976 miRNA_196B mTOR_P mTOR TSC_1_2 Rictor_P Rictor Rheb_P Rheb RSK_P RSK PDK1_P PDK1 MLL_P MLL HOXA9 GBL_P GBL ERK_P ERK AKT_P AKT**

		**miRNA_1976 miRNA_196B mTOR_P mTOR TSC_1_2 Rictor_P Rictor Rheb_P Rheb RSK_P RSK PDK1_P PDK1 MLL_P MLL HOXA9 GBL_P GBL ERK_P ERK AKT_P AKT**
		**miRNA_1976 miRNA_196B mTOR_P mTOR TSC_1_2 Rictor_P Rictor Rheb_P Rheb RSK_P RSK PDK1_P PDK1 MLL_P MLL HOXA9 GBL_P GBL ERK_P ERK AKT_P AKT**
**49**	**1352**	Basin Dimension Percentual: **1.36**
		**miRNA_1976 miRNA_196B mTOR_P mTOR TSC_1_2 Rictor_P Rictor Rheb_P Rheb RSK_P RSK PDK1_P PDK1 MLL_P MLL HOXA9 GBL_P GBL ERK_P ERK AKT_P AKT**
		**miRNA_1976 miRNA_196B mTOR_P mTOR TSC_1_2 Rictor_P Rictor Rheb_P Rheb RSK_P RSK PDK1_P PDK1 MLL_P MLL HOXA9 GBL_P GBL ERK_P ERK AKT_P AKT**
		**miRNA_1976 miRNA_196B mTOR_P mTOR TSC_1_2 Rictor_P Rictor Rheb_P Rheb RSK_P RSK PDK1_P PDK1 MLL_P MLL HOXA9 GBL_P GBL ERK_P ERK AKT_P AKT**
		**miRNA_1976 miRNA_196B mTOR_P mTOR TSC_1_2 Rictor_P Rictor Rheb_P Rheb RSK_P RSK PDK1_P PDK1 MLL_P MLL HOXA9 GBL_P GBL ERK_P ERK AKT_P AKT**
		**miRNA_1976 miRNA_196B mTOR_P mTOR TSC_1_2 Rictor_P Rictor Rheb_P Rheb RSK_P RSK PDK1_P PDK1 MLL_P MLL HOXA9 GBL_P GBL ERK_P ERK AKT_P AKT**
		**miRNA_1976 miRNA_196B mTOR_P mTOR TSC_1_2 Rictor_P Rictor Rheb_P Rheb RSK_P RSK PDK1_P PDK1 MLL_P MLL HOXA9 GBL_P GBL ERK_P ERK AKT_P AKT**
		**miRNA_1976 miRNA_196B mTOR_P mTOR TSC_1_2 Rictor_P Rictor Rheb_P Rheb RSK_P RSK PDK1_P PDK1 MLL_P MLL HOXA9 GBL_P GBL ERK_P ERK AKT_P AKT**
**23**	**1296**	Basin Dimension Percentual: **1.30**
		**miRNA_1976 miRNA_196B mTOR_P mTOR TSC_1_2 Rictor_P Rictor Rheb_P Rheb RSK_P RSK PDK1_P PDK1 MLL_P MLL HOXA9 GBL_P GBL ERK_P ERK AKT_P AKT**
**54**	**924**	Basin Dimension Percentual: **0.93**
		**miRNA_1976 miRNA_196B mTOR_P mTOR TSC_1_2 Rictor_P Rictor Rheb_P Rheb RSK_P RSK PDK1_P PDK1 MLL_P MLL HOXA9 GBL_P GBL ERK_P ERK AKT_P AKT**
		**miRNA_1976 miRNA_196B mTOR_P mTOR TSC_1_2 Rictor_P Rictor Rheb_P Rheb RSK_P RSK PDK1_P PDK1 MLL_P MLL**

		**HOXA9 GBL_P GBL ERK_P ERK AKT_P AKT**
		**miRNA_1976 miRNA_196B mTOR_P mTOR TSC_1_2 Rictor_P Rictor Rheb_P Rheb RSK_P RSK PDK1_P PDK1 MLL_P MLL HOXA9 GBL_P GBL ERK_P ERK AKT_P AKT**
		**miRNA_1976 miRNA_196B mTOR_P mTOR TSC_1_2 Rictor_P Rictor Rheb_P Rheb RSK_P RSK PDK1_P PDK1 MLL_P MLL HOXA9 GBL_P GBL ERK_P ERK AKT_P AKT**
		**miRNA_1976 miRNA_196B mTOR_P mTOR TSC_1_2 Rictor_P Rictor Rheb_P Rheb RSK_P RSK PDK1_P PDK1 MLL_P MLL HOXA9 GBL_P GBL ERK_P ERK AKT_P AKT**
		**miRNA_1976 miRNA_196B mTOR_P mTOR TSC_1_2 Rictor_P Rictor Rheb_P Rheb RSK_P RSK PDK1_P PDK1 MLL_P MLL HOXA9 GBL_P GBL ERK_P ERK AKT_P AKT**
		**miRNA_1976 miRNA_196B mTOR_P mTOR TSC_1_2 Rictor_P Rictor Rheb_P Rheb RSK_P RSK PDK1_P PDK1 MLL_P MLL HOXA9 GBL_P GBL ERK_P ERK AKT_P AKT**
**5**	**880**	Basin Dimension Percentual: **0.88**
		**miRNA_1976 miRNA_196B mTOR_P mTOR TSC_1_2 Rictor_P Rictor Rheb_P Rheb RSK_P RSK PDK1_P PDK1 MLL_P MLL HOXA9 GBL_P GBL ERK_P ERK AKT_P AKT**
		**miRNA_1976 miRNA_196B mTOR_P mTOR TSC_1_2 Rictor_P Rictor Rheb_P Rheb RSK_P RSK PDK1_P PDK1 MLL_P MLL HOXA9 GBL_P GBL ERK_P ERK AKT_P AKT**
		**miRNA_1976 miRNA_196B mTOR_P mTOR TSC_1_2 Rictor_P Rictor Rheb_P Rheb RSK_P RSK PDK1_P PDK1 MLL_P MLL HOXA9 GBL_P GBL ERK_P ERK AKT_P AKT**
		**miRNA_1976 miRNA_196B mTOR_P mTOR TSC_1_2 Rictor_P Rictor Rheb_P Rheb RSK_P RSK PDK1_P PDK1 MLL_P MLL HOXA9 GBL_P GBL ERK_P ERK AKT_P AKT**
		**miRNA_1976 miRNA_196B mTOR_P mTOR TSC_1_2 Rictor_P Rictor Rheb_P Rheb RSK_P RSK PDK1_P PDK1 MLL_P MLL HOXA9 GBL_P GBL ERK_P ERK AKT_P AKT**
		**miRNA_1976 miRNA_196B mTOR_P mTOR TSC_1_2 Rictor_P Rictor Rheb_P Rheb RSK_P RSK PDK1_P PDK1 MLL_P MLL HOXA9 GBL_P GBL ERK_P ERK AKT_P AKT**
		**miRNA_1976 miRNA_196B mTOR_P mTOR TSC_1_2 Rictor_P Rictor Rheb_P Rheb RSK_P RSK PDK1_P PDK1 MLL_P MLL HOXA9 GBL_P GBL ERK_P ERK AKT_P AKT**

**51**	**876**	Basin Dimension Percentual: **0.88**
		**miRNA_1976 miRNA_196B mTOR_P mTOR TSC_1_2 Rictor_P Rictor Rheb_P Rheb RSK_P RSK PDK1_P PDK1 MLL_P MLL HOXA9 GBL_P GBL ERK_P ERK AKT_P AKT**
		**miRNA_1976 miRNA_196B mTOR_P mTOR TSC_1_2 Rictor_P Rictor Rheb_P Rheb RSK_P RSK PDK1_P PDK1 MLL_P MLL HOXA9 GBL_P GBL ERK_P ERK AKT_P AKT**
		**miRNA_1976 miRNA_196B mTOR_P mTOR TSC_1_2 Rictor_P Rictor Rheb_P Rheb RSK_P RSK PDK1_P PDK1 MLL_P MLL HOXA9 GBL_P GBL ERK_P ERK AKT_P AKT**
		**miRNA_1976 miRNA_196B mTOR_P mTOR TSC_1_2 Rictor_P Rictor Rheb_P Rheb RSK_P RSK PDK1_P PDK1 MLL_P MLL HOXA9 GBL_P GBL ERK_P ERK AKT_P AKT**
		**miRNA_1976 miRNA_196B mTOR_P mTOR TSC_1_2 Rictor_P Rictor Rheb_P Rheb RSK_P RSK PDK1_P PDK1 MLL_P MLL HOXA9 GBL_P GBL ERK_P ERK AKT_P AKT**
		**miRNA_1976 miRNA_196B mTOR_P mTOR TSC_1_2 Rictor_P Rictor Rheb_P Rheb RSK_P RSK PDK1_P PDK1 MLL_P MLL HOXA9 GBL_P GBL ERK_P ERK AKT_P AKT**
		**miRNA_1976 miRNA_196B mTOR_P mTOR TSC_1_2 Rictor_P Rictor Rheb_P Rheb RSK_P RSK PDK1_P PDK1 MLL_P MLL HOXA9 GBL_P GBL ERK_P ERK AKT_P AKT**
**60**	**864**	Basin Dimension Percentual: **0.87**
		**miRNA_1976 miRNA_196B mTOR_P mTOR TSC_1_2 Rictor_P Rictor Rheb_P Rheb RSK_P RSK PDK1_P PDK1 MLL_P MLL HOXA9 GBL_P GBL ERK_P ERK AKT_P AKT**
		**miRNA_1976 miRNA_196B mTOR_P mTOR TSC_1_2 Rictor_P Rictor Rheb_P Rheb RSK_P RSK PDK1_P PDK1 MLL_P MLL HOXA9 GBL_P GBL ERK_P ERK AKT_P AKT**
		**miRNA_1976 miRNA_196B mTOR_P mTOR TSC_1_2 Rictor_P Rictor Rheb_P Rheb RSK_P RSK PDK1_P PDK1 MLL_P MLL HOXA9 GBL_P GBL ERK_P ERK AKT_P AKT**
		**miRNA_1976 miRNA_196B mTOR_P mTOR TSC_1_2 Rictor_P Rictor Rheb_P Rheb RSK_P RSK PDK1_P PDK1 MLL_P MLL HOXA9 GBL_P GBL ERK_P ERK AKT_P AKT**
		**miRNA_1976 miRNA_196B mTOR_P mTOR TSC_1_2 Rictor_P Rictor Rheb_P Rheb RSK_P RSK PDK1_P PDK1 MLL_P MLL HOXA9 GBL_P GBL ERK_P ERK AKT_P AKT**

		**miRNA_1976 miRNA_196B mTOR_P mTOR TSC_1_2 Rictor_P Rictor Rheb_P Rheb RSK_P RSK PDK1_P PDK1 MLL_P MLL HOXA9 GBL_P GBL ERK_P ERK AKT_P AKT**
		**miRNA_1976 miRNA_196B mTOR_P mTOR TSC_1_2 Rictor_P Rictor Rheb_P Rheb RSK_P RSK PDK1_P PDK1 MLL_P MLL HOXA9 GBL_P GBL ERK_P ERK AKT_P AKT**
**52**	**816**	Basin Dimension Percentual: **0.82**
		**miRNA_1976 miRNA_196B mTOR_P mTOR TSC_1_2 Rictor_P Rictor Rheb_P Rheb RSK_P RSK PDK1_P PDK1 MLL_P MLL HOXA9 GBL_P GBL ERK_P ERK AKT_P AKT**
		**miRNA_1976 miRNA_196B mTOR_P mTOR TSC_1_2 Rictor_P Rictor Rheb_P Rheb RSK_P RSK PDK1_P PDK1 MLL_P MLL HOXA9 GBL_P GBL ERK_P ERK AKT_P AKT**
		**miRNA_1976 miRNA_196B mTOR_P mTOR TSC_1_2 Rictor_P Rictor Rheb_P Rheb RSK_P RSK PDK1_P PDK1 MLL_P MLL HOXA9 GBL_P GBL ERK_P ERK AKT_P AKT**
		**miRNA_1976 miRNA_196B mTOR_P mTOR TSC_1_2 Rictor_P Rictor Rheb_P Rheb RSK_P RSK PDK1_P PDK1 MLL_P MLL HOXA9 GBL_P GBL ERK_P ERK AKT_P AKT**
		**miRNA_1976 miRNA_196B mTOR_P mTOR TSC_1_2 Rictor_P Rictor Rheb_P Rheb RSK_P RSK PDK1_P PDK1 MLL_P MLL HOXA9 GBL_P GBL ERK_P ERK AKT_P AKT**
		**miRNA_1976 miRNA_196B mTOR_P mTOR TSC_1_2 Rictor_P Rictor Rheb_P Rheb RSK_P RSK PDK1_P PDK1 MLL_P MLL HOXA9 GBL_P GBL ERK_P ERK AKT_P AKT**
		**miRNA_1976 miRNA_196B mTOR_P mTOR TSC_1_2 Rictor_P Rictor Rheb_P Rheb RSK_P RSK PDK1_P PDK1 MLL_P MLL HOXA9 GBL_P GBL ERK_P ERK AKT_P AKT**
**48**	**748**	Basin Dimension Percentual: **0.75**
		**miRNA_1976 miRNA_196B mTOR_P mTOR TSC_1_2 Rictor_P Rictor Rheb_P Rheb RSK_P RSK PDK1_P PDK1 MLL_P MLL HOXA9 GBL_P GBL ERK_P ERK AKT_P AKT**
		**miRNA_1976 miRNA_196B mTOR_P mTOR TSC_1_2 Rictor_P Rictor Rheb_P Rheb RSK_P RSK PDK1_P PDK1 MLL_P MLL HOXA9 GBL_P GBL ERK_P ERK AKT_P AKT**
		**miRNA_1976 miRNA_196B mTOR_P mTOR TSC_1_2 Rictor_P Rictor Rheb_P Rheb RSK_P RSK PDK1_P PDK1 MLL_P MLL HOXA9 GBL_P GBL ERK_P ERK AKT_P AKT**

		**miRNA_1976 miRNA_196B mTOR_P mTOR TSC_1_2 Rictor_P Rictor Rheb_P Rheb RSK_P RSK PDK1_P PDK1 MLL_P MLL HOXA9 GBL_P GBL ERK_P ERK AKT_P AKT**
		**miRNA_1976 miRNA_196B mTOR_P mTOR TSC_1_2 Rictor_P Rictor Rheb_P Rheb RSK_P RSK PDK1_P PDK1 MLL_P MLL HOXA9 GBL_P GBL ERK_P ERK AKT_P AKT**
		**miRNA_1976 miRNA_196B mTOR_P mTOR TSC_1_2 Rictor_P Rictor Rheb_P Rheb RSK_P RSK PDK1_P PDK1 MLL_P MLL HOXA9 GBL_P GBL ERK_P ERK AKT_P AKT**
		**miRNA_1976 miRNA_196B mTOR_P mTOR TSC_1_2 Rictor_P Rictor Rheb_P Rheb RSK_P RSK PDK1_P PDK1 MLL_P MLL HOXA9 GBL_P GBL ERK_P ERK AKT_P AKT**
**7**	**676**	Basin Dimension Percentual: **0.68**
		**miRNA_1976 miRNA_196B mTOR_P mTOR TSC_1_2 Rictor_P Rictor Rheb_P Rheb RSK_P RSK PDK1_P PDK1 MLL_P MLL HOXA9 GBL_P GBL ERK_P ERK AKT_P AKT**
		**miRNA_1976 miRNA_196B mTOR_P mTOR TSC_1_2 Rictor_P Rictor Rheb_P Rheb RSK_P RSK PDK1_P PDK1 MLL_P MLL HOXA9 GBL_P GBL ERK_P ERK AKT_P AKT**
		**miRNA_1976 miRNA_196B mTOR_P mTOR TSC_1_2 Rictor_P Rictor Rheb_P Rheb RSK_P RSK PDK1_P PDK1 MLL_P MLL HOXA9 GBL_P GBL ERK_P ERK AKT_P AKT**
		**miRNA_1976 miRNA_196B mTOR_P mTOR TSC_1_2 Rictor_P Rictor Rheb_P Rheb RSK_P RSK PDK1_P PDK1 MLL_P MLL HOXA9 GBL_P GBL ERK_P ERK AKT_P AKT**
		**miRNA_1976 miRNA_196B mTOR_P mTOR TSC_1_2 Rictor_P Rictor Rheb_P Rheb RSK_P RSK PDK1_P PDK1 MLL_P MLL HOXA9 GBL_P GBL ERK_P ERK AKT_P AKT**
		**miRNA_1976 miRNA_196B mTOR_P mTOR TSC_1_2 Rictor_P Rictor Rheb_P Rheb RSK_P RSK PDK1_P PDK1 MLL_P MLL HOXA9 GBL_P GBL ERK_P ERK AKT_P AKT**
		**miRNA_1976 miRNA_196B mTOR_P mTOR TSC_1_2 Rictor_P Rictor Rheb_P Rheb RSK_P RSK PDK1_P PDK1 MLL_P MLL HOXA9 GBL_P GBL ERK_P ERK AKT_P AKT**
**50**	**668**	Basin Dimension Percentual: **0.67**
		**miRNA_1976 miRNA_196B mTOR_P mTOR TSC_1_2 Rictor_P Rictor Rheb_P Rheb RSK_P RSK PDK1_P PDK1 MLL_P MLL HOXA9 GBL_P GBL ERK_P ERK AKT_P AKT**

		**miRNA_1976 miRNA_196B mTOR_P mTOR TSC_1_2 Rictor_P Rictor Rheb_P Rheb RSK_P RSK PDK1_P PDK1 MLL_P MLL HOXA9 GBL_P GBL ERK_P ERK AKT_P AKT**
		**miRNA_1976 miRNA_196B mTOR_P mTOR TSC_1_2 Rictor_P Rictor Rheb_P Rheb RSK_P RSK PDK1_P PDK1 MLL_P MLL HOXA9 GBL_P GBL ERK_P ERK AKT_P AKT**
		**miRNA_1976 miRNA_196B mTOR_P mTOR TSC_1_2 Rictor_P Rictor Rheb_P Rheb RSK_P RSK PDK1_P PDK1 MLL_P MLL HOXA9 GBL_P GBL ERK_P ERK AKT_P AKT**
		**miRNA_1976 miRNA_196B mTOR_P mTOR TSC_1_2 Rictor_P Rictor Rheb_P Rheb RSK_P RSK PDK1_P PDK1 MLL_P MLL HOXA9 GBL_P GBL ERK_P ERK AKT_P AKT**
		**miRNA_1976 miRNA_196B mTOR_P mTOR TSC_1_2 Rictor_P Rictor Rheb_P Rheb RSK_P RSK PDK1_P PDK1 MLL_P MLL HOXA9 GBL_P GBL ERK_P ERK AKT_P AKT**
		**miRNA_1976 miRNA_196B mTOR_P mTOR TSC_1_2 Rictor_P Rictor Rheb_P Rheb RSK_P RSK PDK1_P PDK1 MLL_P MLL HOXA9 GBL_P GBL ERK_P ERK AKT_P AKT**
**17**	**462**	Basin Dimension Percentual: **0.46**
		**miRNA_1976 miRNA_196B mTOR_P mTOR TSC_1_2 Rictor_P Rictor Rheb_P Rheb RSK_P RSK PDK1_P PDK1 MLL_P MLL HOXA9 GBL_P GBL ERK_P ERK AKT_P AKT**
		**miRNA_1976 miRNA_196B mTOR_P mTOR TSC_1_2 Rictor_P Rictor Rheb_P Rheb RSK_P RSK PDK1_P PDK1 MLL_P MLL HOXA9 GBL_P GBL ERK_P ERK AKT_P AKT**
		**miRNA_1976 miRNA_196B mTOR_P mTOR TSC_1_2 Rictor_P Rictor Rheb_P Rheb RSK_P RSK PDK1_P PDK1 MLL_P MLL HOXA9 GBL_P GBL ERK_P ERK AKT_P AKT**
		**miRNA_1976 miRNA_196B mTOR_P mTOR TSC_1_2 Rictor_P Rictor Rheb_P Rheb RSK_P RSK PDK1_P PDK1 MLL_P MLL HOXA9 GBL_P GBL ERK_P ERK AKT_P AKT**
		**miRNA_1976 miRNA_196B mTOR_P mTOR TSC_1_2 Rictor_P Rictor Rheb_P Rheb RSK_P RSK PDK1_P PDK1 MLL_P MLL HOXA9 GBL_P GBL ERK_P ERK AKT_P AKT**
		**miRNA_1976 miRNA_196B mTOR_P mTOR TSC_1_2 Rictor_P Rictor Rheb_P Rheb RSK_P RSK PDK1_P PDK1 MLL_P MLL HOXA9 GBL_P GBL ERK_P ERK AKT_P AKT**
		**miRNA_1976 miRNA_196B mTOR_P mTOR TSC_1_2 Rictor_P Rictor Rheb_P Rheb RSK_P RSK PDK1_P PDK1 MLL_P MLL**

		**HOXA9 GBL_P GBL ERK_P ERK AKT_P AKT**
**59**	**448**	Basin Dimension Percentual: **0.45**
		**miRNA_1976 miRNA_196B mTOR_P mTOR TSC_1_2 Rictor_P Rictor Rheb_P Rheb RSK_P RSK PDK1_P PDK1 MLL_P MLL HOXA9 GBL_P GBL ERK_P ERK AKT_P AKT**
**1**	**440**	Basin Dimension Percentual: **0.44**
		**miRNA_1976 miRNA_196B mTOR_P mTOR TSC_1_2 Rictor_P Rictor Rheb_P Rheb RSK_P RSK PDK1_P PDK1 MLL_P MLL HOXA9 GBL_P GBL ERK_P ERK AKT_P AKT**
		**miRNA_1976 miRNA_196B mTOR_P mTOR TSC_1_2 Rictor_P Rictor Rheb_P Rheb RSK_P RSK PDK1_P PDK1 MLL_P MLL HOXA9 GBL_P GBL ERK_P ERK AKT_P AKT**
		**miRNA_1976 miRNA_196B mTOR_P mTOR TSC_1_2 Rictor_P Rictor Rheb_P Rheb RSK_P RSK PDK1_P PDK1 MLL_P MLL HOXA9 GBL_P GBL ERK_P ERK AKT_P AKT**
		**miRNA_1976 miRNA_196B mTOR_P mTOR TSC_1_2 Rictor_P Rictor Rheb_P Rheb RSK_P RSK PDK1_P PDK1 MLL_P MLL HOXA9 GBL_P GBL ERK_P ERK AKT_P AKT**
		**miRNA_1976 miRNA_196B mTOR_P mTOR TSC_1_2 Rictor_P Rictor Rheb_P Rheb RSK_P RSK PDK1_P PDK1 MLL_P MLL HOXA9 GBL_P GBL ERK_P ERK AKT_P AKT**
		**miRNA_1976 miRNA_196B mTOR_P mTOR TSC_1_2 Rictor_P Rictor Rheb_P Rheb RSK_P RSK PDK1_P PDK1 MLL_P MLL HOXA9 GBL_P GBL ERK_P ERK AKT_P AKT**
		**miRNA_1976 miRNA_196B mTOR_P mTOR TSC_1_2 Rictor_P Rictor Rheb_P Rheb RSK_P RSK PDK1_P PDK1 MLL_P MLL HOXA9 GBL_P GBL ERK_P ERK AKT_P AKT**
**11**	**438**	Basin Dimension Percentual: **0.44**
		**miRNA_1976 miRNA_196B mTOR_P mTOR TSC_1_2 Rictor_P Rictor Rheb_P Rheb RSK_P RSK PDK1_P PDK1 MLL_P MLL HOXA9 GBL_P GBL ERK_P ERK AKT_P AKT**
		**miRNA_1976 miRNA_196B mTOR_P mTOR TSC_1_2 Rictor_P Rictor Rheb_P Rheb RSK_P RSK PDK1_P PDK1 MLL_P MLL HOXA9 GBL_P GBL ERK_P ERK AKT_P AKT**
		**miRNA_1976 miRNA_196B mTOR_P mTOR TSC_1_2 Rictor_P Rictor Rheb_P Rheb RSK_P RSK PDK1_P PDK1 MLL_P MLL HOXA9 GBL_P GBL ERK_P ERK AKT_P AKT**

		**miRNA_1976 miRNA_196B mTOR_P mTOR TSC_1_2 Rictor_P Rictor Rheb_P Rheb RSK_P RSK PDK1_P PDK1 MLL_P MLL HOXA9 GBL_P GBL ERK_P ERK AKT_P AKT**
		**miRNA_1976 miRNA_196B mTOR_P mTOR TSC_1_2 Rictor_P Rictor Rheb_P Rheb RSK_P RSK PDK1_P PDK1 MLL_P MLL HOXA9 GBL_P GBL ERK_P ERK AKT_P AKT**
		**miRNA_1976 miRNA_196B mTOR_P mTOR TSC_1_2 Rictor_P Rictor Rheb_P Rheb RSK_P RSK PDK1_P PDK1 MLL_P MLL HOXA9 GBL_P GBL ERK_P ERK AKT_P AKT**
		**miRNA_1976 miRNA_196B mTOR_P mTOR TSC_1_2 Rictor_P Rictor Rheb_P Rheb RSK_P RSK PDK1_P PDK1 MLL_P MLL HOXA9 GBL_P GBL ERK_P ERK AKT_P AKT**
**28**	**432**	Basin Dimension Percentual: **0.43**
		**miRNA_1976 miRNA_196B mTOR_P mTOR TSC_1_2 Rictor_P Rictor Rheb_P Rheb RSK_P RSK PDK1_P PDK1 MLL_P MLL HOXA9 GBL_P GBL ERK_P ERK AKT_P AKT**
		**miRNA_1976 miRNA_196B mTOR_P mTOR TSC_1_2 Rictor_P Rictor Rheb_P Rheb RSK_P RSK PDK1_P PDK1 MLL_P MLL HOXA9 GBL_P GBL ERK_P ERK AKT_P AKT**
		**miRNA_1976 miRNA_196B mTOR_P mTOR TSC_1_2 Rictor_P Rictor Rheb_P Rheb RSK_P RSK PDK1_P PDK1 MLL_P MLL HOXA9 GBL_P GBL ERK_P ERK AKT_P AKT**
		**miRNA_1976 miRNA_196B mTOR_P mTOR TSC_1_2 Rictor_P Rictor Rheb_P Rheb RSK_P RSK PDK1_P PDK1 MLL_P MLL HOXA9 GBL_P GBL ERK_P ERK AKT_P AKT**
		**miRNA_1976 miRNA_196B mTOR_P mTOR TSC_1_2 Rictor_P Rictor Rheb_P Rheb RSK_P RSK PDK1_P PDK1 MLL_P MLL HOXA9 GBL_P GBL ERK_P ERK AKT_P AKT**
		**miRNA_1976 miRNA_196B mTOR_P mTOR TSC_1_2 Rictor_P Rictor Rheb_P Rheb RSK_P RSK PDK1_P PDK1 MLL_P MLL HOXA9 GBL_P GBL ERK_P ERK AKT_P AKT**
		**miRNA_1976 miRNA_196B mTOR_P mTOR TSC_1_2 Rictor_P Rictor Rheb_P Rheb RSK_P RSK PDK1_P PDK1 MLL_P MLL HOXA9 GBL_P GBL ERK_P ERK AKT_P AKT**
**12**	**408**	Basin Dimension Percentual: **0.41**
		**miRNA_1976 miRNA_196B mTOR_P mTOR TSC_1_2 Rictor_P Rictor Rheb_P Rheb RSK_P RSK PDK1_P PDK1 MLL_P MLL HOXA9 GBL_P GBL ERK_P ERK AKT_P AKT**

		**miRNA_1976 miRNA_196B mTOR_P mTOR TSC_1_2 Rictor_P Rictor Rheb_P Rheb RSK_P RSK PDK1_P PDK1 MLL_P MLL HOXA9 GBL_P GBL ERK_P ERK AKT_P AKT**
		**miRNA_1976 miRNA_196B mTOR_P mTOR TSC_1_2 Rictor_P Rictor Rheb_P Rheb RSK_P RSK PDK1_P PDK1 MLL_P MLL HOXA9 GBL_P GBL ERK_P ERK AKT_P AKT**
		**miRNA_1976 miRNA_196B mTOR_P mTOR TSC_1_2 Rictor_P Rictor Rheb_P Rheb RSK_P RSK PDK1_P PDK1 MLL_P MLL HOXA9 GBL_P GBL ERK_P ERK AKT_P AKT**
		**miRNA_1976 miRNA_196B mTOR_P mTOR TSC_1_2 Rictor_P Rictor Rheb_P Rheb RSK_P RSK PDK1_P PDK1 MLL_P MLL HOXA9 GBL_P GBL ERK_P ERK AKT_P AKT**
		**miRNA_1976 miRNA_196B mTOR_P mTOR TSC_1_2 Rictor_P Rictor Rheb_P Rheb RSK_P RSK PDK1_P PDK1 MLL_P MLL HOXA9 GBL_P GBL ERK_P ERK AKT_P AKT**
		**miRNA_1976 miRNA_196B mTOR_P mTOR TSC_1_2 Rictor_P Rictor Rheb_P Rheb RSK_P RSK PDK1_P PDK1 MLL_P MLL HOXA9 GBL_P GBL ERK_P ERK AKT_P AKT**
**6**	**374**	Basin Dimension Percentual: **0.38**
		**miRNA_1976 miRNA_196B mTOR_P mTOR TSC_1_2 Rictor_P Rictor Rheb_P Rheb RSK_P RSK PDK1_P PDK1 MLL_P MLL HOXA9 GBL_P GBL ERK_P ERK AKT_P AKT**
		**miRNA_1976 miRNA_196B mTOR_P mTOR TSC_1_2 Rictor_P Rictor Rheb_P Rheb RSK_P RSK PDK1_P PDK1 MLL_P MLL HOXA9 GBL_P GBL ERK_P ERK AKT_P AKT**
		**miRNA_1976 miRNA_196B mTOR_P mTOR TSC_1_2 Rictor_P Rictor Rheb_P Rheb RSK_P RSK PDK1_P PDK1 MLL_P MLL HOXA9 GBL_P GBL ERK_P ERK AKT_P AKT**
		**miRNA_1976 miRNA_196B mTOR_P mTOR TSC_1_2 Rictor_P Rictor Rheb_P Rheb RSK_P RSK PDK1_P PDK1 MLL_P MLL HOXA9 GBL_P GBL ERK_P ERK AKT_P AKT**
		**miRNA_1976 miRNA_196B mTOR_P mTOR TSC_1_2 Rictor_P Rictor Rheb_P Rheb RSK_P RSK PDK1_P PDK1 MLL_P MLL HOXA9 GBL_P GBL ERK_P ERK AKT_P AKT**
		**miRNA_1976 miRNA_196B mTOR_P mTOR TSC_1_2 Rictor_P Rictor Rheb_P Rheb RSK_P RSK PDK1_P PDK1 MLL_P MLL HOXA9 GBL_P GBL ERK_P ERK AKT_P AKT**
		**miRNA_1976 miRNA_196B mTOR_P mTOR TSC_1_2 Rictor_P Rictor Rheb_P Rheb RSK_P RSK PDK1_P PDK1 MLL_P MLL**

		**HOXA9 GBL_P GBL ERK_P ERK AKT_P AKT**
**63**	**360**	Basin Dimension Percentual: **0.36**
		**miRNA_1976 miRNA_196B mTOR_P mTOR TSC_1_2 Rictor_P Rictor Rheb_P Rheb RSK_P RSK PDK1_P PDK1 MLL_P MLL HOXA9 GBL_P GBL ERK_P ERK AKT_P AKT**
		**miRNA_1976 miRNA_196B mTOR_P mTOR TSC_1_2 Rictor_P Rictor Rheb_P Rheb RSK_P RSK PDK1_P PDK1 MLL_P MLL HOXA9 GBL_P GBL ERK_P ERK AKT_P AKT**
		**miRNA_1976 miRNA_196B mTOR_P mTOR TSC_1_2 Rictor_P Rictor Rheb_P Rheb RSK_P RSK PDK1_P PDK1 MLL_P MLL HOXA9 GBL_P GBL ERK_P ERK AKT_P AKT**
		**miRNA_1976 miRNA_196B mTOR_P mTOR TSC_1_2 Rictor_P Rictor Rheb_P Rheb RSK_P RSK PDK1_P PDK1 MLL_P MLL HOXA9 GBL_P GBL ERK_P ERK AKT_P AKT**
		**miRNA_1976 miRNA_196B mTOR_P mTOR TSC_1_2 Rictor_P Rictor Rheb_P Rheb RSK_P RSK PDK1_P PDK1 MLL_P MLL HOXA9 GBL_P GBL ERK_P ERK AKT_P AKT**
		**miRNA_1976 miRNA_196B mTOR_P mTOR TSC_1_2 Rictor_P Rictor Rheb_P Rheb RSK_P RSK PDK1_P PDK1 MLL_P MLL HOXA9 GBL_P GBL ERK_P ERK AKT_P AKT**
		**miRNA_1976 miRNA_196B mTOR_P mTOR TSC_1_2 Rictor_P Rictor Rheb_P Rheb RSK_P RSK PDK1_P PDK1 MLL_P MLL HOXA9 GBL_P GBL ERK_P ERK AKT_P AKT**
**3**	**338**	Basin Dimension Percentual: **0.34**
		**miRNA_1976 miRNA_196B mTOR_P mTOR TSC_1_2 Rictor_P Rictor Rheb_P Rheb RSK_P RSK PDK1_P PDK1 MLL_P MLL HOXA9 GBL_P GBL ERK_P ERK AKT_P AKT**
		**miRNA_1976 miRNA_196B mTOR_P mTOR TSC_1_2 Rictor_P Rictor Rheb_P Rheb RSK_P RSK PDK1_P PDK1 MLL_P MLL HOXA9 GBL_P GBL ERK_P ERK AKT_P AKT**
		**miRNA_1976 miRNA_196B mTOR_P mTOR TSC_1_2 Rictor_P Rictor Rheb_P Rheb RSK_P RSK PDK1_P PDK1 MLL_P MLL HOXA9 GBL_P GBL ERK_P ERK AKT_P AKT**
		**miRNA_1976 miRNA_196B mTOR_P mTOR TSC_1_2 Rictor_P Rictor Rheb_P Rheb RSK_P RSK PDK1_P PDK1 MLL_P MLL HOXA9 GBL_P GBL ERK_P ERK AKT_P AKT**
		**miRNA_1976 miRNA_196B mTOR_P mTOR TSC_1_2 Rictor_P Rictor Rheb_P Rheb RSK_P RSK PDK1_P PDK1 MLL_P MLL HOXA9 GBL_P GBL ERK_P ERK AKT_P AKT**

		**miRNA_1976 miRNA_196B mTOR_P mTOR TSC_1_2 Rictor_P Rictor Rheb_P Rheb RSK_P RSK PDK1_P PDK1 MLL_P MLL HOXA9 GBL_P GBL ERK_P ERK AKT_P AKT**
		**miRNA_1976 miRNA_196B mTOR_P mTOR TSC_1_2 Rictor_P Rictor Rheb_P Rheb RSK_P RSK PDK1_P PDK1 MLL_P MLL HOXA9 GBL_P GBL ERK_P ERK AKT_P AKT**
**8**	**334**	Basin Dimension Percentual: **0.34**
		**miRNA_1976 miRNA_196B mTOR_P mTOR TSC_1_2 Rictor_P Rictor Rheb_P Rheb RSK_P RSK PDK1_P PDK1 MLL_P MLL HOXA9 GBL_P GBL ERK_P ERK AKT_P AKT**
		**miRNA_1976 miRNA_196B mTOR_P mTOR TSC_1_2 Rictor_P Rictor Rheb_P Rheb RSK_P RSK PDK1_P PDK1 MLL_P MLL HOXA9 GBL_P GBL ERK_P ERK AKT_P AKT**
		**miRNA_1976 miRNA_196B mTOR_P mTOR TSC_1_2 Rictor_P Rictor Rheb_P Rheb RSK_P RSK PDK1_P PDK1 MLL_P MLL HOXA9 GBL_P GBL ERK_P ERK AKT_P AKT**
		**miRNA_1976 miRNA_196B mTOR_P mTOR TSC_1_2 Rictor_P Rictor Rheb_P Rheb RSK_P RSK PDK1_P PDK1 MLL_P MLL HOXA9 GBL_P GBL ERK_P ERK AKT_P AKT**
		**miRNA_1976 miRNA_196B mTOR_P mTOR TSC_1_2 Rictor_P Rictor Rheb_P Rheb RSK_P RSK PDK1_P PDK1 MLL_P MLL HOXA9 GBL_P GBL ERK_P ERK AKT_P AKT**
		**miRNA_1976 miRNA_196B mTOR_P mTOR TSC_1_2 Rictor_P Rictor Rheb_P Rheb RSK_P RSK PDK1_P PDK1 MLL_P MLL HOXA9 GBL_P GBL ERK_P ERK AKT_P AKT**
		**miRNA_1976 miRNA_196B mTOR_P mTOR TSC_1_2 Rictor_P Rictor Rheb_P Rheb RSK_P RSK PDK1_P PDK1 MLL_P MLL HOXA9 GBL_P GBL ERK_P ERK AKT_P AKT**
**53**	**300**	Basin Dimension Percentual: **0.30**
		**miRNA_1976 miRNA_196B mTOR_P mTOR TSC_1_2 Rictor_P Rictor Rheb_P Rheb RSK_P RSK PDK1_P PDK1 MLL_P MLL HOXA9 GBL_P GBL ERK_P ERK AKT_P AKT**
		**miRNA_1976 miRNA_196B mTOR_P mTOR TSC_1_2 Rictor_P Rictor Rheb_P Rheb RSK_P RSK PDK1_P PDK1 MLL_P MLL HOXA9 GBL_P GBL ERK_P ERK AKT_P AKT**
		**miRNA_1976 miRNA_196B mTOR_P mTOR TSC_1_2 Rictor_P Rictor Rheb_P Rheb RSK_P RSK PDK1_P PDK1 MLL_P MLL HOXA9 GBL_P GBL ERK_P ERK AKT_P AKT**

		**miRNA_1976 miRNA_196B mTOR_P mTOR TSC_1_2 Rictor_P Rictor Rheb_P Rheb RSK_P RSK PDK1_P PDK1 MLL_P MLL HOXA9 GBL_P GBL ERK_P ERK AKT_P AKT**
		**miRNA_1976 miRNA_196B mTOR_P mTOR TSC_1_2 Rictor_P Rictor Rheb_P Rheb RSK_P RSK PDK1_P PDK1 MLL_P MLL HOXA9 GBL_P GBL ERK_P ERK AKT_P AKT**
		**miRNA_1976 miRNA_196B mTOR_P mTOR TSC_1_2 Rictor_P Rictor Rheb_P Rheb RSK_P RSK PDK1_P PDK1 MLL_P MLL HOXA9 GBL_P GBL ERK_P ERK AKT_P AKT**
		**miRNA_1976 miRNA_196B mTOR_P mTOR TSC_1_2 Rictor_P Rictor Rheb_P Rheb RSK_P RSK PDK1_P PDK1 MLL_P MLL HOXA9 GBL_P GBL ERK_P ERK AKT_P AKT**
**56**	**284**	Basin Dimension Percentual: **0.28**
		**miRNA_1976 miRNA_196B mTOR_P mTOR TSC_1_2 Rictor_P Rictor Rheb_P Rheb RSK_P RSK PDK1_P PDK1 MLL_P MLL HOXA9 GBL_P GBL ERK_P ERK AKT_P AKT**
		**miRNA_1976 miRNA_196B mTOR_P mTOR TSC_1_2 Rictor_P Rictor Rheb_P Rheb RSK_P RSK PDK1_P PDK1 MLL_P MLL HOXA9 GBL_P GBL ERK_P ERK AKT_P AKT**
		**miRNA_1976 miRNA_196B mTOR_P mTOR TSC_1_2 Rictor_P Rictor Rheb_P Rheb RSK_P RSK PDK1_P PDK1 MLL_P MLL HOXA9 GBL_P GBL ERK_P ERK AKT_P AKT**
		**miRNA_1976 miRNA_196B mTOR_P mTOR TSC_1_2 Rictor_P Rictor Rheb_P Rheb RSK_P RSK PDK1_P PDK1 MLL_P MLL HOXA9 GBL_P GBL ERK_P ERK AKT_P AKT**
		**miRNA_1976 miRNA_196B mTOR_P mTOR TSC_1_2 Rictor_P Rictor Rheb_P Rheb RSK_P RSK PDK1_P PDK1 MLL_P MLL HOXA9 GBL_P GBL ERK_P ERK AKT_P AKT**
		**miRNA_1976 miRNA_196B mTOR_P mTOR TSC_1_2 Rictor_P Rictor Rheb_P Rheb RSK_P RSK PDK1_P PDK1 MLL_P MLL HOXA9 GBL_P GBL ERK_P ERK AKT_P AKT**
		**miRNA_1976 miRNA_196B mTOR_P mTOR TSC_1_2 Rictor_P Rictor Rheb_P Rheb RSK_P RSK PDK1_P PDK1 MLL_P MLL HOXA9 GBL_P GBL ERK_P ERK AKT_P AKT**
**64**	**252**	Basin Dimension Percentual: **0.25**
		**miRNA_1976 miRNA_196B mTOR_P mTOR TSC_1_2 Rictor_P Rictor Rheb_P Rheb RSK_P RSK PDK1_P PDK1 MLL_P MLL HOXA9 GBL_P GBL ERK_P ERK AKT_P AKT**

		**miRNA_1976 miRNA_196B mTOR_P mTOR TSC_1_2 Rictor_P Rictor Rheb_P Rheb RSK_P RSK PDK1_P PDK1 MLL_P MLL HOXA9 GBL_P GBL ERK_P ERK AKT_P AKT**
		**miRNA_1976 miRNA_196B mTOR_P mTOR TSC_1_2 Rictor_P Rictor Rheb_P Rheb RSK_P RSK PDK1_P PDK1 MLL_P MLL HOXA9 GBL_P GBL ERK_P ERK AKT_P AKT**
		**miRNA_1976 miRNA_196B mTOR_P mTOR TSC_1_2 Rictor_P Rictor Rheb_P Rheb RSK_P RSK PDK1_P PDK1 MLL_P MLL HOXA9 GBL_P GBL ERK_P ERK AKT_P AKT**
		**miRNA_1976 miRNA_196B mTOR_P mTOR TSC_1_2 Rictor_P Rictor Rheb_P Rheb RSK_P RSK PDK1_P PDK1 MLL_P MLL HOXA9 GBL_P GBL ERK_P ERK AKT_P AKT**
		**miRNA_1976 miRNA_196B mTOR_P mTOR TSC_1_2 Rictor_P Rictor Rheb_P Rheb RSK_P RSK PDK1_P PDK1 MLL_P MLL HOXA9 GBL_P GBL ERK_P ERK AKT_P AKT**
		**miRNA_1976 miRNA_196B mTOR_P mTOR TSC_1_2 Rictor_P Rictor Rheb_P Rheb RSK_P RSK PDK1_P PDK1 MLL_P MLL HOXA9 GBL_P GBL ERK_P ERK AKT_P AKT**
**14**	**231**	Basin Dimension Percentual: **0.23**
		**miRNA_1976 miRNA_196B mTOR_P mTOR TSC_1_2 Rictor_P Rictor Rheb_P Rheb RSK_P RSK PDK1_P PDK1 MLL_P MLL HOXA9 GBL_P GBL ERK_P ERK AKT_P AKT**
		**miRNA_1976 miRNA_196B mTOR_P mTOR TSC_1_2 Rictor_P Rictor Rheb_P Rheb RSK_P RSK PDK1_P PDK1 MLL_P MLL HOXA9 GBL_P GBL ERK_P ERK AKT_P AKT**
		**miRNA_1976 miRNA_196B mTOR_P mTOR TSC_1_2 Rictor_P Rictor Rheb_P Rheb RSK_P RSK PDK1_P PDK1 MLL_P MLL HOXA9 GBL_P GBL ERK_P ERK AKT_P AKT**
		**miRNA_1976 miRNA_196B mTOR_P mTOR TSC_1_2 Rictor_P Rictor Rheb_P Rheb RSK_P RSK PDK1_P PDK1 MLL_P MLL HOXA9 GBL_P GBL ERK_P ERK AKT_P AKT**
		**miRNA_1976 miRNA_196B mTOR_P mTOR TSC_1_2 Rictor_P Rictor Rheb_P Rheb RSK_P RSK PDK1_P PDK1 MLL_P MLL HOXA9 GBL_P GBL ERK_P ERK AKT_P AKT**
		**miRNA_1976 miRNA_196B mTOR_P mTOR TSC_1_2 Rictor_P Rictor Rheb_P Rheb RSK_P RSK PDK1_P PDK1 MLL_P MLL HOXA9 GBL_P GBL ERK_P ERK AKT_P AKT**
		**miRNA_1976 miRNA_196B mTOR_P mTOR TSC_1_2 Rictor_P Rictor Rheb_P Rheb RSK_P RSK PDK1_P PDK1 MLL_P MLL**

		**HOXA9 GBL_P GBL ERK_P ERK AKT_P AKT**
**26**	**224**	Basin Dimension Percentual: **0.22**
		**miRNA_1976 miRNA_196B mTOR_P mTOR TSC_1_2 Rictor_P Rictor Rheb_P Rheb RSK_P RSK PDK1_P PDK1 MLL_P MLL HOXA9 GBL_P GBL ERK_P ERK AKT_P AKT**
**9**	**219**	Basin Dimension Percentual: **0.22**
		**miRNA_1976 miRNA_196B mTOR_P mTOR TSC_1_2 Rictor_P Rictor Rheb_P Rheb RSK_P RSK PDK1_P PDK1 MLL_P MLL HOXA9 GBL_P GBL ERK_P ERK AKT_P AKT**
		**miRNA_1976 miRNA_196B mTOR_P mTOR TSC_1_2 Rictor_P Rictor Rheb_P Rheb RSK_P RSK PDK1_P PDK1 MLL_P MLL HOXA9 GBL_P GBL ERK_P ERK AKT_P AKT**
		**miRNA_1976 miRNA_196B mTOR_P mTOR TSC_1_2 Rictor_P Rictor Rheb_P Rheb RSK_P RSK PDK1_P PDK1 MLL_P MLL HOXA9 GBL_P GBL ERK_P ERK AKT_P AKT**
		**miRNA_1976 miRNA_196B mTOR_P mTOR TSC_1_2 Rictor_P Rictor Rheb_P Rheb RSK_P RSK PDK1_P PDK1 MLL_P MLL HOXA9 GBL_P GBL ERK_P ERK AKT_P AKT**
		**miRNA_1976 miRNA_196B mTOR_P mTOR TSC_1_2 Rictor_P Rictor Rheb_P Rheb RSK_P RSK PDK1_P PDK1 MLL_P MLL HOXA9 GBL_P GBL ERK_P ERK AKT_P AKT**
		**miRNA_1976 miRNA_196B mTOR_P mTOR TSC_1_2 Rictor_P Rictor Rheb_P Rheb RSK_P RSK PDK1_P PDK1 MLL_P MLL HOXA9 GBL_P GBL ERK_P ERK AKT_P AKT**
		**miRNA_1976 miRNA_196B mTOR_P mTOR TSC_1_2 Rictor_P Rictor Rheb_P Rheb RSK_P RSK PDK1_P PDK1 MLL_P MLL HOXA9 GBL_P GBL ERK_P ERK AKT_P AKT**
**27**	**216**	Basin Dimension Percentual: **0.22**
		**miRNA_1976 miRNA_196B mTOR_P mTOR TSC_1_2 Rictor_P Rictor Rheb_P Rheb RSK_P RSK PDK1_P PDK1 MLL_P MLL HOXA9 GBL_P GBL ERK_P ERK AKT_P AKT**
		**miRNA_1976 miRNA_196B mTOR_P mTOR TSC_1_2 Rictor_P Rictor Rheb_P Rheb RSK_P RSK PDK1_P PDK1 MLL_P MLL HOXA9 GBL_P GBL ERK_P ERK AKT_P AKT**
		**miRNA_1976 miRNA_196B mTOR_P mTOR TSC_1_2 Rictor_P Rictor Rheb_P Rheb RSK_P RSK PDK1_P PDK1 MLL_P MLL HOXA9 GBL_P GBL ERK_P ERK AKT_P AKT**

		**miRNA_1976 miRNA_196B mTOR_P mTOR TSC_1_2 Rictor_P Rictor Rheb_P Rheb RSK_P RSK PDK1_P PDK1 MLL_P MLL HOXA9 GBL_P GBL ERK_P ERK AKT_P AKT**
		**miRNA_1976 miRNA_196B mTOR_P mTOR TSC_1_2 Rictor_P Rictor Rheb_P Rheb RSK_P RSK PDK1_P PDK1 MLL_P MLL HOXA9 GBL_P GBL ERK_P ERK AKT_P AKT**
		**miRNA_1976 miRNA_196B mTOR_P mTOR TSC_1_2 Rictor_P Rictor Rheb_P Rheb RSK_P RSK PDK1_P PDK1 MLL_P MLL HOXA9 GBL_P GBL ERK_P ERK AKT_P AKT**
		**miRNA_1976 miRNA_196B mTOR_P mTOR TSC_1_2 Rictor_P Rictor Rheb_P Rheb RSK_P RSK PDK1_P PDK1 MLL_P MLL HOXA9 GBL_P GBL ERK_P ERK AKT_P AKT**
**10**	**204**	Basin Dimension Percentual: **0.20**
		**miRNA_1976 miRNA_196B mTOR_P mTOR TSC_1_2 Rictor_P Rictor Rheb_P Rheb RSK_P RSK PDK1_P PDK1 MLL_P MLL HOXA9 GBL_P GBL ERK_P ERK AKT_P AKT**
		**miRNA_1976 miRNA_196B mTOR_P mTOR TSC_1_2 Rictor_P Rictor Rheb_P Rheb RSK_P RSK PDK1_P PDK1 MLL_P MLL HOXA9 GBL_P GBL ERK_P ERK AKT_P AKT**
		**miRNA_1976 miRNA_196B mTOR_P mTOR TSC_1_2 Rictor_P Rictor Rheb_P Rheb RSK_P RSK PDK1_P PDK1 MLL_P MLL HOXA9 GBL_P GBL ERK_P ERK AKT_P AKT**
		**miRNA_1976 miRNA_196B mTOR_P mTOR TSC_1_2 Rictor_P Rictor Rheb_P Rheb RSK_P RSK PDK1_P PDK1 MLL_P MLL HOXA9 GBL_P GBL ERK_P ERK AKT_P AKT**
		**miRNA_1976 miRNA_196B mTOR_P mTOR TSC_1_2 Rictor_P Rictor Rheb_P Rheb RSK_P RSK PDK1_P PDK1 MLL_P MLL HOXA9 GBL_P GBL ERK_P ERK AKT_P AKT**
		**miRNA_1976 miRNA_196B mTOR_P mTOR TSC_1_2 Rictor_P Rictor Rheb_P Rheb RSK_P RSK PDK1_P PDK1 MLL_P MLL HOXA9 GBL_P GBL ERK_P ERK AKT_P AKT**
		**miRNA_1976 miRNA_196B mTOR_P mTOR TSC_1_2 Rictor_P Rictor Rheb_P Rheb RSK_P RSK PDK1_P PDK1 MLL_P MLL HOXA9 GBL_P GBL ERK_P ERK AKT_P AKT**
**2**	**187**	Basin Dimension Percentual: **0.19**
		**miRNA_1976 miRNA_196B mTOR_P mTOR TSC_1_2 Rictor_P Rictor Rheb_P Rheb RSK_P RSK PDK1_P PDK1 MLL_P MLL HOXA9 GBL_P GBL ERK_P ERK AKT_P AKT**
		**miRNA_1976 miRNA_196B mTOR_P mTOR TSC_1_2 Rictor_P**

		**Rictor Rheb_P Rheb RSK_P RSK PDK1_P PDK1 MLL_P MLL HOXA9 GBL_P GBL ERK_P ERK AKT_P AKT**
		**miRNA_1976 miRNA_196B mTOR_P mTOR TSC_1_2 Rictor_P Rictor Rheb_P Rheb RSK_P RSK PDK1_P PDK1 MLL_P MLL HOXA9 GBL_P GBL ERK_P ERK AKT_P AKT**
		**miRNA_1976 miRNA_196B mTOR_P mTOR TSC_1_2 Rictor_P Rictor Rheb_P Rheb RSK_P RSK PDK1_P PDK1 MLL_P MLL HOXA9 GBL_P GBL ERK_P ERK AKT_P AKT**
		**miRNA_1976 miRNA_196B mTOR_P mTOR TSC_1_2 Rictor_P Rictor Rheb_P Rheb RSK_P RSK PDK1_P PDK1 MLL_P MLL HOXA9 GBL_P GBL ERK_P ERK AKT_P AKT**
		**miRNA_1976 miRNA_196B mTOR_P mTOR TSC_1_2 Rictor_P Rictor Rheb_P Rheb RSK_P RSK PDK1_P PDK1 MLL_P MLL HOXA9 GBL_P GBL ERK_P ERK AKT_P AKT**
		**miRNA_1976 miRNA_196B mTOR_P mTOR TSC_1_2 Rictor_P Rictor Rheb_P Rheb RSK_P RSK PDK1_P PDK1 MLL_P MLL HOXA9 GBL_P GBL ERK_P ERK AKT_P AKT**
**35**	**180**	Basin Dimension Percentual: **0.18**
		**miRNA_1976 miRNA_196B mTOR_P mTOR TSC_1_2 Rictor_P Rictor Rheb_P Rheb RSK_P RSK PDK1_P PDK1 MLL_P MLL HOXA9 GBL_P GBL ERK_P ERK AKT_P AKT**
		**miRNA_1976 miRNA_196B mTOR_P mTOR TSC_1_2 Rictor_P Rictor Rheb_P Rheb RSK_P RSK PDK1_P PDK1 MLL_P MLL HOXA9 GBL_P GBL ERK_P ERK AKT_P AKT**
		**miRNA_1976 miRNA_196B mTOR_P mTOR TSC_1_2 Rictor_P Rictor Rheb_P Rheb RSK_P RSK PDK1_P PDK1 MLL_P MLL HOXA9 GBL_P GBL ERK_P ERK AKT_P AKT**
		**miRNA_1976 miRNA_196B mTOR_P mTOR TSC_1_2 Rictor_P Rictor Rheb_P Rheb RSK_P RSK PDK1_P PDK1 MLL_P MLL HOXA9 GBL_P GBL ERK_P ERK AKT_P AKT**
		**miRNA_1976 miRNA_196B mTOR_P mTOR TSC_1_2 Rictor_P Rictor Rheb_P Rheb RSK_P RSK PDK1_P PDK1 MLL_P MLL HOXA9 GBL_P GBL ERK_P ERK AKT_P AKT**
		**miRNA_1976 miRNA_196B mTOR_P mTOR TSC_1_2 Rictor_P Rictor Rheb_P Rheb RSK_P RSK PDK1_P PDK1 MLL_P MLL HOXA9 GBL_P GBL ERK_P ERK AKT_P AKT**
		**miRNA_1976 miRNA_196B mTOR_P mTOR TSC_1_2 Rictor_P Rictor Rheb_P Rheb RSK_P RSK PDK1_P PDK1 MLL_P MLL HOXA9 GBL_P GBL ERK_P ERK AKT_P AKT**

**57**	**168**	Basin Dimension Percentual: **0.17**
		**miRNA_1976 miRNA_196B mTOR_P mTOR TSC_1_2 Rictor_P Rictor Rheb_P Rheb RSK_P RSK PDK1_P PDK1 MLL_P MLL HOXA9 GBL_P GBL ERK_P ERK AKT_P AKT**
		**miRNA_1976 miRNA_196B mTOR_P mTOR TSC_1_2 Rictor_P Rictor Rheb_P Rheb RSK_P RSK PDK1_P PDK1 MLL_P MLL HOXA9 GBL_P GBL ERK_P ERK AKT_P AKT**
		**miRNA_1976 miRNA_196B mTOR_P mTOR TSC_1_2 Rictor_P Rictor Rheb_P Rheb RSK_P RSK PDK1_P PDK1 MLL_P MLL HOXA9 GBL_P GBL ERK_P ERK AKT_P AKT**
		**miRNA_1976 miRNA_196B mTOR_P mTOR TSC_1_2 Rictor_P Rictor Rheb_P Rheb RSK_P RSK PDK1_P PDK1 MLL_P MLL HOXA9 GBL_P GBL ERK_P ERK AKT_P AKT**
		**miRNA_1976 miRNA_196B mTOR_P mTOR TSC_1_2 Rictor_P Rictor Rheb_P Rheb RSK_P RSK PDK1_P PDK1 MLL_P MLL HOXA9 GBL_P GBL ERK_P ERK AKT_P AKT**
		**miRNA_1976 miRNA_196B mTOR_P mTOR TSC_1_2 Rictor_P Rictor Rheb_P Rheb RSK_P RSK PDK1_P PDK1 MLL_P MLL HOXA9 GBL_P GBL ERK_P ERK AKT_P AKT**
		**miRNA_1976 miRNA_196B mTOR_P mTOR TSC_1_2 Rictor_P Rictor Rheb_P Rheb RSK_P RSK PDK1_P PDK1 MLL_P MLL HOXA9 GBL_P GBL ERK_P ERK AKT_P AKT**
**4**	**167**	Basin Dimension Percentual: **0.17**
		**miRNA_1976 miRNA_196B mTOR_P mTOR TSC_1_2 Rictor_P Rictor Rheb_P Rheb RSK_P RSK PDK1_P PDK1 MLL_P MLL HOXA9 GBL_P GBL ERK_P ERK AKT_P AKT**
		**miRNA_1976 miRNA_196B mTOR_P mTOR TSC_1_2 Rictor_P Rictor Rheb_P Rheb RSK_P RSK PDK1_P PDK1 MLL_P MLL HOXA9 GBL_P GBL ERK_P ERK AKT_P AKT**
		**miRNA_1976 miRNA_196B mTOR_P mTOR TSC_1_2 Rictor_P Rictor Rheb_P Rheb RSK_P RSK PDK1_P PDK1 MLL_P MLL HOXA9 GBL_P GBL ERK_P ERK AKT_P AKT**
		**miRNA_1976 miRNA_196B mTOR_P mTOR TSC_1_2 Rictor_P Rictor Rheb_P Rheb RSK_P RSK PDK1_P PDK1 MLL_P MLL HOXA9 GBL_P GBL ERK_P ERK AKT_P AKT**
		**miRNA_1976 miRNA_196B mTOR_P mTOR TSC_1_2 Rictor_P Rictor Rheb_P Rheb RSK_P RSK PDK1_P PDK1 MLL_P MLL HOXA9 GBL_P GBL ERK_P ERK AKT_P AKT**
		**miRNA_1976 miRNA_196B mTOR_P mTOR TSC_1_2 Rictor_P**

		**Rictor Rheb_P Rheb RSK_P RSK PDK1_P PDK1 MLL_P MLL HOXA9 GBL_P GBL ERK_P ERK AKT_P AKT**
		**miRNA_1976 miRNA_196B mTOR_P mTOR TSC_1_2 Rictor_P Rictor Rheb_P Rheb RSK_P RSK PDK1_P PDK1 MLL_P MLL HOXA9 GBL_P GBL ERK_P ERK AKT_P AKT**
**62**	**164**	Basin Dimension Percentual: **0.16**
		**miRNA_1976 miRNA_196B mTOR_P mTOR TSC_1_2 Rictor_P Rictor Rheb_P Rheb RSK_P RSK PDK1_P PDK1 MLL_P MLL HOXA9 GBL_P GBL ERK_P ERK AKT_P AKT**
		**miRNA_1976 miRNA_196B mTOR_P mTOR TSC_1_2 Rictor_P Rictor Rheb_P Rheb RSK_P RSK PDK1_P PDK1 MLL_P MLL HOXA9 GBL_P GBL ERK_P ERK AKT_P AKT**
		**miRNA_1976 miRNA_196B mTOR_P mTOR TSC_1_2 Rictor_P Rictor Rheb_P Rheb RSK_P RSK PDK1_P PDK1 MLL_P MLL HOXA9 GBL_P GBL ERK_P ERK AKT_P AKT**
		**miRNA_1976 miRNA_196B mTOR_P mTOR TSC_1_2 Rictor_P Rictor Rheb_P Rheb RSK_P RSK PDK1_P PDK1 MLL_P MLL HOXA9 GBL_P GBL ERK_P ERK AKT_P AKT**
		**miRNA_1976 miRNA_196B mTOR_P mTOR TSC_1_2 Rictor_P Rictor Rheb_P Rheb RSK_P RSK PDK1_P PDK1 MLL_P MLL HOXA9 GBL_P GBL ERK_P ERK AKT_P AKT**
		**miRNA_1976 miRNA_196B mTOR_P mTOR TSC_1_2 Rictor_P Rictor Rheb_P Rheb RSK_P RSK PDK1_P PDK1 MLL_P MLL HOXA9 GBL_P GBL ERK_P ERK AKT_P AKT**
		**miRNA_1976 miRNA_196B mTOR_P mTOR TSC_1_2 Rictor_P Rictor Rheb_P Rheb RSK_P RSK PDK1_P PDK1 MLL_P MLL HOXA9 GBL_P GBL ERK_P ERK AKT_P AKT**
**61**	**160**	Basin Dimension Percentual: **0.16**
		**miRNA_1976 miRNA_196B mTOR_P mTOR TSC_1_2 Rictor_P Rictor Rheb_P Rheb RSK_P RSK PDK1_P PDK1 MLL_P MLL HOXA9 GBL_P GBL ERK_P ERK AKT_P AKT**
		**miRNA_1976 miRNA_196B mTOR_P mTOR TSC_1_2 Rictor_P Rictor Rheb_P Rheb RSK_P RSK PDK1_P PDK1 MLL_P MLL HOXA9 GBL_P GBL ERK_P ERK AKT_P AKT**
		**miRNA_1976 miRNA_196B mTOR_P mTOR TSC_1_2 Rictor_P Rictor Rheb_P Rheb RSK_P RSK PDK1_P PDK1 MLL_P MLL HOXA9 GBL_P GBL ERK_P ERK AKT_P AKT**
		**miRNA_1976 miRNA_196B mTOR_P mTOR TSC_1_2 Rictor_P Rictor Rheb_P Rheb RSK_P RSK PDK1_P PDK1 MLL_P MLL**

		**HOXA9 GBL_P GBL ERK_P ERK AKT_P AKT**
		**miRNA_1976 miRNA_196B mTOR_P mTOR TSC_1_2 Rictor_P Rictor Rheb_P Rheb RSK_P RSK PDK1_P PDK1 MLL_P MLL HOXA9 GBL_P GBL ERK_P ERK AKT_P AKT**
		**miRNA_1976 miRNA_196B mTOR_P mTOR TSC_1_2 Rictor_P Rictor Rheb_P Rheb RSK_P RSK PDK1_P PDK1 MLL_P MLL HOXA9 GBL_P GBL ERK_P ERK AKT_P AKT**
		**miRNA_1976 miRNA_196B mTOR_P mTOR TSC_1_2 Rictor_P Rictor Rheb_P Rheb RSK_P RSK PDK1_P PDK1 MLL_P MLL HOXA9 GBL_P GBL ERK_P ERK AKT_P AKT**
**16**	**150**	Basin Dimension Percentual: **0.15**
		**miRNA_1976 miRNA_196B mTOR_P mTOR TSC_1_2 Rictor_P Rictor Rheb_P Rheb RSK_P RSK PDK1_P PDK1 MLL_P MLL HOXA9 GBL_P GBL ERK_P ERK AKT_P AKT**
		**miRNA_1976 miRNA_196B mTOR_P mTOR TSC_1_2 Rictor_P Rictor Rheb_P Rheb RSK_P RSK PDK1_P PDK1 MLL_P MLL HOXA9 GBL_P GBL ERK_P ERK AKT_P AKT**
		**miRNA_1976 miRNA_196B mTOR_P mTOR TSC_1_2 Rictor_P Rictor Rheb_P Rheb RSK_P RSK PDK1_P PDK1 MLL_P MLL HOXA9 GBL_P GBL ERK_P ERK AKT_P AKT**
		**miRNA_1976 miRNA_196B mTOR_P mTOR TSC_1_2 Rictor_P Rictor Rheb_P Rheb RSK_P RSK PDK1_P PDK1 MLL_P MLL HOXA9 GBL_P GBL ERK_P ERK AKT_P AKT**
		**miRNA_1976 miRNA_196B mTOR_P mTOR TSC_1_2 Rictor_P Rictor Rheb_P Rheb RSK_P RSK PDK1_P PDK1 MLL_P MLL HOXA9 GBL_P GBL ERK_P ERK AKT_P AKT**
		**miRNA_1976 miRNA_196B mTOR_P mTOR TSC_1_2 Rictor_P Rictor Rheb_P Rheb RSK_P RSK PDK1_P PDK1 MLL_P MLL HOXA9 GBL_P GBL ERK_P ERK AKT_P AKT**
		**miRNA_1976 miRNA_196B mTOR_P mTOR TSC_1_2 Rictor_P Rictor Rheb_P Rheb RSK_P RSK PDK1_P PDK1 MLL_P MLL HOXA9 GBL_P GBL ERK_P ERK AKT_P AKT**
**21**	**142**	Basin Dimension Percentual: **0.14**
		**miRNA_1976 miRNA_196B mTOR_P mTOR TSC_1_2 Rictor_P Rictor Rheb_P Rheb RSK_P RSK PDK1_P PDK1 MLL_P MLL HOXA9 GBL_P GBL ERK_P ERK AKT_P AKT**
		**miRNA_1976 miRNA_196B mTOR_P mTOR TSC_1_2 Rictor_P Rictor Rheb_P Rheb RSK_P RSK PDK1_P PDK1 MLL_P MLL HOXA9 GBL_P GBL ERK_P ERK AKT_P AKT**

		**miRNA_1976 miRNA_196B mTOR_P mTOR TSC_1_2 Rictor_P Rictor Rheb_P Rheb RSK_P RSK PDK1_P PDK1 MLL_P MLL HOXA9 GBL_P GBL ERK_P ERK AKT_P AKT**
		**miRNA_1976 miRNA_196B mTOR_P mTOR TSC_1_2 Rictor_P Rictor Rheb_P Rheb RSK_P RSK PDK1_P PDK1 MLL_P MLL HOXA9 GBL_P GBL ERK_P ERK AKT_P AKT**
		**miRNA_1976 miRNA_196B mTOR_P mTOR TSC_1_2 Rictor_P Rictor Rheb_P Rheb RSK_P RSK PDK1_P PDK1 MLL_P MLL HOXA9 GBL_P GBL ERK_P ERK AKT_P AKT**
		**miRNA_1976 miRNA_196B mTOR_P mTOR TSC_1_2 Rictor_P Rictor Rheb_P Rheb RSK_P RSK PDK1_P PDK1 MLL_P MLL HOXA9 GBL_P GBL ERK_P ERK AKT_P AKT**
		**miRNA_1976 miRNA_196B mTOR_P mTOR TSC_1_2 Rictor_P Rictor Rheb_P Rheb RSK_P RSK PDK1_P PDK1 MLL_P MLL HOXA9 GBL_P GBL ERK_P ERK AKT_P AKT**
**36**	**126**	Basin Dimension Percentual: **0.13**
		**miRNA_1976 miRNA_196B mTOR_P mTOR TSC_1_2 Rictor_P Rictor Rheb_P Rheb RSK_P RSK PDK1_P PDK1 MLL_P MLL HOXA9 GBL_P GBL ERK_P ERK AKT_P AKT**
		**miRNA_1976 miRNA_196B mTOR_P mTOR TSC_1_2 Rictor_P Rictor Rheb_P Rheb RSK_P RSK PDK1_P PDK1 MLL_P MLL HOXA9 GBL_P GBL ERK_P ERK AKT_P AKT**
		**miRNA_1976 miRNA_196B mTOR_P mTOR TSC_1_2 Rictor_P Rictor Rheb_P Rheb RSK_P RSK PDK1_P PDK1 MLL_P MLL HOXA9 GBL_P GBL ERK_P ERK AKT_P AKT**
		**miRNA_1976 miRNA_196B mTOR_P mTOR TSC_1_2 Rictor_P Rictor Rheb_P Rheb RSK_P RSK PDK1_P PDK1 MLL_P MLL HOXA9 GBL_P GBL ERK_P ERK AKT_P AKT**
		**miRNA_1976 miRNA_196B mTOR_P mTOR TSC_1_2 Rictor_P Rictor Rheb_P Rheb RSK_P RSK PDK1_P PDK1 MLL_P MLL HOXA9 GBL_P GBL ERK_P ERK AKT_P AKT**
		**miRNA_1976 miRNA_196B mTOR_P mTOR TSC_1_2 Rictor_P Rictor Rheb_P Rheb RSK_P RSK PDK1_P PDK1 MLL_P MLL HOXA9 GBL_P GBL ERK_P ERK AKT_P AKT**
		**miRNA_1976 miRNA_196B mTOR_P mTOR TSC_1_2 Rictor_P Rictor Rheb_P Rheb RSK_P RSK PDK1_P PDK1 MLL_P MLL HOXA9 GBL_P GBL ERK_P ERK AKT_P AKT**
**25**	**112**	Basin Dimension Percentual: **0.11**

		**miRNA_1976 miRNA_196B mTOR_P mTOR TSC_1_2 Rictor_P Rictor Rheb_P Rheb RSK_P RSK PDK1_P PDK1 MLL_P MLL HOXA9 GBL_P GBL ERK_P ERK AKT_P AKT**
**33**	**90**	Basin Dimension Percentual: **0.09**
		**miRNA_1976 miRNA_196B mTOR_P mTOR TSC_1_2 Rictor_P Rictor Rheb_P Rheb RSK_P RSK PDK1_P PDK1 MLL_P MLL HOXA9 GBL_P GBL ERK_P ERK AKT_P AKT**
		**miRNA_1976 miRNA_196B mTOR_P mTOR TSC_1_2 Rictor_P Rictor Rheb_P Rheb RSK_P RSK PDK1_P PDK1 MLL_P MLL HOXA9 GBL_P GBL ERK_P ERK AKT_P AKT**
		**miRNA_1976 miRNA_196B mTOR_P mTOR TSC_1_2 Rictor_P Rictor Rheb_P Rheb RSK_P RSK PDK1_P PDK1 MLL_P MLL HOXA9 GBL_P GBL ERK_P ERK AKT_P AKT**
		**miRNA_1976 miRNA_196B mTOR_P mTOR TSC_1_2 Rictor_P Rictor Rheb_P Rheb RSK_P RSK PDK1_P PDK1 MLL_P MLL HOXA9 GBL_P GBL ERK_P ERK AKT_P AKT**
		**miRNA_1976 miRNA_196B mTOR_P mTOR TSC_1_2 Rictor_P Rictor Rheb_P Rheb RSK_P RSK PDK1_P PDK1 MLL_P MLL HOXA9 GBL_P GBL ERK_P ERK AKT_P AKT**
		**miRNA_1976 miRNA_196B mTOR_P mTOR TSC_1_2 Rictor_P Rictor Rheb_P Rheb RSK_P RSK PDK1_P PDK1 MLL_P MLL HOXA9 GBL_P GBL ERK_P ERK AKT_P AKT**
		**miRNA_1976 miRNA_196B mTOR_P mTOR TSC_1_2 Rictor_P Rictor Rheb_P Rheb RSK_P RSK PDK1_P PDK1 MLL_P MLL HOXA9 GBL_P GBL ERK_P ERK AKT_P AKT**
**22**	**84**	Basin Dimension Percentual: **0.08**
		**miRNA_1976 miRNA_196B mTOR_P mTOR TSC_1_2 Rictor_P Rictor Rheb_P Rheb RSK_P RSK PDK1_P PDK1 MLL_P MLL HOXA9 GBL_P GBL ERK_P ERK AKT_P AKT**
		**miRNA_1976 miRNA_196B mTOR_P mTOR TSC_1_2 Rictor_P Rictor Rheb_P Rheb RSK_P RSK PDK1_P PDK1 MLL_P MLL HOXA9 GBL_P GBL ERK_P ERK AKT_P AKT**
		**miRNA_1976 miRNA_196B mTOR_P mTOR TSC_1_2 Rictor_P Rictor Rheb_P Rheb RSK_P RSK PDK1_P PDK1 MLL_P MLL HOXA9 GBL_P GBL ERK_P ERK AKT_P AKT**
		**miRNA_1976 miRNA_196B mTOR_P mTOR TSC_1_2 Rictor_P Rictor Rheb_P Rheb RSK_P RSK PDK1_P PDK1 MLL_P MLL HOXA9 GBL_P GBL ERK_P ERK AKT_P AKT**

		**miRNA_1976 miRNA_196B mTOR_P mTOR TSC_1_2 Rictor_P Rictor Rheb_P Rheb RSK_P RSK PDK1_P PDK1 MLL_P MLL HOXA9 GBL_P GBL ERK_P ERK AKT_P AKT**
		**miRNA_1976 miRNA_196B mTOR_P mTOR TSC_1_2 Rictor_P Rictor Rheb_P Rheb RSK_P RSK PDK1_P PDK1 MLL_P MLL HOXA9 GBL_P GBL ERK_P ERK AKT_P AKT**
		**miRNA_1976 miRNA_196B mTOR_P mTOR TSC_1_2 Rictor_P Rictor Rheb_P Rheb RSK_P RSK PDK1_P PDK1 MLL_P MLL HOXA9 GBL_P GBL ERK_P ERK AKT_P AKT**
**32**	**82**	Basin Dimension Percentual: **0.08**
		**miRNA_1976 miRNA_196B mTOR_P mTOR TSC_1_2 Rictor_P Rictor Rheb_P Rheb RSK_P RSK PDK1_P PDK1 MLL_P MLL HOXA9 GBL_P GBL ERK_P ERK AKT_P AKT**
		**miRNA_1976 miRNA_196B mTOR_P mTOR TSC_1_2 Rictor_P Rictor Rheb_P Rheb RSK_P RSK PDK1_P PDK1 MLL_P MLL HOXA9 GBL_P GBL ERK_P ERK AKT_P AKT**
		**miRNA_1976 miRNA_196B mTOR_P mTOR TSC_1_2 Rictor_P Rictor Rheb_P Rheb RSK_P RSK PDK1_P PDK1 MLL_P MLL HOXA9 GBL_P GBL ERK_P ERK AKT_P AKT**
		**miRNA_1976 miRNA_196B mTOR_P mTOR TSC_1_2 Rictor_P Rictor Rheb_P Rheb RSK_P RSK PDK1_P PDK1 MLL_P MLL HOXA9 GBL_P GBL ERK_P ERK AKT_P AKT**
		**miRNA_1976 miRNA_196B mTOR_P mTOR TSC_1_2 Rictor_P Rictor Rheb_P Rheb RSK_P RSK PDK1_P PDK1 MLL_P MLL HOXA9 GBL_P GBL ERK_P ERK AKT_P AKT**
		**miRNA_1976 miRNA_196B mTOR_P mTOR TSC_1_2 Rictor_P Rictor Rheb_P Rheb RSK_P RSK PDK1_P PDK1 MLL_P MLL HOXA9 GBL_P GBL ERK_P ERK AKT_P AKT**
		**miRNA_1976 miRNA_196B mTOR_P mTOR TSC_1_2 Rictor_P Rictor Rheb_P Rheb RSK_P RSK PDK1_P PDK1 MLL_P MLL HOXA9 GBL_P GBL ERK_P ERK AKT_P AKT**
**30**	**80**	Basin Dimension Percentual: **0.08**
		**miRNA_1976 miRNA_196B mTOR_P mTOR TSC_1_2 Rictor_P Rictor Rheb_P Rheb RSK_P RSK PDK1_P PDK1 MLL_P MLL HOXA9 GBL_P GBL ERK_P ERK AKT_P AKT**
		**miRNA_1976 miRNA_196B mTOR_P mTOR TSC_1_2 Rictor_P Rictor Rheb_P Rheb RSK_P RSK PDK1_P PDK1 MLL_P MLL HOXA9 GBL_P GBL ERK_P ERK AKT_P AKT**

		**miRNA_1976 miRNA_196B mTOR_P mTOR TSC_1_2 Rictor_P Rictor Rheb_P Rheb RSK_P RSK PDK1_P PDK1 MLL_P MLL HOXA9 GBL_P GBL ERK_P ERK AKT_P AKT**
		**miRNA_1976 miRNA_196B mTOR_P mTOR TSC_1_2 Rictor_P Rictor Rheb_P Rheb RSK_P RSK PDK1_P PDK1 MLL_P MLL HOXA9 GBL_P GBL ERK_P ERK AKT_P AKT**
		**miRNA_1976 miRNA_196B mTOR_P mTOR TSC_1_2 Rictor_P Rictor Rheb_P Rheb RSK_P RSK PDK1_P PDK1 MLL_P MLL HOXA9 GBL_P GBL ERK_P ERK AKT_P AKT**
		**miRNA_1976 miRNA_196B mTOR_P mTOR TSC_1_2 Rictor_P Rictor Rheb_P Rheb RSK_P RSK PDK1_P PDK1 MLL_P MLL HOXA9 GBL_P GBL ERK_P ERK AKT_P AKT**
		**miRNA_1976 miRNA_196B mTOR_P mTOR TSC_1_2 Rictor_P Rictor Rheb_P Rheb RSK_P RSK PDK1_P PDK1 MLL_P MLL HOXA9 GBL_P GBL ERK_P ERK AKT_P AKT**
**66**	**80**	Basin Dimension Percentual: **0.08**
		**miRNA_1976 miRNA_196B mTOR_P mTOR TSC_1_2 Rictor_P Rictor Rheb_P Rheb RSK_P RSK PDK1_P PDK1 MLL_P MLL HOXA9 GBL_P GBL ERK_P ERK AKT_P AKT**
		**miRNA_1976 miRNA_196B mTOR_P mTOR TSC_1_2 Rictor_P Rictor Rheb_P Rheb RSK_P RSK PDK1_P PDK1 MLL_P MLL HOXA9 GBL_P GBL ERK_P ERK AKT_P AKT**
		**miRNA_1976 miRNA_196B mTOR_P mTOR TSC_1_2 Rictor_P Rictor Rheb_P Rheb RSK_P RSK PDK1_P PDK1 MLL_P MLL HOXA9 GBL_P GBL ERK_P ERK AKT_P AKT**
		**miRNA_1976 miRNA_196B mTOR_P mTOR TSC_1_2 Rictor_P Rictor Rheb_P Rheb RSK_P RSK PDK1_P PDK1 MLL_P MLL HOXA9 GBL_P GBL ERK_P ERK AKT_P AKT**
		**miRNA_1976 miRNA_196B mTOR_P mTOR TSC_1_2 Rictor_P Rictor Rheb_P Rheb RSK_P RSK PDK1_P PDK1 MLL_P MLL HOXA9 GBL_P GBL ERK_P ERK AKT_P AKT**
		**miRNA_1976 miRNA_196B mTOR_P mTOR TSC_1_2 Rictor_P Rictor Rheb_P Rheb RSK_P RSK PDK1_P PDK1 MLL_P MLL HOXA9 GBL_P GBL ERK_P ERK AKT_P AKT**
		**miRNA_1976 miRNA_196B mTOR_P mTOR TSC_1_2 Rictor_P Rictor Rheb_P Rheb RSK_P RSK PDK1_P PDK1 MLL_P MLL HOXA9 GBL_P GBL ERK_P ERK AKT_P AKT**
**13**	**75**	Basin Dimension Percentual: **0.08**

		**miRNA_1976 miRNA_196B mTOR_P mTOR TSC_1_2 Rictor_P Rictor Rheb_P Rheb RSK_P RSK PDK1_P PDK1 MLL_P MLL HOXA9 GBL_P GBL ERK_P ERK AKT_P AKT**
		**miRNA_1976 miRNA_196B mTOR_P mTOR TSC_1_2 Rictor_P Rictor Rheb_P Rheb RSK_P RSK PDK1_P PDK1 MLL_P MLL HOXA9 GBL_P GBL ERK_P ERK AKT_P AKT**
		**miRNA_1976 miRNA_196B mTOR_P mTOR TSC_1_2 Rictor_P Rictor Rheb_P Rheb RSK_P RSK PDK1_P PDK1 MLL_P MLL HOXA9 GBL_P GBL ERK_P ERK AKT_P AKT**
		**miRNA_1976 miRNA_196B mTOR_P mTOR TSC_1_2 Rictor_P Rictor Rheb_P Rheb RSK_P RSK PDK1_P PDK1 MLL_P MLL HOXA9 GBL_P GBL ERK_P ERK AKT_P AKT**
		**miRNA_1976 miRNA_196B mTOR_P mTOR TSC_1_2 Rictor_P Rictor Rheb_P Rheb RSK_P RSK PDK1_P PDK1 MLL_P MLL HOXA9 GBL_P GBL ERK_P ERK AKT_P AKT**
		**miRNA_1976 miRNA_196B mTOR_P mTOR TSC_1_2 Rictor_P Rictor Rheb_P Rheb RSK_P RSK PDK1_P PDK1 MLL_P MLL HOXA9 GBL_P GBL ERK_P ERK AKT_P AKT**
		**miRNA_1976 miRNA_196B mTOR_P mTOR TSC_1_2 Rictor_P Rictor Rheb_P Rheb RSK_P RSK PDK1_P PDK1 MLL_P MLL HOXA9 GBL_P GBL ERK_P ERK AKT_P AKT**
**65**	**72**	Basin Dimension Percentual: **0.07**
		**miRNA_1976 miRNA_196B mTOR_P mTOR TSC_1_2 Rictor_P Rictor Rheb_P Rheb RSK_P RSK PDK1_P PDK1 MLL_P MLL HOXA9 GBL_P GBL ERK_P ERK AKT_P AKT**
		**miRNA_1976 miRNA_196B mTOR_P mTOR TSC_1_2 Rictor_P Rictor Rheb_P Rheb RSK_P RSK PDK1_P PDK1 MLL_P MLL HOXA9 GBL_P GBL ERK_P ERK AKT_P AKT**
		**miRNA_1976 miRNA_196B mTOR_P mTOR TSC_1_2 Rictor_P Rictor Rheb_P Rheb RSK_P RSK PDK1_P PDK1 MLL_P MLL HOXA9 GBL_P GBL ERK_P ERK AKT_P AKT**
		**miRNA_1976 miRNA_196B mTOR_P mTOR TSC_1_2 Rictor_P Rictor Rheb_P Rheb RSK_P RSK PDK1_P PDK1 MLL_P MLL HOXA9 GBL_P GBL ERK_P ERK AKT_P AKT**
		**miRNA_1976 miRNA_196B mTOR_P mTOR TSC_1_2 Rictor_P Rictor Rheb_P Rheb RSK_P RSK PDK1_P PDK1 MLL_P MLL HOXA9 GBL_P GBL ERK_P ERK AKT_P AKT**
		**miRNA_1976 miRNA_196B mTOR_P mTOR TSC_1_2 Rictor_P Rictor Rheb_P Rheb RSK_P RSK PDK1_P PDK1 MLL_P MLL**

		**HOXA9 GBL_P GBL ERK_P ERK AKT_P AKT**
		**miRNA_1976 miRNA_196B mTOR_P mTOR TSC_1_2 Rictor_P Rictor Rheb_P Rheb RSK_P RSK PDK1_P PDK1 MLL_P MLL HOXA9 GBL_P GBL ERK_P ERK AKT_P AKT**
**19**	**71**	Basin Dimension Percentual: **0.07**
		**miRNA_1976 miRNA_196B mTOR_P mTOR TSC_1_2 Rictor_P Rictor Rheb_P Rheb RSK_P RSK PDK1_P PDK1 MLL_P MLL HOXA9 GBL_P GBL ERK_P ERK AKT_P AKT**
		**miRNA_1976 miRNA_196B mTOR_P mTOR TSC_1_2 Rictor_P Rictor Rheb_P Rheb RSK_P RSK PDK1_P PDK1 MLL_P MLL HOXA9 GBL_P GBL ERK_P ERK AKT_P AKT**
		**miRNA_1976 miRNA_196B mTOR_P mTOR TSC_1_2 Rictor_P Rictor Rheb_P Rheb RSK_P RSK PDK1_P PDK1 MLL_P MLL HOXA9 GBL_P GBL ERK_P ERK AKT_P AKT**
		**miRNA_1976 miRNA_196B mTOR_P mTOR TSC_1_2 Rictor_P Rictor Rheb_P Rheb RSK_P RSK PDK1_P PDK1 MLL_P MLL HOXA9 GBL_P GBL ERK_P ERK AKT_P AKT**
		**miRNA_1976 miRNA_196B mTOR_P mTOR TSC_1_2 Rictor_P Rictor Rheb_P Rheb RSK_P RSK PDK1_P PDK1 MLL_P MLL HOXA9 GBL_P GBL ERK_P ERK AKT_P AKT**
		**miRNA_1976 miRNA_196B mTOR_P mTOR TSC_1_2 Rictor_P Rictor Rheb_P Rheb RSK_P RSK PDK1_P PDK1 MLL_P MLL HOXA9 GBL_P GBL ERK_P ERK AKT_P AKT**
		**miRNA_1976 miRNA_196B mTOR_P mTOR TSC_1_2 Rictor_P Rictor Rheb_P Rheb RSK_P RSK PDK1_P PDK1 MLL_P MLL HOXA9 GBL_P GBL ERK_P ERK AKT_P AKT**
**55**	**68**	Basin Dimension Percentual: **0.07**
		**miRNA_1976 miRNA_196B mTOR_P mTOR TSC_1_2 Rictor_P Rictor Rheb_P Rheb RSK_P RSK PDK1_P PDK1 MLL_P MLL HOXA9 GBL_P GBL ERK_P ERK AKT_P AKT**
		**miRNA_1976 miRNA_196B mTOR_P mTOR TSC_1_2 Rictor_P Rictor Rheb_P Rheb RSK_P RSK PDK1_P PDK1 MLL_P MLL HOXA9 GBL_P GBL ERK_P ERK AKT_P AKT**
		**miRNA_1976 miRNA_196B mTOR_P mTOR TSC_1_2 Rictor_P Rictor Rheb_P Rheb RSK_P RSK PDK1_P PDK1 MLL_P MLL HOXA9 GBL_P GBL ERK_P ERK AKT_P AKT**
		**miRNA_1976 miRNA_196B mTOR_P mTOR TSC_1_2 Rictor_P Rictor Rheb_P Rheb RSK_P RSK PDK1_P PDK1 MLL_P MLL HOXA9 GBL_P GBL ERK_P ERK AKT_P AKT**

		**miRNA_1976 miRNA_196B mTOR_P mTOR TSC_1_2 Rictor_P Rictor Rheb_P Rheb RSK_P RSK PDK1_P PDK1 MLL_P MLL HOXA9 GBL_P GBL ERK_P ERK AKT_P AKT**
		**miRNA_1976 miRNA_196B mTOR_P mTOR TSC_1_2 Rictor_P Rictor Rheb_P Rheb RSK_P RSK PDK1_P PDK1 MLL_P MLL HOXA9 GBL_P GBL ERK_P ERK AKT_P AKT**
		**miRNA_1976 miRNA_196B mTOR_P mTOR TSC_1_2 Rictor_P Rictor Rheb_P Rheb RSK_P RSK PDK1_P PDK1 MLL_P MLL HOXA9 GBL_P GBL ERK_P ERK AKT_P AKT**
**34**	**63**	Basin Dimension Percentual: **0.06**
		**miRNA_1976 miRNA_196B mTOR_P mTOR TSC_1_2 Rictor_P Rictor Rheb_P Rheb RSK_P RSK PDK1_P PDK1 MLL_P MLL HOXA9 GBL_P GBL ERK_P ERK AKT_P AKT**
		**miRNA_1976 miRNA_196B mTOR_P mTOR TSC_1_2 Rictor_P Rictor Rheb_P Rheb RSK_P RSK PDK1_P PDK1 MLL_P MLL HOXA9 GBL_P GBL ERK_P ERK AKT_P AKT**
		**miRNA_1976 miRNA_196B mTOR_P mTOR TSC_1_2 Rictor_P Rictor Rheb_P Rheb RSK_P RSK PDK1_P PDK1 MLL_P MLL HOXA9 GBL_P GBL ERK_P ERK AKT_P AKT**
		**miRNA_1976 miRNA_196B mTOR_P mTOR TSC_1_2 Rictor_P Rictor Rheb_P Rheb RSK_P RSK PDK1_P PDK1 MLL_P MLL HOXA9 GBL_P GBL ERK_P ERK AKT_P AKT**
		**miRNA_1976 miRNA_196B mTOR_P mTOR TSC_1_2 Rictor_P Rictor Rheb_P Rheb RSK_P RSK PDK1_P PDK1 MLL_P MLL HOXA9 GBL_P GBL ERK_P ERK AKT_P AKT**
		**miRNA_1976 miRNA_196B mTOR_P mTOR TSC_1_2 Rictor_P Rictor Rheb_P Rheb RSK_P RSK PDK1_P PDK1 MLL_P MLL HOXA9 GBL_P GBL ERK_P ERK AKT_P AKT**
		**miRNA_1976 miRNA_196B mTOR_P mTOR TSC_1_2 Rictor_P Rictor Rheb_P Rheb RSK_P RSK PDK1_P PDK1 MLL_P MLL HOXA9 GBL_P GBL ERK_P ERK AKT_P AKT**
**20**	**42**	Basin Dimension Percentual: **0.04**
		**miRNA_1976 miRNA_196B mTOR_P mTOR TSC_1_2 Rictor_P Rictor Rheb_P Rheb RSK_P RSK PDK1_P PDK1 MLL_P MLL HOXA9 GBL_P GBL ERK_P ERK AKT_P AKT**
		**miRNA_1976 miRNA_196B mTOR_P mTOR TSC_1_2 Rictor_P Rictor Rheb_P Rheb RSK_P RSK PDK1_P PDK1 MLL_P MLL HOXA9 GBL_P GBL ERK_P ERK AKT_P AKT**

		**miRNA_1976 miRNA_196B mTOR_P mTOR TSC_1_2 Rictor_P Rictor Rheb_P Rheb RSK_P RSK PDK1_P PDK1 MLL_P MLL HOXA9 GBL_P GBL ERK_P ERK AKT_P AKT**
		**miRNA_1976 miRNA_196B mTOR_P mTOR TSC_1_2 Rictor_P Rictor Rheb_P Rheb RSK_P RSK PDK1_P PDK1 MLL_P MLL HOXA9 GBL_P GBL ERK_P ERK AKT_P AKT**
		**miRNA_1976 miRNA_196B mTOR_P mTOR TSC_1_2 Rictor_P Rictor Rheb_P Rheb RSK_P RSK PDK1_P PDK1 MLL_P MLL HOXA9 GBL_P GBL ERK_P ERK AKT_P AKT**
		**miRNA_1976 miRNA_196B mTOR_P mTOR TSC_1_2 Rictor_P Rictor Rheb_P Rheb RSK_P RSK PDK1_P PDK1 MLL_P MLL HOXA9 GBL_P GBL ERK_P ERK AKT_P AKT**
		**miRNA_1976 miRNA_196B mTOR_P mTOR TSC_1_2 Rictor_P Rictor Rheb_P Rheb RSK_P RSK PDK1_P PDK1 MLL_P MLL HOXA9 GBL_P GBL ERK_P ERK AKT_P AKT**
**31**	**41**	Basin Dimension Percentual: **0.04**
		**miRNA_1976 miRNA_196B mTOR_P mTOR TSC_1_2 Rictor_P Rictor Rheb_P Rheb RSK_P RSK PDK1_P PDK1 MLL_P MLL HOXA9 GBL_P GBL ERK_P ERK AKT_P AKT**
		**miRNA_1976 miRNA_196B mTOR_P mTOR TSC_1_2 Rictor_P Rictor Rheb_P Rheb RSK_P RSK PDK1_P PDK1 MLL_P MLL HOXA9 GBL_P GBL ERK_P ERK AKT_P AKT**
		**miRNA_1976 miRNA_196B mTOR_P mTOR TSC_1_2 Rictor_P Rictor Rheb_P Rheb RSK_P RSK PDK1_P PDK1 MLL_P MLL HOXA9 GBL_P GBL ERK_P ERK AKT_P AKT**
		**miRNA_1976 miRNA_196B mTOR_P mTOR TSC_1_2 Rictor_P Rictor Rheb_P Rheb RSK_P RSK PDK1_P PDK1 MLL_P MLL HOXA9 GBL_P GBL ERK_P ERK AKT_P AKT**
		**miRNA_1976 miRNA_196B mTOR_P mTOR TSC_1_2 Rictor_P Rictor Rheb_P Rheb RSK_P RSK PDK1_P PDK1 MLL_P MLL HOXA9 GBL_P GBL ERK_P ERK AKT_P AKT**
		**miRNA_1976 miRNA_196B mTOR_P mTOR TSC_1_2 Rictor_P Rictor Rheb_P Rheb RSK_P RSK PDK1_P PDK1 MLL_P MLL HOXA9 GBL_P GBL ERK_P ERK AKT_P AKT**
		**miRNA_1976 miRNA_196B mTOR_P mTOR TSC_1_2 Rictor_P Rictor Rheb_P Rheb RSK_P RSK PDK1_P PDK1 MLL_P MLL HOXA9 GBL_P GBL ERK_P ERK AKT_P AKT**
**29**	**40**	Basin Dimension Percentual: **0.04**

		**miRNA_1976 miRNA_196B mTOR_P mTOR TSC_1_2 Rictor_P Rictor Rheb_P Rheb RSK_P RSK PDK1_P PDK1 MLL_P MLL HOXA9 GBL_P GBL ERK_P ERK AKT_P AKT**
		**miRNA_1976 miRNA_196B mTOR_P mTOR TSC_1_2 Rictor_P Rictor Rheb_P Rheb RSK_P RSK PDK1_P PDK1 MLL_P MLL HOXA9 GBL_P GBL ERK_P ERK AKT_P AKT**
		**miRNA_1976 miRNA_196B mTOR_P mTOR TSC_1_2 Rictor_P Rictor Rheb_P Rheb RSK_P RSK PDK1_P PDK1 MLL_P MLL HOXA9 GBL_P GBL ERK_P ERK AKT_P AKT**
		**miRNA_1976 miRNA_196B mTOR_P mTOR TSC_1_2 Rictor_P Rictor Rheb_P Rheb RSK_P RSK PDK1_P PDK1 MLL_P MLL HOXA9 GBL_P GBL ERK_P ERK AKT_P AKT**
		**miRNA_1976 miRNA_196B mTOR_P mTOR TSC_1_2 Rictor_P Rictor Rheb_P Rheb RSK_P RSK PDK1_P PDK1 MLL_P MLL HOXA9 GBL_P GBL ERK_P ERK AKT_P AKT**
		**miRNA_1976 miRNA_196B mTOR_P mTOR TSC_1_2 Rictor_P Rictor Rheb_P Rheb RSK_P RSK PDK1_P PDK1 MLL_P MLL HOXA9 GBL_P GBL ERK_P ERK AKT_P AKT**
		**miRNA_1976 miRNA_196B mTOR_P mTOR TSC_1_2 Rictor_P Rictor Rheb_P Rheb RSK_P RSK PDK1_P PDK1 MLL_P MLL HOXA9 GBL_P GBL ERK_P ERK AKT_P AKT**
**40**	**40**	Basin Dimension Percentual: **0.04**
		**miRNA_1976 miRNA_196B mTOR_P mTOR TSC_1_2 Rictor_P Rictor Rheb_P Rheb RSK_P RSK PDK1_P PDK1 MLL_P MLL HOXA9 GBL_P GBL ERK_P ERK AKT_P AKT**
		**miRNA_1976 miRNA_196B mTOR_P mTOR TSC_1_2 Rictor_P Rictor Rheb_P Rheb RSK_P RSK PDK1_P PDK1 MLL_P MLL HOXA9 GBL_P GBL ERK_P ERK AKT_P AKT**
		**miRNA_1976 miRNA_196B mTOR_P mTOR TSC_1_2 Rictor_P Rictor Rheb_P Rheb RSK_P RSK PDK1_P PDK1 MLL_P MLL HOXA9 GBL_P GBL ERK_P ERK AKT_P AKT**
		**miRNA_1976 miRNA_196B mTOR_P mTOR TSC_1_2 Rictor_P Rictor Rheb_P Rheb RSK_P RSK PDK1_P PDK1 MLL_P MLL HOXA9 GBL_P GBL ERK_P ERK AKT_P AKT**
		**miRNA_1976 miRNA_196B mTOR_P mTOR TSC_1_2 Rictor_P Rictor Rheb_P Rheb RSK_P RSK PDK1_P PDK1 MLL_P MLL HOXA9 GBL_P GBL ERK_P ERK AKT_P AKT**
		**miRNA_1976 miRNA_196B mTOR_P mTOR TSC_1_2 Rictor_P Rictor Rheb_P Rheb RSK_P RSK PDK1_P PDK1 MLL_P MLL**

		**HOXA9 GBL_P GBL ERK_P ERK AKT_P AKT**
		**miRNA_1976 miRNA_196B mTOR_P mTOR TSC_1_2 Rictor_P Rictor Rheb_P Rheb RSK_P RSK PDK1_P PDK1 MLL_P MLL HOXA9 GBL_P GBL ERK_P ERK AKT_P AKT**
**38**	**36**	Basin Dimension Percentual: **0.04**
		**miRNA_1976 miRNA_196B mTOR_P mTOR TSC_1_2 Rictor_P Rictor Rheb_P Rheb RSK_P RSK PDK1_P PDK1 MLL_P MLL HOXA9 GBL_P GBL ERK_P ERK AKT_P AKT**
		**miRNA_1976 miRNA_196B mTOR_P mTOR TSC_1_2 Rictor_P Rictor Rheb_P Rheb RSK_P RSK PDK1_P PDK1 MLL_P MLL HOXA9 GBL_P GBL ERK_P ERK AKT_P AKT**
		**miRNA_1976 miRNA_196B mTOR_P mTOR TSC_1_2 Rictor_P Rictor Rheb_P Rheb RSK_P RSK PDK1_P PDK1 MLL_P MLL HOXA9 GBL_P GBL ERK_P ERK AKT_P AKT**
		**miRNA_1976 miRNA_196B mTOR_P mTOR TSC_1_2 Rictor_P Rictor Rheb_P Rheb RSK_P RSK PDK1_P PDK1 MLL_P MLL HOXA9 GBL_P GBL ERK_P ERK AKT_P AKT**
		**miRNA_1976 miRNA_196B mTOR_P mTOR TSC_1_2 Rictor_P Rictor Rheb_P Rheb RSK_P RSK PDK1_P PDK1 MLL_P MLL HOXA9 GBL_P GBL ERK_P ERK AKT_P AKT**
		**miRNA_1976 miRNA_196B mTOR_P mTOR TSC_1_2 Rictor_P Rictor Rheb_P Rheb RSK_P RSK PDK1_P PDK1 MLL_P MLL HOXA9 GBL_P GBL ERK_P ERK AKT_P AKT**
		**miRNA_1976 miRNA_196B mTOR_P mTOR TSC_1_2 Rictor_P Rictor Rheb_P Rheb RSK_P RSK PDK1_P PDK1 MLL_P MLL HOXA9 GBL_P GBL ERK_P ERK AKT_P AKT**
**18**	**34**	Basin Dimension Percentual: **0.03**
		**miRNA_1976 miRNA_196B mTOR_P mTOR TSC_1_2 Rictor_P Rictor Rheb_P Rheb RSK_P RSK PDK1_P PDK1 MLL_P MLL HOXA9 GBL_P GBL ERK_P ERK AKT_P AKT**
		**miRNA_1976 miRNA_196B mTOR_P mTOR TSC_1_2 Rictor_P Rictor Rheb_P Rheb RSK_P RSK PDK1_P PDK1 MLL_P MLL HOXA9 GBL_P GBL ERK_P ERK AKT_P AKT**
		**miRNA_1976 miRNA_196B mTOR_P mTOR TSC_1_2 Rictor_P Rictor Rheb_P Rheb RSK_P RSK PDK1_P PDK1 MLL_P MLL HOXA9 GBL_P GBL ERK_P ERK AKT_P AKT**
		**miRNA_1976 miRNA_196B mTOR_P mTOR TSC_1_2 Rictor_P Rictor Rheb_P Rheb RSK_P RSK PDK1_P PDK1 MLL_P MLL HOXA9 GBL_P GBL ERK_P ERK AKT_P AKT**

		**miRNA_1976 miRNA_196B mTOR_P mTOR TSC_1_2 Rictor_P Rictor Rheb_P Rheb RSK_P RSK PDK1_P PDK1 MLL_P MLL HOXA9 GBL_P GBL ERK_P ERK AKT_P AKT**
		**miRNA_1976 miRNA_196B mTOR_P mTOR TSC_1_2 Rictor_P Rictor Rheb_P Rheb RSK_P RSK PDK1_P PDK1 MLL_P MLL HOXA9 GBL_P GBL ERK_P ERK AKT_P AKT**
		**miRNA_1976 miRNA_196B mTOR_P mTOR TSC_1_2 Rictor_P Rictor Rheb_P Rheb RSK_P RSK PDK1_P PDK1 MLL_P MLL HOXA9 GBL_P GBL ERK_P ERK AKT_P AKT**
**39**	**20**	Basin Dimension Percentual: **0.02**
		**miRNA_1976 miRNA_196B mTOR_P mTOR TSC_1_2 Rictor_P Rictor Rheb_P Rheb RSK_P RSK PDK1_P PDK1 MLL_P MLL HOXA9 GBL_P GBL ERK_P ERK AKT_P AKT**
		**miRNA_1976 miRNA_196B mTOR_P mTOR TSC_1_2 Rictor_P Rictor Rheb_P Rheb RSK_P RSK PDK1_P PDK1 MLL_P MLL HOXA9 GBL_P GBL ERK_P ERK AKT_P AKT**
		**miRNA_1976 miRNA_196B mTOR_P mTOR TSC_1_2 Rictor_P Rictor Rheb_P Rheb RSK_P RSK PDK1_P PDK1 MLL_P MLL HOXA9 GBL_P GBL ERK_P ERK AKT_P AKT**
		**miRNA_1976 miRNA_196B mTOR_P mTOR TSC_1_2 Rictor_P Rictor Rheb_P Rheb RSK_P RSK PDK1_P PDK1 MLL_P MLL HOXA9 GBL_P GBL ERK_P ERK AKT_P AKT**
		**miRNA_1976 miRNA_196B mTOR_P mTOR TSC_1_2 Rictor_P Rictor Rheb_P Rheb RSK_P RSK PDK1_P PDK1 MLL_P MLL HOXA9 GBL_P GBL ERK_P ERK AKT_P AKT**
		**miRNA_1976 miRNA_196B mTOR_P mTOR TSC_1_2 Rictor_P Rictor Rheb_P Rheb RSK_P RSK PDK1_P PDK1 MLL_P MLL HOXA9 GBL_P GBL ERK_P ERK AKT_P AKT**
		**miRNA_1976 miRNA_196B mTOR_P mTOR TSC_1_2 Rictor_P Rictor Rheb_P Rheb RSK_P RSK PDK1_P PDK1 MLL_P MLL HOXA9 GBL_P GBL ERK_P ERK AKT_P AKT**
**37**	**18**	Basin Dimension Percentual: **0.02**
		**miRNA_1976 miRNA_196B mTOR_P mTOR TSC_1_2 Rictor_P Rictor Rheb_P Rheb RSK_P RSK PDK1_P PDK1 MLL_P MLL HOXA9 GBL_P GBL ERK_P ERK AKT_P AKT**
		**miRNA_1976 miRNA_196B mTOR_P mTOR TSC_1_2 Rictor_P Rictor Rheb_P Rheb RSK_P RSK PDK1_P PDK1 MLL_P MLL HOXA9 GBL_P GBL ERK_P ERK AKT_P AKT**

		**miRNA_1976 miRNA_196B mTOR_P mTOR TSC_1_2 Rictor_P Rictor Rheb_P Rheb RSK_P RSK PDK1_P PDK1 MLL_P MLL HOXA9 GBL_P GBL ERK_P ERK AKT_P AKT**
		**miRNA_1976 miRNA_196B mTOR_P mTOR TSC_1_2 Rictor_P Rictor Rheb_P Rheb RSK_P RSK PDK1_P PDK1 MLL_P MLL HOXA9 GBL_P GBL ERK_P ERK AKT_P AKT**
		**miRNA_1976 miRNA_196B mTOR_P mTOR TSC_1_2 Rictor_P Rictor Rheb_P Rheb RSK_P RSK PDK1_P PDK1 MLL_P MLL HOXA9 GBL_P GBL ERK_P ERK AKT_P AKT**
		**miRNA_1976 miRNA_196B mTOR_P mTOR TSC_1_2 Rictor_P Rictor Rheb_P Rheb RSK_P RSK PDK1_P PDK1 MLL_P MLL HOXA9 GBL_P GBL ERK_P ERK AKT_P AKT**
		**miRNA_1976 miRNA_196B mTOR_P mTOR TSC_1_2 Rictor_P Rictor Rheb_P Rheb RSK_P RSK PDK1_P PDK1 MLL_P MLL HOXA9 GBL_P GBL ERK_P ERK AKT_P AKT**
**15**	**17**	Basin Dimension Percentual: **0.02**
		**miRNA_1976 miRNA_196B mTOR_P mTOR TSC_1_2 Rictor_P Rictor Rheb_P Rheb RSK_P RSK PDK1_P PDK1 MLL_P MLL HOXA9 GBL_P GBL ERK_P ERK AKT_P AKT**
		**miRNA_1976 miRNA_196B mTOR_P mTOR TSC_1_2 Rictor_P Rictor Rheb_P Rheb RSK_P RSK PDK1_P PDK1 MLL_P MLL HOXA9 GBL_P GBL ERK_P ERK AKT_P AKT**
		**miRNA_1976 miRNA_196B mTOR_P mTOR TSC_1_2 Rictor_P Rictor Rheb_P Rheb RSK_P RSK PDK1_P PDK1 MLL_P MLL HOXA9 GBL_P GBL ERK_P ERK AKT_P AKT**
		**miRNA_1976 miRNA_196B mTOR_P mTOR TSC_1_2 Rictor_P Rictor Rheb_P Rheb RSK_P RSK PDK1_P PDK1 MLL_P MLL HOXA9 GBL_P GBL ERK_P ERK AKT_P AKT**
		**miRNA_1976 miRNA_196B mTOR_P mTOR TSC_1_2 Rictor_P Rictor Rheb_P Rheb RSK_P RSK PDK1_P PDK1 MLL_P MLL HOXA9 GBL_P GBL ERK_P ERK AKT_P AKT**
		**miRNA_1976 miRNA_196B mTOR_P mTOR TSC_1_2 Rictor_P Rictor Rheb_P Rheb RSK_P RSK PDK1_P PDK1 MLL_P MLL HOXA9 GBL_P GBL ERK_P ERK AKT_P AKT**
		**miRNA_1976 miRNA_196B mTOR_P mTOR TSC_1_2 Rictor_P Rictor Rheb_P Rheb RSK_P RSK PDK1_P PDK1 MLL_P MLL HOXA9 GBL_P GBL ERK_P ERK AKT_P AKT**
**68**	**4**	Basin Dimension Percentual: **0.00**

		**miRNA_1976 miRNA_196B mTOR_P mTOR TSC_1_2 Rictor_P Rictor Rheb_P Rheb RSK_P RSK PDK1_P PDK1 MLL_P MLL HOXA9 GBL_P GBL ERK_P ERK AKT_P AKT**
**42**	**2**	Basin Dimension Percentual: **0.00**
		**miRNA_1976 miRNA_196B mTOR_P mTOR TSC_1_2 Rictor_P Rictor Rheb_P Rheb RSK_P RSK PDK1_P PDK1 MLL_P MLL HOXA9 GBL_P GBL ERK_P ERK AKT_P AKT**
**41**	**1**	Basin Dimension Percentual: **0.00**
		**miRNA_1976 miRNA_196B mTOR_P mTOR TSC_1_2 Rictor_P Rictor Rheb_P Rheb RSK_P RSK PDK1_P PDK1 MLL_P MLL HOXA9 GBL_P GBL ERK_P ERK AKT_P AKT**
